# Nutrient‐driven growth and microbiome shifts in the brown alga *Sargassum fluitans* III

**DOI:** 10.1111/jpy.70045

**Published:** 2025-06-20

**Authors:** Tom Theirlynck, Lotte Staat, Dhaishendra Servania, Aschwin H. Engelen, Brigitta I. van Tussenbroek, Gerard Muyzer, Petra M. Visser, Linda Amaral‐Zettler

**Affiliations:** ^1^ Department of Marine Microbiology and Biogeochemistry Royal Netherlands Institute for Sea Research Den Burg/Texel the Netherlands; ^2^ Microbial Systems Ecology, Department of Freshwater and Marine Ecology, Institute for Biodiversity and Ecosystem Dynamics University of Amsterdam Amsterdam the Netherlands; ^3^ Centro de Ciências do Mar do Algarve (CCMAR/CIMAR LA), Campus de Gambelas Universidade do Algarve Faro Portugal; ^4^ CARMABI Foundation Curaçao; ^5^ Instituto de Ciencias del Mar y Limnología‐UNAM Unidad Académica de Sistemas Arrecifales Puerto Morelos Mexico

**Keywords:** brown alga, growth, microbiome, nutrient limitation, photosynthesis

## Abstract

Since 2011, holopelagic *Sargassum* has been accumulating in a region of the tropical Atlantic now referred to as the Great Atlantic *Sargassum* Belt (GASB). Among the hypothesized contributors to these accumulations are the increased inputs of nitrogen (N) and phosphorus (P) in the tropical Atlantic Ocean. Little is known about the effects of N and P additions on *Sargassum* physiology and its microbiome. We studied the effects of N, P, and NP additions on the growth, photosynthetic efficiency, and microbiome composition of *Sargassum fluitans* III in a six‐day experiment on the Caribbean Island of Curaçao. *Sargassum fluitans* III took up most nitrate and phosphate within 3 days with respective uptake rates of 0.343 and 0.0399 μmol · g^−1^ DW · h^−1^. *F*v/*F*m decreased in the control after 6 days but remained constant in nutrient treatments. Growth rates did not differ significantly among treatments, but a trend in higher growth rates in the NP treatment was discerned, suggesting a possible NP co‐limitation. The relative abundance of epiphytic Cyanobacteria such as *Schizothrix* and bacteria such as *Lentilitoribacter* increased under N and P addition, while heterotrophic *Rhodobacteraceae* decreased in abundance. Microeukaryotic communities responded with varying changes in alpha diversity, possibly steered by increased photosynthesis and growth of *S. fluitans* III or bacterial interactions. The physiological response to N and P and rapid change of the microbiome demonstrates that the studied *S. fluitans* III can quickly benefit from increased nutrient concentrations, which might contribute to its growth success in the GASB.

**Abbreviations**
ANOVA: Analysis of variance
ASV: amplicon sequencing variant
CARMABI: Caribbean Research and Management of Biodiversity foundation
CCA: canonical correspondence analysis
DCA: detrended correspondence analysis
GASB: Great Atlantic Sargassum Belt
HSD: honestly significant difference
ICP‐OES: inductively coupled plasma–optical emission spectrometry
LDA: linear discriminant analyses
LEfSe: linear discriminant analysis effect size
N: nitrogen
NMDS: non‐metric multidimensional scaling
P: phosphorus
PAM: pulse amplitude modulation
PCR: polymerase chain reaction
RGR: relative growth rate

## INTRODUCTION

The brown macroalgal genus *Sargassum* includes holopelagic species that were documented in the Sargasso Sea as early as the 15th century (Ryther, 1956). Holopelagic *Sargassum* (*Sargassum* from hereon) serves as an important ecosystem in the high seas of the Atlantic Ocean, most notably contributing to a large biodiversity of macrofauna and endemic species (Laffoley et al., 2011), and plays a role in local carbon sequestration (Hu et al., 2021). Since the beginning of 2011, the presence of *Sargassum* has increased drastically, accumulating in a region called the “Great Atlantic *Sargassum* Belt” (GASB) extending throughout the tropical and subtropical Atlantic Ocean, from the coasts of West Africa into the Gulf of Mexico (Wang et al., 2019). The taxonomic distinction of holopelagic species and morphotypes within accumulations of *Sargassum* is an ongoing debate, due to generally low molecular diversity observed among species (Álvarez‐Canali et al., 2024). Considering the genetic differences (Álvarez‐Canali et al., 2024; Amaral‐Zettler et al., 2017) and ecological/morphological value in distinguishing morphotypes (Parr, 1939; Schell et al., 2015), we here on in refer to *S. natans* with morphotypes I and VIII and *S. fluitans* morphotype III as genotypes. *Sargassum* accumulations differ in the relative abundance of their genotypes. *Sargassum natans* VIII was relatively more abundant in the GASB between 2011 and 2015 (Schell et al., 2015), but in recent years, *S. fluitans* III has become increasingly dominant in the Caribbean (García‐Sánchez et al., 2020), with variations among regions and seasons (Alleyne, Johnson, et al., 2023). When reaching the shore, *Sargassum* inundations have caused many problems in coastal ecosystems, smothering coastal communities of corals, sea turtles, and seagrasses (Louime et al., 2017) and polluting coastal waters upon decay (van Tussenbroek et al., 2017).

The recent inundations have increased scientific interest in understanding the accumulations of *Sargassum*. It has been hypothesized that the increase of *Sargassum* may be linked to rising seawater temperatures (Djakouré et al., 2017), changes in oceanic currents (Johns et al., 2020), seasonal winds and cyclones, and atmospheric depositions of Saharan dust (Oviatt et al., 2019). Moreover, various sources of nutrient inputs, most notably nitrogen (N) and phosphorus (P), have likely played essential roles in the formation of the GASB. *Sargassum* was originally believed to be mostly P‐limited in the oligotrophic environment of the Sargasso Sea (Lapointe, 1986), possibly facing a stronger nutrient depletion in oceanic regions as opposed to neritic regions bordering the Sargasso Sea (Lapointe, 1995). Since *Sargassum* started accumulating in the subtropical North Atlantic in 2011, however, anthropogenic global oceanic inputs of N and P have increased significantly, potentially supported by discharges from the Congo and Amazon rivers (Djakouré et al., 2017; Oviatt et al., 2019; Wang et al., 2019), which have skewed the nutrient availability and have likely affected the nutrient limitations of *Sargassum* in this region (Lapointe et al., 2021).

Several experiments have since demonstrated the effects of nutrient additions on the ecophysiology of *Sargassum* in the subtropical North Atlantic, although *Sargassum* is notoriously challenging to culture under controlled conditions (Magaña‐Gallegos, García‐Sánchez, et al., 2023). Magaña‐Gallegos, García‐Sánchez, et al. (2023) tested the effects of nitrate and phosphate additions on *S. natans* I and *S. fluitans* III in in situ and ex situ experiments in coastal Mexico. Although no differences in growth rates after nutrient addition were observed, they noted an increase in N:P ratios and possible P limitation. This corroborated in situ experiments in Martinique that reported a high N content in all *Sargassum* genotypes suggesting a P limitation (Changeux et al., 2023). Recently, Leemans et al. (2025) showed growth effects from combined N and P additions for *S. fluitans* III collected in the Mexican Caribbean. Still, these effects were observed only as part of a co‐limitation involving iron (Fe). Among *Sargassum* genotypes, *S. fluitans* III generally has had the highest growth rates (Changeux et al., 2023; Corbin & Oxenford, 2023; Magaña‐Gallegos, Villegas‐Muñoz, et al., 2023), highest resilience to high temperatures (Magaña‐Gallegos, Villegas‐Muñoz, et al., 2023), and the lowest light requirements without compromising growth (Vásquez‐Elizondo et al., 2024), which may explain the current dominance of *S. fluitans* III. These studies suggest that nutrient limitations in *Sargassum* likely depend on its condition and nutrient reserves, which vary among regions in the GASB, due to local abiotic fluctuations in the environment.

Current ecophysiology studies have not accounted for *Sargassum*'s rich microbiome tightly bound to the host (Hervé et al., 2021; Léger‐Pigout et al., 2024; Mendonça et al., 2024; Michotey et al., 2020; Mohapatra, 2023; Theirlynck et al., 2023; Torralba et al., 2017). Given the photoautotrophic and photoheterotrophic metabolism of some attached microbial groups, such as the Cyanobacteria and *Rhodobacteraceae* (Theirlynck et al., 2023; Torralba et al., 2017), the microbial community might respond to ambient N and P changes in the environment in these taxa. In turn, the main heterotrophic microbial groups in *Sargassum*, such as the Bacteroidetes, *Saprospiraceae*, and Vibrionales (Mendonça et al., 2024; Michotey et al., 2020; Theirlynck et al., 2023), might respond to changes in photosynthesis or the release of organic material by the macroalgal host after nutrient additions. In addition to photoautotrophic bacteria, heterotrophic bacteria contribute to N fixation, which could provide an added source of N for *Sargassum* (Léger‐Pigout et al., 2024).

Here, we characterized the influence of N and P additions on the growth, photosynthetic efficiency, and microbial community composition of *Sargassum fluitans* III in a 6‐day growth experiment conducted in Curaçao. We hypothesized a significant influence of P on the physiology and the microbiome of *S. fluitans* III, given the expected P limitation of *Sargassum* from previous experiments in the tropical North Atlantic. We aimed to show the underlying changes in nutrient uptake, growth, and photosynthesis of *S. fluitans* III and how these changes tie to the associated microbiome in a coastal environment.

## MATERIALS AND METHODS

### Sampling location and collection

**FIGURE 1:**
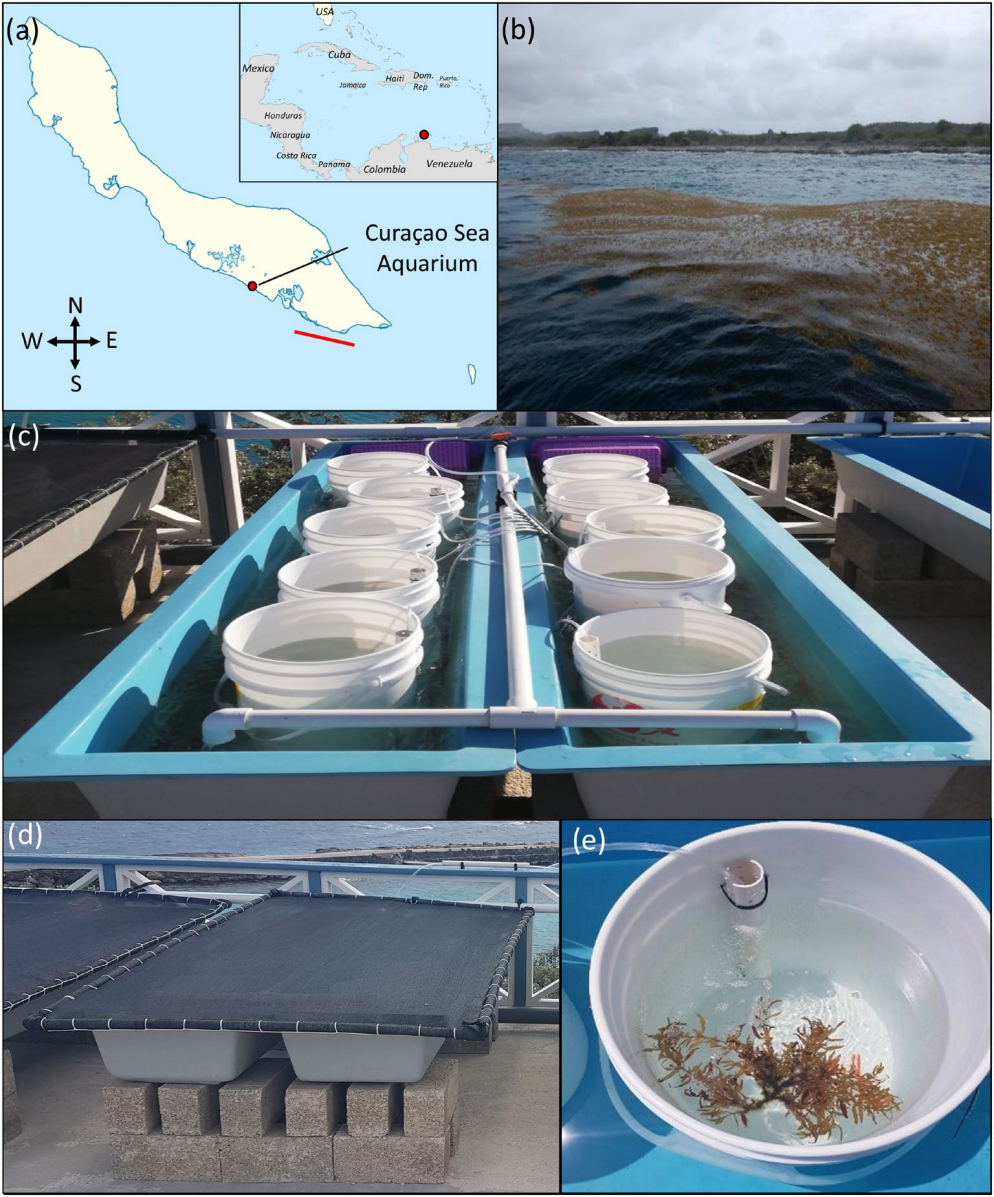
Sampling map and setup of the ecophysiology experiment. (a) Map of Curaçao and sampling area. The red line shows the area where *Sargassum fluitans* III was sampled for the experiment. The red dot shows the Curacao Sea Aquarium where the experiments were performed. (b) A raft of holopelagic *Sargassum* located in front of the coast of East Point, Curaçao, was sampled for the ecophysiology experiments. (c) In the experimental setup of the ecophysiology experiment, five buckets were placed in each raceway in continuously flowing seawater. (d) Shading cover to limit light intensity (e) Set up of the bucket system with Venturi's system of aeration to ensure water movement.

The experimental work was done on Curaçao, located in the Southern Caribbean Sea, between May and July 2022. Samples of the brown macroalga *Sargassum fluitans* III were collected during a field campaign on May 13, 2022 (Figure 1a), with the assistance of the Marine Park rangers from the Caribbean Research and Management of Biodiversity foundation (CARMABI). Samples were collected using gloved hands near Fuikbaai from a raft of *Sargassum* (Figure 1b) approximately 100–300 m off the coast of Curaçao (12°02.360′ N, 68°48.847′ W). Samples were placed in buckets (previously rinsed with ethanol) filled with seawater from the same location and closed with a lid during transport. Samples of *Sargassum* were transported to the Curaçao Sea Aquarium. *Sargassum fluitans* III was separated from the other *Sargassum* genotypes and placed in two fiberglass tanks (Dolphin Fiberglass Products, FL, United States) with dimensions of 200 × 41 × 28 cm (hereon called “raceways”) with a continuous seawater flow supplied by a Hayward 2 HP pump (Hayward Industries, NC, United States) via white PVC Type 2 pipes from the adjacent coastal environment near the Sea Aquarium. Average concentrations of dissolved nitrate (NO_3_^−^), nitrite (NO_2_^−^), and phosphate (PO_4_^3−^) in the raceways were 0.799 ± 0.043 μM NO_3_^−^, 0.075 ± 0.006 μM NO_2_^−^, and 0.064 ± 0.006 μM PO_4_^3−^. Raceways were covered with a shading fabric that maximally blocked 75% of the UV light, ensuring estimated light levels ranging from 200 to 500 μmol photons · m^−2^ · s^−1^ at noon, which is above the known light saturation range for *Sargassum* (Hanisak & Samuel, 1987; Vásquez‐Elizondo et al., 2024). The *Sargassum* clumps were maintained by ensuring maximal water flow for the *Sargassum* tissue, providing shade from sunlight, protecting it from rainfall with a cover, and removing detritus or dead tissue from the tanks daily. The *S. fluitans* III samples in raceways were acclimatized to the tank system for 17 days and used as stock specimens for the growth experiment that took place from May 30 to June 5.

### Experimental design and setup

A total of 20 buckets (25 L) were randomly divided over four raceways, accommodating five buckets per raceway (Figure 1c). A continuous seawater flow in the raceways cooled the seawater in the buckets, wherein we placed one clump of *Sargassum fluitans* III of 10–15 g wet weight per bucket and ensured that the temperature did not increase over the ambient seawater temperature of 28.5 ± 1.5°C. Each bucket was equipped with a venturi aeration tube to facilitate water movement and the continuous movement of the *S. fluitans* III clump, modeled after Magaña‐Gallegos, García‐Sánchez, et al. (2023), and buckets were shaded with a cover during the experiment (Figure 1d).

Before starting the experiment, the buckets (ø = 30 cm, height = 35 cm) were cleaned as follows: Buckets were scrubbed once using a sponge with a 10% (v/v) liquid green soap dilution (Driehoek, Amsterdam, The Netherlands), then rinsed and scrubbed three times with tap water. The buckets were subsequently rinsed once with a 1% (v/v) bleach solution, then rinsed and scrubbed once with tap water and finally washed once with seawater that was filtered through a 100‐μm nylon mesh. A hole was drilled in each bucket to fit a PVC pipe with a tie wrap that ended 5 cm above the bottom of the bucket. The PVC pipe had an aeration tube connected to an AquaForte AP‐100 air pump (SIBO Fluidra, Veghel, the Netherlands). Aeration tubes were equipped with sandstone bubble diffusers, and aeration levels could be modified by controlling valves per bucket (Figure 1e).

Buckets were filled with 18 L of 100‐μm filtered natural seawater and divided among four treatments (*n* = 5): 1) **Control**: natural seawater without nutrient enrichment (control), 2) **N**: nitrate addition (NO_3_^−^), 3) **P**: phosphate addition (PO_4_^3−^), and 4) **NP**: nitrate (NO_3_^−^) and phosphate (PO_4_^3−^) addition. For the nutrient treatments (N, P, and NP), 0.7649 g of NaNO_3_ and 0.1601 g of Na_2_HPO_4_ were weighed and dissolved in 50 mL of Milli‐Q water. Per bucket, 5 mL of these solutions were pipetted for concentrations of roughly 60 μmol · L^−1^ for the nitrate and 6 μmol · L^−1^ for the phosphate treatments, respectively. Concentrations were chosen based on an estimated 10:1 ratio for *Sargassum* N:P, based on earlier studies on Caribbean *Sargassum* and other tropical macroalgae (Diniz et al., 2011; Lapointe, 1995). Buckets were refilled daily with distilled water to compensate for water evaporation during the experiment. Raceways contained a PVC pipe of 24 cm in height to prevent overflowing the raceway and water from flowing into the buckets. The experiment lasted for 6 days, with measurements taking place on days 0, 3, and 6 (*t* = 0, *t* = 3, *t* = 6).

### Measurements of growth rates

At the start of the experiment, clumps of *Sargassum fluitans* III were inspected for healthy, non‐discolored tissue and selected from the maintenance stock in the raceways. Clumps of *S. fluitans* III were spun in a salad spinner that was cleaned with 1% (v/v) bleach and 70% (v/v) ethanol, placed in a plastic cup that was cleaned in the same manner and weighed on an On Balance CT‐250‐BK scale (Truweigh LLC, MI, United States) to determine the wet weight. A red piece of rope (ø = 2 mm) was tied to one of the apical tips of the clump to measure the length of the apical tips during the experiment, and the *S. fluitans* III clump was added to the randomly assigned experimental bucket. The apical tip length was measured by putting the *Sargassum* clump on 1 × 1 cm gritted laminated paper cleaned with 1% (v/v) bleach and 70% (v/v) ethanol, taking photos of the apical tip with a red knot and measuring the apical tip length in Image J (Schneider et al., 2012). The *Sargassum* clump was weighed, and a piece of *Sargassum* around 3–4 g wet weight was cut off and preserved for CNP and microbial analyses. Growth rates were determined by calculating the relative growth rate (RGR), based on wet weight, as doublings per day and according to Hanisak and Samuel (1987), and as increased apical tip length (Figure S3).

Photosynthetic measurements were carried out via Pulse Amplitude Modulation (PAM) on a JUNIOR‐PAM (WALZ, Effeltrich, Germany) before adding nutrients at day 0 and at the end of day 3 and 6. Buckets with *Sargassum fluitans* III samples were covered for 20 min with a black plastic bag to relax the photosystems. Before taking photosynthetic measurements, the PAM gain value was set at 3, at which the measured Ft values of *S. fluitans* III blades were between 400 and 800 mV, and the gain value was kept the same for the entire experiment. Maximum fluorescence (*F*m) was measured by giving a short light pulse and noting the photosynthetic efficiency (*F*v/*F*m). Five blades were measured per replicate and per time point (*t* = 0, *t* = 3, *t* = 6) to correct for tissue variation within the *Sargassum* clump.

### The CNP ratios and dissolved nutrient measurements

Samples of 3–4 grams of *Sargassum fluitans* III tissue were taken per replicate, stored in a plastic bag, and frozen at −20°C. The samples for CNP analysis were freeze‐dried, transported, and analyzed at the University of Amsterdam. Samples from *t* = 0 and 6 (*n* = 3) were crushed in liquid nitrogen with a mortar and pestle that were cleaned intermittently with 70% (v/v) ethanol and Milli‐Q water. Samples were ground with a ball and mill grinder (Fritsch Pulverisette 5, Idar‐Oberstein, Germany) for 4 min at 400 rpm. Total C and N contents were measured using an EL Cube CHNS elemental analyzer (Elementar, Langenselbold, Germany) following the manufacturer's guidelines. Total P was determined by destruction and inductively coupled plasma–optical emission spectrometry (ICP‐OES) with the Perkin Elmer Optima 8000 ICP‐OES and the PE S23 autosampler (PerkinElmer, MA, United States).

Seawater samples were taken with a 30‐mL syringe, filtered through a 0.2‐μm Acrodisc filter, and poured into a 6‐mL pony vial. The 0.2‐μm filter and the pony vial were rinsed three times with the sampled water beforehand to prevent contamination by previous samples. Pony vial samples were frozen and stored at −20°C until further analyses at the Royal Netherlands Institute for Sea Research (NIOZ). Concentrations of PO_4_, NO_3_, and NO_2_ (in μM) were measured on a TrAAcs 800 autoanalyzer (Bran+Luebbe, WI, United States) with minimum detection limits of 0.005 μM [PO_4_], 0.02 μM [NO_3_] and 0.004 μM [NO_2_] following the NSOP 9 guidelines (Hydes et al., 2010). Nutrient uptake rates were calculated as μmol [PO_4_] and [NO_3_] taken up per gram of dry weight of *Sargassum* per hour over the first 3 days (μmol · g^−1^ DW · h^−1^). Dry weights during the experiment were defined by the weight of samples after freeze‐drying.

### DNA extraction, PCR amplification, and Illumina NextSeq sequencing

Two to three apical tips were removed and put in a sterile Whirl‐pak™ sampling bag containing approximately 20 grams of silica gel to preserve the algal material including endophytes and epiphytes. Samples were transported to the CARMABI laboratory for whole tissue DNA extraction. The polymerase chain reaction (PCR) amplification of the 16S/18S rRNA gene V4‐5 hypervariable region was performed at the Royal Netherlands Institute for Sea Research (NIOZ) Institute. A detailed description of the extraction and bioinformatic methodology is provided in the Appendix S1.

Paired‐end Illumina NextSeq 2 × 300 bp sequencing was performed by the Gulbenkian Institute of Science Genomics Unit in Oeiras, Portugal, with the microbiome samples sequenced on two lanes, NIOZ348 and NIOZ349, resulting in 63,154,513 and 63,438,353 raw reads in total, respectively. The raw sequencing data and MIMARKS table (Appendix S2) of this study have been deposited in the European Nucleotide Archive under accession number PRJEB86257.

### Bioinformatic analyses

Sequencing data were processed in Cascabel v6.0.2 (Abdala Asbun et al., 2020) using the double‐barcoded paired workflow for the short 16S rRNA gene fragments and the double‐barcoded unpaired workflow for the longer 18S rRNA gene fragments. The CASCABEL pipeline was run in ASV (amplicon sequencing variant) mode with the same general settings described in Theirlynck et al. (2023) but using the ARB Silva database v138.1 (Quast et al., 2012) for bacterial/archaeal 16S rRNA gene taxonomy and the PR2 v5.0.0 database (Guillou et al., 2012) for the eukaryotic 18S rRNA gene taxonomic assignment. The complete CASCABEL pipeline settings for 16S rRNA and 18S rRNA gene fragment analyses can be found in the Appendix S3. After quality control, demultiplexing, and taxonomic analysis in CASCABEL, two ASV tables were created for bacteria and eukaryotes respectively that were further analyzed in R v4.3.3 (R Core Team, 2024). Any ASVs occurring fewer than 10 times across the datasets were removed. Data was decontaminated using the microDecon package v1.0.2 (McKnight et al., 2019), where reads in samples that also occurred in negative PCR controls in matching PCR batches were removed.

For the 16S rRNA gene amplicon dataset, ASVs assigned to mitochondria and plastids were removed, which amounted to ca. 68% of the total sequences in the dataset. Samples with a total count below 1000 reads were removed. The 16S rRNA gene dataset was normalized based on the median with the Phyloseq package v1.46 (McMurdie & Holmes, 2013), which resulted in 20,378 high‐quality reads on average per sample and was further analyzed based on relative abundance.

We removed 18S rRNA gene ASVs assigned to the *Sargassum* genus, which amounted to ca. 87% of the total sequences in the dataset. Samples with a total count below 100 reads were removed. The 18S rRNA gene amplicon dataset was limited, with an average read count of 1919 reads per sample, and was therefore exclusively analyzed based on ASV presence/absence and not on relative abundance.

### Statistical analyses

Statistical analyses were performed in R v4.3.3 (R Core Team, 2024). Differences in dissolved nitrate and phosphate, nutrient uptake, CNP tissue contents, RGR, apical tip length, and photosynthetic efficiency were tested for normality with Shapiro–Wilk tests and by observing the spread of the residuals. A square root transformation was used for normalization of the %N content. When a normal distribution was confirmed, the CNP contents, RGR, photosynthetic efficiency, and length were tested with two‐way analysis of variance (ANOVA) tests and subsequent Tukey honestly significant difference (HSD) post hoc tests to check for differences between all treatment × day combinations. Concentrations of dissolved nitrate and phosphate were tested with repeated measures ANOVA tests with the nlme package (Pinheiro & Bates, 2000) and subsequent pairwise *t*‐tests between treatment × day groups with a Benjamini‐Hochberg false discovery rate (FDR) correction for multiple testing. Nitrate and phosphate uptake were tested with a one‐way ANOVA test.

A non‐metric multidimensional scaling (NMDS) analysis based on Bray‐Curtis distances was performed to analyze the differences in the microbial community composition at the ASV level using the Phyloseq package, with a stress value lower than 0.15 after 100 runs. Permutational analysis of varaince (PERMANOVA) tests were done in R using the adonis2 function with the vegan package v2.6–4 (Oksanen et al., 2022) to test for statistical differences in bacterial community composition between treatment and day after checking for homogeneity of group dispersions using betadisper. Subsequent pairwise PERMANOVA tests were used to test treatment × day combinations with an FDR correction for multiple testing.

Relative abundances of the top 20 most abundant taxa of bacteria and eukaryotes were plotted with the dplyr v1.1.0 package and ggplot2 package (Wickham, 2016).

Linear discriminant analysis effect size (LEfSe) analyses were done at the bacterial genus level in MicrobiomeAnalyst (Dhariwal et al., 2017). Linear discriminant analyses (LDA) were done to determine the most changing genera throughout the experiment across treatments. The LDA scores were calculated with a *p*‐value cutoff of 0.05 in Kruskal‐Wallis tests. Subsequent pairwise *t*‐tests with an FDR correction were done for bacterial genera selected by LDA analyses.

A canonical correspondence analysis (CCA) was computed in R using the vegan package v2.6–4. First, we constructed a correlation matrix (Appendix S4 and S5) and removed co‐correlated abiotic variables of C/N, C/P, N/P, %C, NO_3_^−^, NO_2_^−^, and PO_4_^3−^. Then, a detrended correspondence analysis (DCA) was computed with the vegan package to inspect whether data was homogeneously or heterogeneously dispersed. Since data were heterogeneous according to the DCA (Appendix S6), we proceeded with a CCA to observe the relative impact of explanatory variables on the microbial distribution at *t* = 6. To test for the significance of factors in the CCA, the Mantel test was done to investigate the Spearman correlations between the relative abundance of ASVs and variables N, P, RGR, and *F*v/*F*m. An overview of the full statistical analyses and sample sizes is shown in Appendix S7.

## RESULTS

### Nutrient concentrations and growth parameters

**FIGURE 2:**
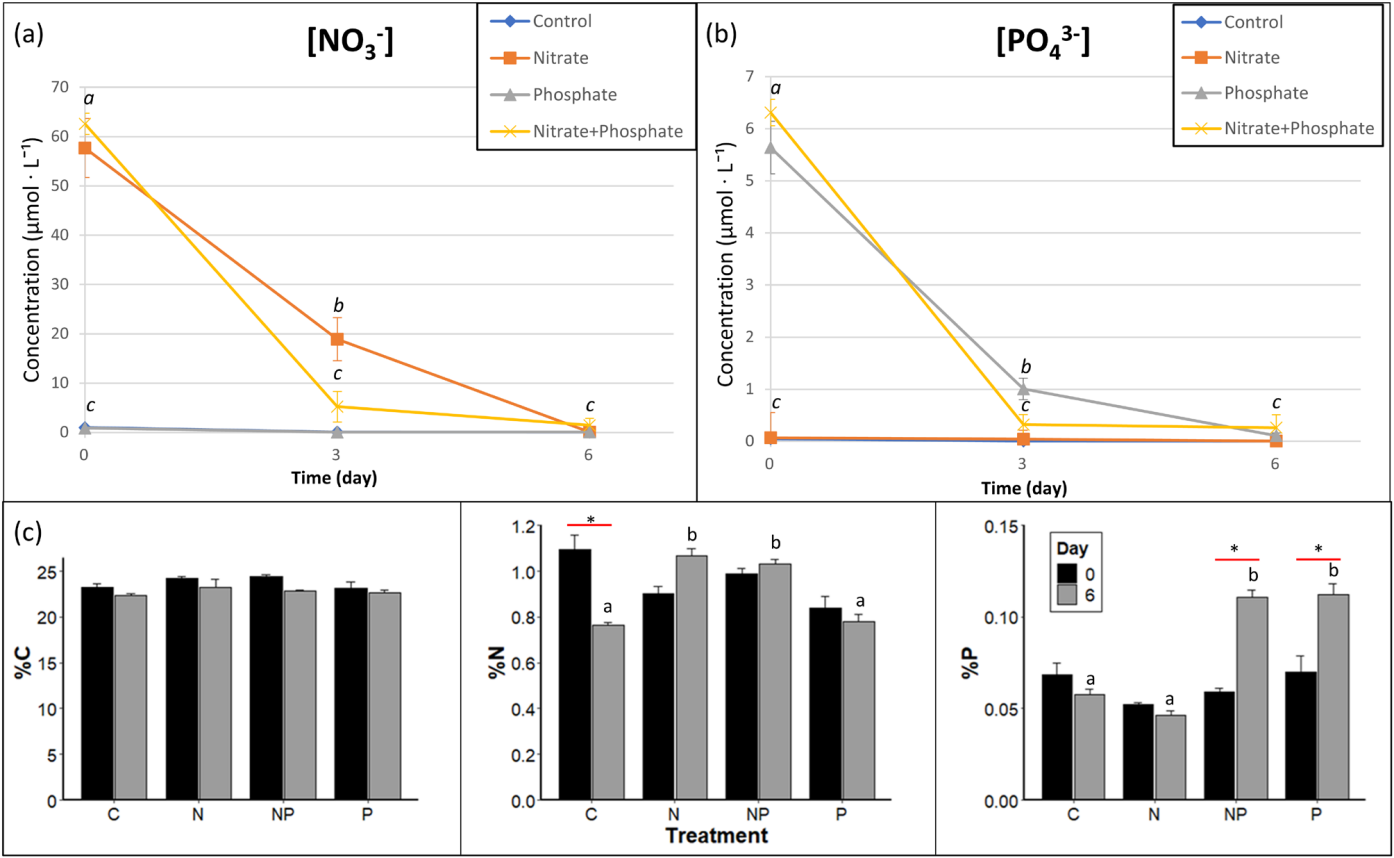
(a) Concentrations (μmol/L) of dissolved nitrate [NO_3_^−^] and (b) phosphate [PO_4_^3−^] over time, error bars show the standard error of the mean (*n* = 3), and letters indicate statistical differences at the *p* < 0.05 level (pairwise *t*‐tests) (c) Percentages of elemental carbon, nitrogen, and phosphorus on day 0 and day 6 per experimental treatment (*n* = 3). Letters indicate statistical differences among treatments and asterisks indicate differences within treatments at the *p* < 0.05 level (Tukey HSD tests). Error bars show the standard error of the mean (*n* = 3).

Dissolved nitrate and phosphate concentrations were 60.13 ± 4.06 and 5.975 ± 0.378 μmol · L^−1^, respectively, directly after nutrient addition at the start of the experiment (*t* = 0; Figure 2a,b). After 3 days, 60% to 80% of the supplied dissolved nitrate and phosphate was taken up. Concentrations of dissolved nitrate and phosphate differed from each other per treatment × day (*p* < 0.0001 & *p* < 0.0001, repeated measures ANOVA) and the NP treatment differed at *t* = 3 from the other treatments (*p* ≤ 0.048, pairwise *t*‐test). Maximum uptake rates between day 0 and 3 were 0.34 ± 0.0037 for nitrate and 0.034 ± 0.0033 μmol · g^−1^ DW · h^−1^ for phosphate in the NP treatment. At *t* = 3, the NP treatment had higher nitrate uptake rates compared to the N treatment and phosphate uptake rates compared to the P treatment (*p* ≤ 0.008, pairwise *t*‐test). After 6 days, the dissolved nitrate and phosphate were fully taken up.

The percent carbon (C) in the *Sargassum fluitans* III tissue remained constant throughout the experiment, with a non‐significant decreasing trend over the 6 days of the experiment (Figure 2c). There was a high natural variation at *t* = 0 of tissue N, with average values ranging from 0.84% to 1.09% and less for tissue P, which ranged from 0.070% to 0.052%. The elemental percentages of tissue N differed per treatment × day (*p* = 0.008, two‐way ANOVA) and were higher in the N and NP treatments than the control and P treatments after 6 days (*p* ≤ 0.048, Tukey HSD test). The elemental percentages of tissue phosphorus also differed per treatment × day (*p* = 0.003, two‐way ANOVA) and showed higher values in the P and NP treatments after 6 days compared to the control and N treatments (*p* ≤ 0.007, Tukey HSD test). Natural variation of N and P contents was high at *t* = 0. Ratios of elemental tissue CNP were 25.4 ± 1.3 (C:N), 15.9 ± 0.9 (N:P), and 400.9 ± 25.4 (C:P), and averages per treatment and day can be found in the Appendix S8.

**FIGURE 3:**
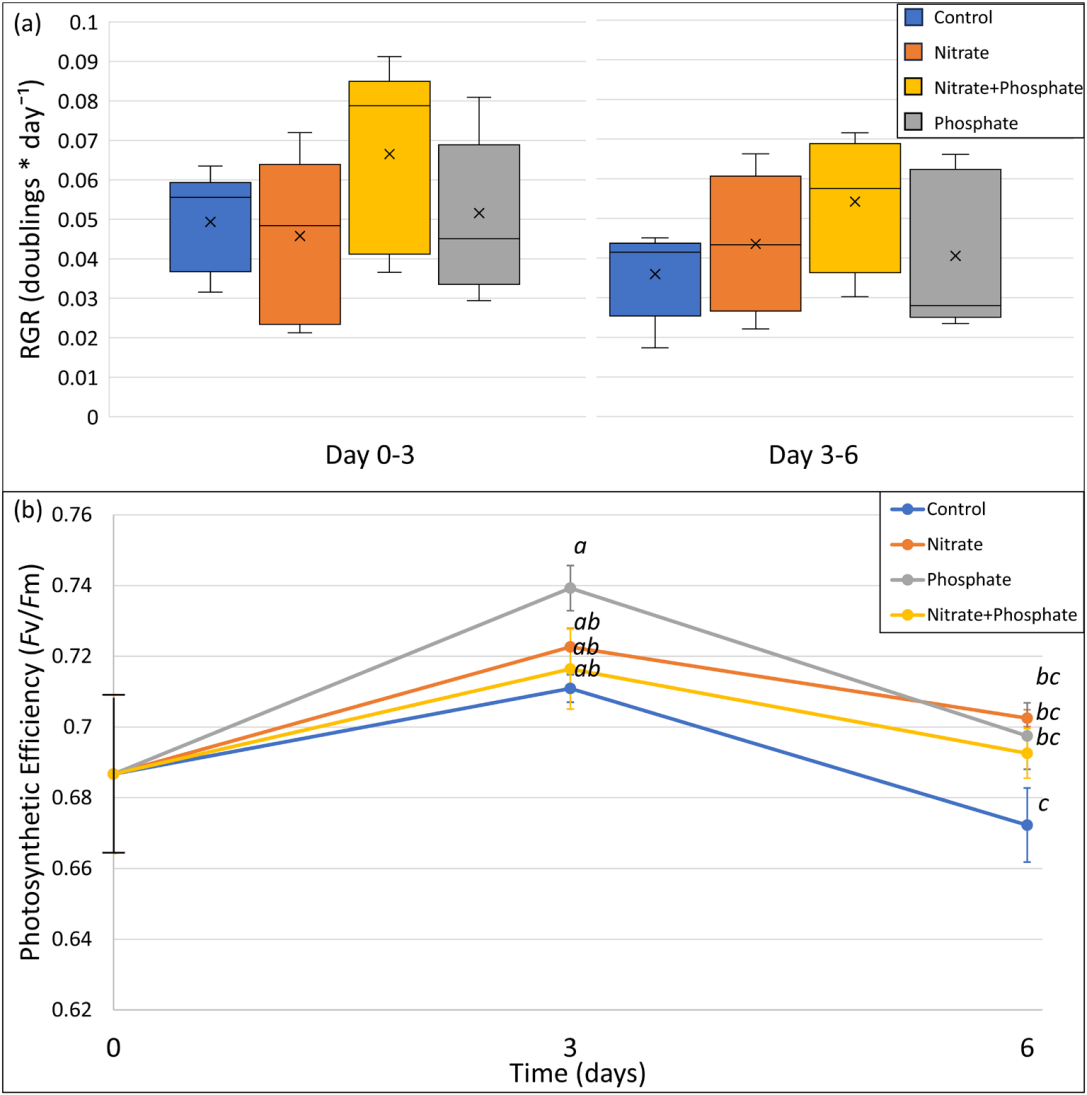
Relative Growth Rate (RGR) and photosynthetic efficiency (*F*v/*F*m) of *Sargassum fluitans* III throughout the course of the experiment for different treatments. (a) RGR is shown for 0–3 days and 3–6 days in box plots where the middle line shows the median and the cross shows the mean. (b) Average photosynthetic efficiency is shown for treatments over time with error bars displaying the standard error of the mean (*n* = 5).

The RGRs based on weight throughout the experiment were calculated for all treatments at time points *t* = 3 and *t* = 6 (Figure 3a). Replicates had relatively high variation in growth, as reflected in the spread of the boxplot. *Sargassum fluitans* III showed positive growth throughout the experiment, ranging from 0.036 to 0.067 doublings · d^−1^. The highest growth rates were measured at *t* = 3, after which growth rates decreased at *t* = 6 (*p* = 0.040, two‐way ANOVA). Adding both N and P tended to lead to higher average growth rates at both time points, but no significant differences were observed among treatments on different days (*p* = 0.527, two‐way ANOVA). The growth based on apical tip length similarly increased for all treatments at *t* = 3 but not at *t* = 6 (Appendix S9). Apical tip growth varied highly within treatments, and no significant interaction was seen between treatment and day (*p* = 0.302, two‐way ANOVA).

The photosynthetic efficiency (*F*v/*F*m) differed per treatment (*p* = 0.025, two‐way ANOVA) and per time (*p* < 0.001, two‐way ANOVA; Figure 3b). *F*v/*F*m was highest at *t* = 3, after which the *F*v/*F*m of all treatments declined at *t* = 6. The P treatment showed the highest *F*v/*F*m at *t* = 3. The control showed the lowest *F*v/*F*m during the experiment and decreased at *t* = 6 (*p* = 0.050, Tukey HSD test). After 3 and 6 days of exposure to the nutrients, the photosynthetic efficiency was the highest in the NP, N, and P treatments and the lowest in the control.

### Effect of N and P additions on the microbial community

**FIGURE 4:**
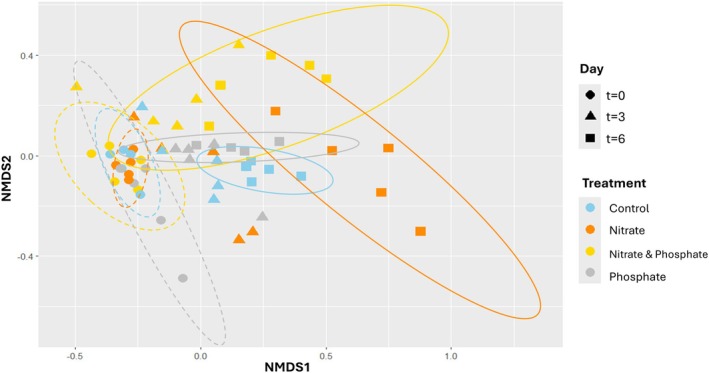
NMDS showing the differences in bacterial community beta diversity associated with different treatment × day combinations (*n* = 4–5) of *Sargassum fluitans* III at the amplicon sequence variant (ASV) level (stress factor = 0.146675). Treatments are shown with different colors and timepoints are indicated by different shapes. The 95% confidence ellipses are displayed by dashed lines at *t* = 0 and by full lines at *t* = 6.

The NMDS plot displayed differences in the bacterial community composition among treatments for different time points (Figure 4). An effect of treatment was observed over time (*p* < 0.001, PERMANOVA), with replicates clustering together tightly at *t* = 0 but dispersing at *t* = 3 and *t* = 6. Bacterial communities at *t* = 6 were different per treatment (pairwise PERMANOVA, *p* ≤ 0.028), which indicated the unique effects of additions of N and P on the bacterial community. Besides a treatment effect, we also observed an effect of time (*p* < 0.001, PERMANOVA).

**FIGURE 5:**
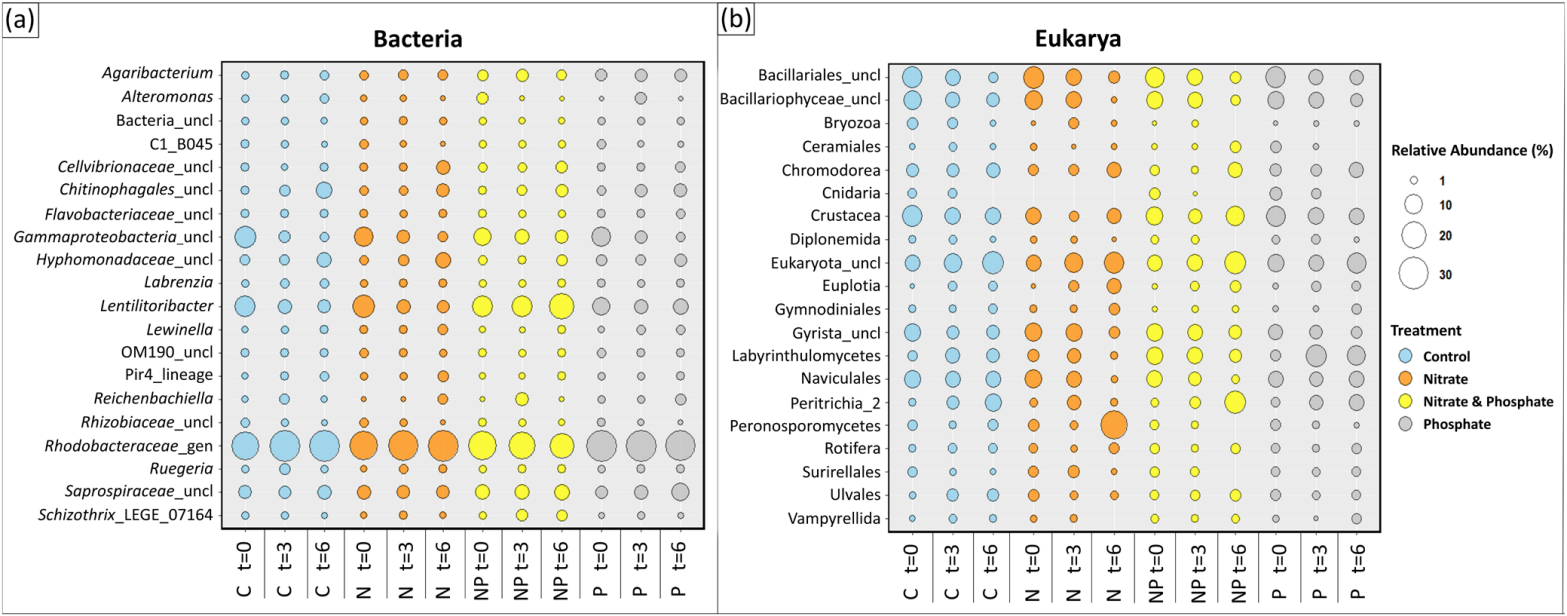
Summary of the top 20 (a) bacterial and (b) eukaryotic taxa belonging to different treatment × day combinations (*n* = 4–5) of *Sargassum fluitans* III. The size of the circle indicates the relative abundance (%) and different colors represent different treatments. Labels below the figure show the different treatment sampling timepoints. Bacterial data are based on the *relative abundance* of assigned ASVs in the dataset, while eukaryotic data are based on the *presence/absence* of the ASVs belonging to taxa indicated. The abbreviation “uncl” indicates unclassified bacteria and eukaryotes within the group.

Bacterial communities showed a high overlap of taxa, though variations in abundances were observed in the top 20 displayed genera throughout the experiment (Figure 5a). Shannon diversity indices were calculated for treatment*day groups and did not differ (*p* = 0.200, ANOVA). An unclassified genus in *Rhodobacteraceae* was the most abundant, with an average relative abundance of 25.7% across time points and treatments, with the lowest relative abundance of 18.1% in the NP treatment at day 6. Other abundant genera in the *Sargassum* microbiome were *Lentilitoribacter* (8.8%); unclassified genera in Gammaproteobacteria (5.6%), Saprospiraceae (4.6%), Chitinophagales (2.8%), and Hyphomonadaceae (2.7%); and the genus *Agaribacterium* (2.2%), which summed up to 26.6% of the microbial community. Rare microbial genera (<2%) included *Reichenbachiella*, *Ruegeria*, *Labrenzia*, *Schizothrix*, *Alteromonas*, and unclassified genera in *Cellvibrionaceae* and *Flavobacteraceae*.

Changes within sampling times and treatment were observed in the eukaryotic microbial community based on the presence and absence of ASVs within the top 20 taxa (Figure 5b). Shannon diversity indices were calculated for treatment*day groups and differed among groups (*p* < 0.001, ANOVA). A general pattern of decreasing diversity was present in the average relative abundances of Cnidaria, Diplonemida, Naviculales, Surirellales and unclassified taxa in Bacillariales, Bacillariophyceae, Crustacea, and Gyrista, which decreased throughout the experiment regardless of the treatments, summing up to about 40% of the eukaryotic community. In contrast, Chromodorea, Euplotia, Rotifera and unclassified taxa in Eukarya and Gymnodiniales displayed increases in relative abundance across treatments. Nitrogen additions had a positive influence on Peronosporomycetes after 6 days, while Peritrichia increased under NP addition. Bryozoa and Vampyrellida showed varying relative abundances under the N and P treatments over time and were not detected from the NP and N treatment, respectively, after 6 days. Furthermore, Ceremiales only increased in the NP treatment and Labyrinthulomycetes increased in the P treatment. Ulvales slightly increased in the control and NP treatments.

**FIGURE 6:**
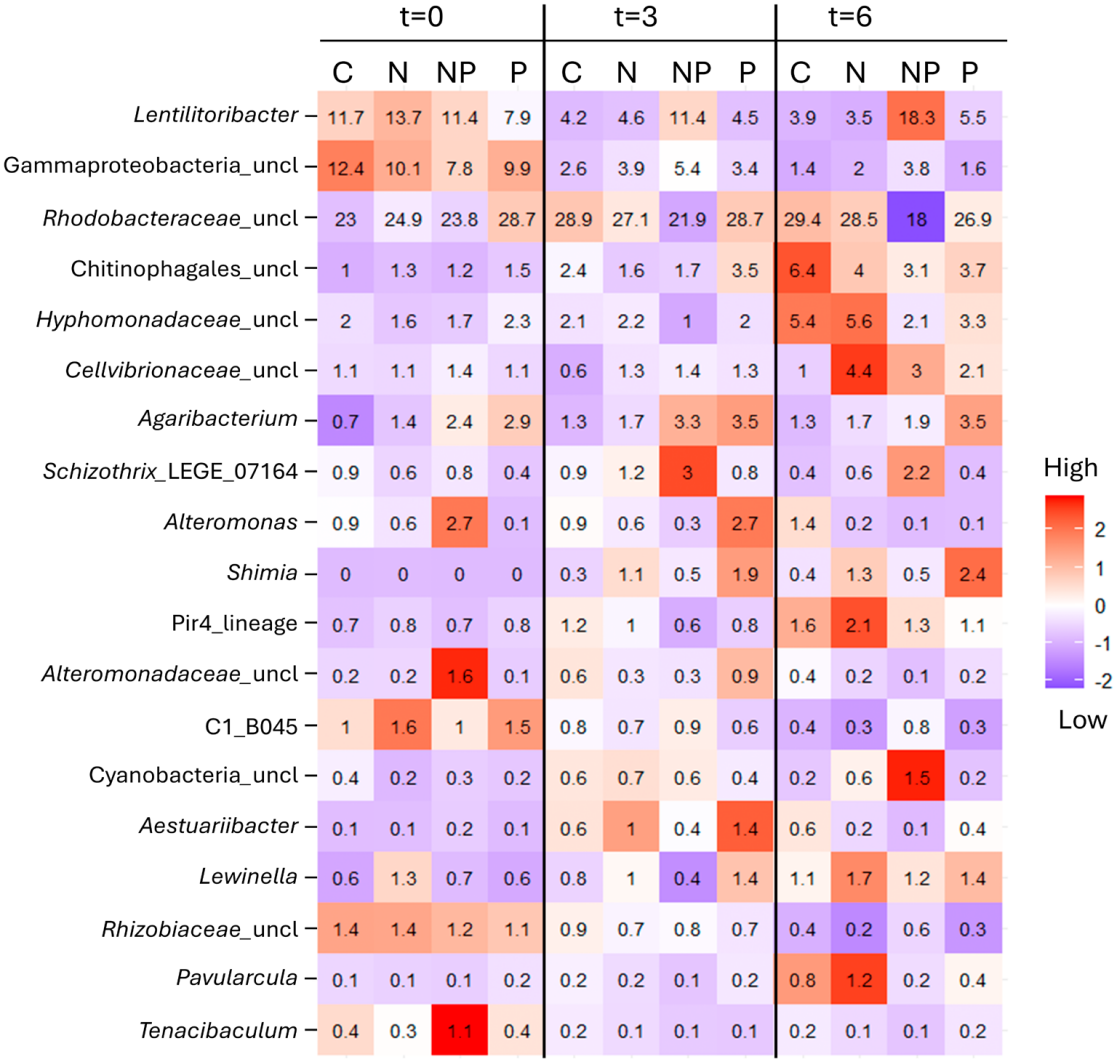
Bacterial genera selected by LEfSe (Linear discriminant analysis Effect Size) analyses with LDA scores >2.0 in *Sargassum fluitans* III. Average relative abundances of genera are shown at days 0, 3, and 6 (*t* = 0, 3, or 6) in the experiment and a heatmap indicates low‐high abundances per genus with a *z*‐score. Treatments are shown by letters: C = Control, N = Nitrate, P = Phosphate & NP = Nitrate + Phosphate. The abbreviation “uncl” indicates unclassified bacterial genera within the groups.

The LEfSe analyses identified 19 bacterial genera with an LDA‐score higher than 2.0 that differed among the experimental treatments and time points (Figure 6). Thirteen of the 19 LefSE genera were among the top 20 most abundant genera, shown in Figure 5a. *Agaribacterium*, *Alteromonas*, *Lewinella*, and an unclassified genus in *Alteromonadaceae* showed constant relative abundances across treatment day combinations (*p* ≥ 0.227, pairwise *t*‐tests), and *Aestuariibacter* fluctuated with similar relative abundances across treatments at *t* = 6 (*p* ≥ 0.123, pairwise *t*‐tests). Some identified genera showed time‐dependent changes throughout the experiment, as either a general increase in abundance across treatments from *t* = 0 to *t* = 6 for an unclassified genus in Chitinophagales (+3.1%), Pir4_lineage (+0.79%), and *Parvularcula* (+0.51%) or a decrease in abundance for C1_B045 (−0.8%), *Tenacibaculum* (−0.4%), and unclassified genera in Gammaproteobacteria (−7.9%) and *Rhizobiaceae* (−0.9%). An unclassified genus in *Hyphomonadaceae* consistently increased in the control and N treatment throughout the experiment and was more abundant than the P and NP treatment at *t* = 6 (*p* ≤ 0.004, pairwise *t*‐tests). *Shimia* increased in the P treatment over 6 days (*p* = 0.021, pairwise *t*‐test) and was more abundant than the NP treatment and control at *t* = 6 (*p* ≤ 0.044, pairwise *t*‐tests). An unclassified genus in *Cellvibrionaceae* increased on average in all nutrient treatments (+1.4%) with higher abundances in the N treatment than in the control at *t* = 6 (*p* = 0.020, pairwise *t*‐test). Most notably, however, large differences were observed in the bacterial community when both N and P were supplied. Both *Schizothrix* and an unclassified genus in Cyanobacteria increased in the NP treatment over 6 days (*p* ≤ 0.025, pairwise *t*‐tests) and were more abundant than the other treatments at *t* = 6 (*p* ≤ 0.018, pairwise *t*‐test). The genus *Lentilitoribacter* increased over 6 days in the NP treatment and was more abundant than the control at *t* = 6 (*p* = 0.037, pairwise *t*‐tests). The most abundant unclassified genus in *Rhodobacteraceae* decreased consistently in the NP treatment over 6 days (−5.8%) and was significantly less abundant than the control and N treatment at *t* = 6 (*p* = 0.045, pairwise *t*‐tests). Average relative abundances of Figures 5 and 6 are shown in Appendix S10–S12.

**FIGURE 7:**
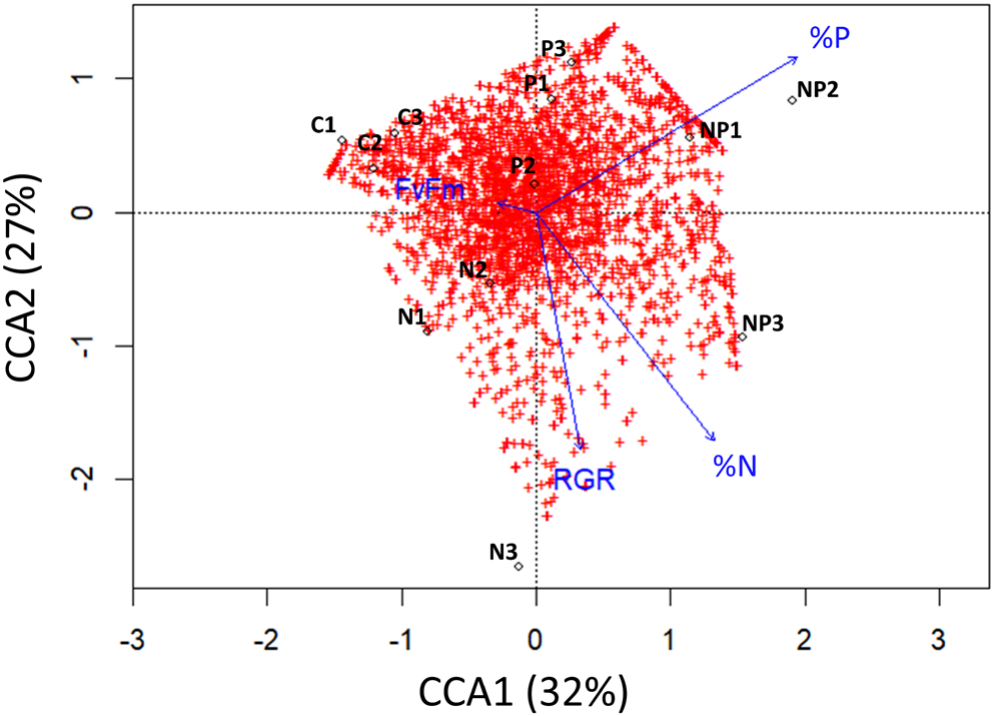
Canonical Correspondence Analysis (CCA) of the bacterial community compositions after six days influenced by %N, %P, relative growth rate (RGR), and *F*v/*F*M in *Sargassum fluitans* III. Red crosses represent all ASVs found in the dataset and black dots show the microbial community compositions of the different replicate samples (C = Control, N = Nitrate, P = Phosphate & NP = Nitrate + Phosphate). The length of the blue arrows indicates the strength of the explanatory variable for the observed microbial community composition. CCA1 explains 32% of the variance and CCA2 explains 27% of the variance.

At the ASV level, a CCA was performed to show the distribution of the bacterial community samples per treatment at day 6 and which explanatory variables best explained the experimental data (Figure 7). CCA1 explained 32% of the sample variation, and CCA2 explained 27% of the sample variation. Microbial community replicates within the control and P treatments clustered tightly, whereas a higher spread was observed in the N and NP treatments. Moreover, the CCA identified which variables best explained the bacterial community patterns, with tissue %P being best correlated with CCA1 and %N and RGR with CCA2. A Mantel test showed that the microbial community composition at the ASV level was correlated with %N (*p* = 0.004) and %P (*p* = 0.020), but not significantly correlated with the RGR (*p* = 0.097) or the *F*v/*F*m (*p* = 0.985). The bacterial community composition was mostly explained by %N and %P among our tested variables.

## DISCUSSION

With *Sargassum* accumulations increasing over the years since 2011 onward, there has been a growing importance in understanding the physiology and nutrient uptake of *Sargassum* and its microbiome. In this study, we characterized the influences of N and P additions on the growth and photosynthesis of *S. fluitans* III, which was acclimatized in a flow‐through tank system, and its microbiome over 6 days.

### Effects of N and P on the physiology of *Sargassum fluitans* III

#### N and P uptake rates

*Sargassum fluitans* III was supplied with 60.13 ± 4.06 and 5.975 ± 0.37 μmol · L^−1^ of nitrate and phosphate respectively, of which 60%–80% was taken up in the 3 days, resulting in increased tissue N and P. Although the effects of N and P on *Sargassum* have been studied before, exact nutrient uptake rates in *Sargassum* have not often been reported. Benthic *Sargassum* sp. showed higher nitrate uptake rates compared to other macroalgae (Vonk et al., 2008). High uptake rates of 5–15 and 1–4 μmol · g^1−^ DW · h^−1^ for nitrate and phosphate, respectively, have been described for some marine cyanobacteria (Den Haan et al., 2016; Solovchenko et al., 2019) and macroalgae such as *Fucus serratus* (Hurd & Dring, 1990), in which a surplus of N and P was taken up within a couple of hours, particularly in areas that were limited in N or P. Our nutrient uptake rates were lower at 0.34 and 0.034 μmol · g^−1^ DW · h^−1^ for nitrate and phosphate, respectively, although this is likely an underestimation of the maximum nutrient uptake rate, since we measured the concentrations only after 3 days and most algae take up proportionally higher concentrations in the first hours after addition (Den Haan et al., 2016; Solovchenko et al., 2019). Some species of *Sargassum* also prefer certain forms of N and P over others, such as *S. hemiphyllum*, which prefers ammonium over nitrate (Han et al., 2018), which influences the uptake mechanics and resulting *Sargassum* physiology. It would be interesting to further explore the maximum nutrient uptake of *Sargassum* by investigating the nutrient uptake of different forms of N and P in a shorter time frame.

#### *Effects of N and P on the growth and photosynthetic efficiency of* Sargassum fluitans *III*

After uptake of both N and P, there was a non‐significant trend of higher growth rates over 3 and 6 days, and the photosynthetic efficiency significantly decreased in the control after 6 days, whereas it remained constant in all nutrient addition treatments. The measured growth rates of 0.036 to 0.067 doublings · d^−1^ in our experiment align with previous ecophysiology experiments, albeit on the lower side of known growth rates for *Sargassum fluitans* III, which has reached values as high as 0.15 doublings · d^−1^ (Leemans et al., 2025). Differences in respective experimental setups and the conditions of the experimental specimens that were acclimatized in the flow‐through tank system likely contributed to the resulting differences. Most notably, acclimation at different light intensities (Vásquez‐Elizondo et al., 2024), temperatures (Corbin & Oxenford, 2023; Magaña‐Gallegos, Villegas‐Muñoz, et al., 2023), and extent of water flow (Magaña‐Gallegos, García‐Sánchez, et al., 2023) can cause variation in growth rates.

Studies on photosynthetic performance related to N and P are limited in *Sargassum*, with most studies focusing on the maximum photosynthetic capacity in P_max_ (Lapointe, 1986, 1995; Vásquez‐Elizondo et al., 2024), rather than the photosynthetic efficiency (*F*v/*F*m). Previous research showed that the photosynthetic capacity in P_max_ was positively affected by additions of N and P (Lapointe, 1995) and P separately (Lapointe, 1986), depending on the location where *Sargassum* was sampled. Nitrogen and P also play known roles in photosynthetic systems by stimulating the biosynthesis of chlorophyll and synthesis of adenosine triphosphate (ATP), respectively (Willows, 2020), explaining the general positive effects of N and P on both the photosynthetic capacity (Lapointe, 1986, 1995) and efficiency in our experiment.

#### *N and P limitations in* Sargassum fluitans *III*

The significant decrease in *F*v/*F*m in the control after 6 days and the consistent (non‐significant) trend of higher growth rates and nutrient take‐up when both N and P are supplied together suggest a possible co‐limitation of N and P in our studied specimens. Co‐limitations of N and P have been previously reported in *Sargassum* (Lapointe, 1995; Lapointe et al., 2014), with possible variations throughout regions of the Sargasso Sea. Most coastal experiments in the GASB did not indicate significant growth effects of N and P (Magaña‐Gallegos, García‐Sánchez, et al., 2023), showed indications of P limitations (Changeux et al., 2023; Lapointe, 1995), or suggested a co‐limitation of NP with Fe along the Mexico coast (Leemans et al., 2025). One explanation for a potential NP‐co‐limitation in this study is that the observed *Sargassum* N:P tissue values and NP limitation are unique to the coastal environment of Curaçao. Our supplied water in the *Sargassum* culture indicated concentrations of 0.874 ± 0.043 μM [NO_x_] and 0.064 ± 0.006 μM [PO_4_^3−^] (N:P = 13.7:1), and the S*argassum* tissue N:P ratio ranged from 12:1 to 17:1 at the start of the experiment, which was mostly in line with the 16:1 N:P Redfield ratio and does not point to a significant limitation of either N or P on their own. In contrast, the majority of *Sargassum* studies have shown increased tissue N:P ratios ranging from 21:1 to 35:1 (Changeux et al., 2023; Lapointe et al., 2021; Leemans et al., 2025; Magaña‐Gallegos, García‐Sánchez, et al., 2023), indicating a P limitation. Coastal waters of Curaçao also seem to be affected by anthropogenic nutrient inputs (Den Haan et al., 2016; Lapointe & Mallin, 2011) that could influence the availability of N and P and thus result in differing limitations opposed to previous studies.

The lack of a significant RGR of *Sargassum fluitans* III to N, P, and combined N&P addition could also suggest that there was general nutrient starvation at *t* = 6 or rather that another factor was limiting growth and photosynthesis in our studied specimens. Our study shows similarities with Leemans et al. (2025), who showed non‐significant increases in growth following the addition of N and P, comparable to our findings, although lacking in photosynthesis measurements. However, they did observe significant growth results when Fe was added with N and P. It is not unlikely that another micronutrient as Fe or vitamins (Croft et al., 2006; Leemans et al., 2025) could limit the growth of our studied *Sargassum* specimens since there is a complicated combined influence of temperature, light irradiance, and/or supplied micronutrients, which could act as cofactors (Mancuso et al., 2023). Future research should focus on testing the effect of N and P over the full range of *Sargassum* biogeography, including on the high seas, since the N:P ratio in *Sargassum*'s tissue varies across this range (Lapointe, 1995; Lapointe et al., 2021).

### Effects of N and P on the microbiome of *Sargassum fluitans* III

#### *Characterization of the microbial community of* Sargassum fluitans *III*

The microbial community of the complete apical tip was studied, including epiphytes and endophytes. Bacterial communities of *Sargassum fluitans* III were mainly formed by bacteria belonging to *Rhodobacteraceae*, *Lentilitoribacter*, Gammaproteobacteria, *Saprospiraceae*, and *Hyphomonadaceae*. The major bacterial taxa confirmed previous studies that showed a dominance of photoheterotrophic and photoautotrophic taxa within mostly the Alphaproteobacteria and Gammaproteobacteria (Hervé et al., 2021; Mendonça et al., 2024; Michotey et al., 2020; Theirlynck et al., 2023; Torralba et al., 2017). *Sargassum* releases high amounts of dissolved organic matter (Powers et al., 2019), which might promote *Rhodobacteraceae*, *Saprospiraceae*, *Hyphomonadaceae*, and Gammaproteobacteria that play roles in organic matter degradation. Some members of the *Rhodobacteraceae*, Gammaproteobacteria, and Alphaproteobacteria are phototrophs with potential roles in N fixation, which was shown in *Sargassum* by analyzing diazotrophic genes such as the *nif*H gene in the microbiome (Léger‐Pigout et al., 2024). *Hyphomonadaceae* is a family frequently associated with *Sargassum* (Mohapatra, 2023; Theirlynck et al., 2023; Torralba et al., 2017) and plays a role in nitrate removal through the process of nitrate reduction to nitrite (Abraham & Rohde, 2014).

So far only Hervé et al. (2021) have studied the eukaryotic community of *Sargassum* in a coastal Caribbean environment using molecular analyses with 515F‐926R primers. We observed that the richest groups of the eukaryotic community were members of the Crustacea, Gyrista in general, Naviculales, Bacillariophyceae, and Bacillariales. The presence of stramenopiles such as Gyrista was reported previously by Hervé et al. (2021), with stramenopiles as one of the largest groups based on richness and relative abundance. Diatom groups such as Bacillariophyceae members, Bacillariales, and Naviculales were among the most dominant epiphytes on *Sargassum*, sharing its photosynthetic niche and supporting diets of associated Nematoda or Crustacea (Monroy‐Velázquez et al., 2019). Our *Sargassum* samples also included many different groups of heterotrophic protists such as Diplonemida, Euplotia, Peritrichia, and Labyrinthulomycetes that fill differing roles, such as parasitism of the host, symbioses with bacteria, and predation on other eukaryotes or saprotrophy (Caron et al., 2012), and are extremely diverse on *Sargassum* (Baker et al., 2018). Interestingly, Cnidaria and Bryozoa have often been reported to dominate the *Sargassum* epiphytic cover (Alleyne, Neat, & Oxenford, 2023; van Tussenbroek et al., 2024), yet they are not very diverse based on our and previous molecular analyses (Hervé et al., 2021), which is in agreement with observations on these organisms (Alleyne, Neat, & Oxenford, 2023; van Tussenbroek et al., 2024). The microbial community composition at the start of the experiment thus showed a high overlap with previous studies on natural *Sargassum* microbial communities. However, there are likely tank acclimatization effects that might have shifted the studied microbiome prior to our experiment. Variation in 16S/18S primers that were used may also have led to taxonomic data differences (Lee et al., 2023).

#### Microbial changes due to N and P additions

Microbial communities of *Sargassum fluitans* III were significantly affected by the addition of N and P over 3 and 6 days, and the bacterial community composition correlated with *Sargassum* N and P contents in a Mantel test. The LEfSe analyses indicated bacterial genera that changed in abundances under the influence of combined N and P additions. Although more specific databases than SILVA could be used for higher cyanobacterial resolution (Lefler et al., 2023), several cyanobacterial genera responded differently to nutrient treatments. *Lentilitoribacter*, *Schizothrix*, and Cyanobacteria increased when both N and P were provided. *Schizothrix*, like most cyanobacteria, is often limited by macronutrients, most notably N and P (Post, 2005). Earlier studies indicated that *Schizothrix* sp. experienced elevated growth rates under higher nitrate conditions (Kuffner & Paul, 2001). *Lentilitoribacter* is a genus within the *Rhizobiaceae*, although not much is known about the particular genus, since it was only first described in 2013 (Park et al., 2013) and lacks an ecophysiological analysis. The positive response to N and P additions in our experiment indicates that these microbial genera could be NP limited in *Sargassum*. Interestingly, *Schizothrix* and *Lentilitoribacter* are potential N‐fixing bacteria (Kuffner & Paul, 2001; Kuzmanović et al., 2022). Cyanobacteria and Alphaproteobacteria are known to be important diazotrophic groups in *Sargassum* (Léger‐Pigout et al., 2024). An increase in N‐fixing bacteria under N‐rich conditions is surprising since it contrasts with the belief that the uptake of N compounds is energetically favorable to N fixation (Knapp, 2012). Nevertheless, previous studies on algal microbiomes have also shown the presence of diazotrophic bacteria in N‐rich environments (Zhang et al., 2015) and while N‐fixing can be depressed in N‐rich conditions, there remains substantial N2 fixation in marine environments (Knapp, 2012). There might, therefore, be an unknown functional importance of diazotrophs in relation to the algal host (Zhang et al., 2015) or competitive advantage to other bacteria (Knapp, 2012; Zhang et al., 2015). Furthermore, *Cellvibrionaceae* increased across nutrient additions of N, P, and NP, which was unsurprising given the copiotropic nature of many species in the genus and increased growth in nutrient‐rich conditions (Spring & Riedel, 2013). Interestingly, a decrease was observed in the abundance of *Rhodobacteraceae* only when both N and P were provided. *Rhodobacteraceae* are a family of heterotrophic organic matter degraders (Pujalte et al., 2014; Torralba et al., 2017), and a decrease could be due to possible changes in organic matter availability or the quality and composition of available organic matter. However, it could also be an indirect effect of *Lentilitoribacter*, *Schizothrix*, and other cyanobacteria that increased in abundance.

Other increases in microbial genera were observed when either N or P was provided. *Hyphomonadaceae* increased when N was provided but also increased in the control. Increases in relative abundances due to N additions could be explained due to their role in nitrate reduction (Abraham & Rohde, 2014). However, increases in *Hyphomonadaceae* in seaweed microbiomes have been previously described in response to oligotrophic conditions when N was in low concentrations (Pei et al., 2024), which was not conclusively shown in our study. *Shimia* increased in abundance when P was provided in our experiment and is capable of degradation of complex organic matter (Pujalte et al., 2014). The increase in *Shimia* may be due to its ability to directly thrive in phosphate‐rich environments, or it may indirectly benefit from shifts in community interaction that further affect organic matter quality and composition.

Although nutrient pollution sometimes results in the loss of functional diversity in host microbiomes (Mancuso et al., 2023), the bacterial community of *Sargassum fluitans* III did not decrease in diversity and was robust to heavy nutrient changes in the environment. It would be interesting to see the effects of varying concentrations of N and P and track the microbiome for a longer duration to see if there is a tipping point for this microbial shift or if the induced microbial variations lead to long‐term changes in the microbiome.

Most eukaryotic taxa decreased in richness over time, and throughout the experiment, diversity decreased in general, indicating a large acclimation effect. However, the diversity of Ceramiales (red algae) and Ulvales (green algae) increased when N and P were supplied. Nutrient pulses contribute significantly to the growth of fast‐growing species such as *Ulva prolifera* (Liu et al., 2013) and affect Ceramiales species (Ryder et al., 1999). Hence, an increase in diversity in these photosynthetic algae growing on *Sargassum* is unsurprising. The diversity of Bryozoa increased when N and P were provided, but Bryozoa were no longer detected in the NP treatment. Bryozoa potentially have a role in nutrient cycles by supplying the macroalgal host with excreted ammonium or taking up exudates, impacting algal photosynthesis when they are very abundant (Hurd et al., 2000). Although these processes might also occur on *Sargassum*, we did not observe a clear pattern confirming these studies. Further diversity increases were observed in heterotrophic groups in N‐treatments for Peritrichia and Peronosporomycetes and P treatments in saprotrophic Labyrinthulomycetes and predatory Vampyrellida. Some of these groups could benefit from the nutrient variations in the environment, but it is also likely that these groups react directly to physiological changes of *S. fluitans* III and the *Sargassum* holobiont in general. Future studies could reveal the complicated relationships that exist between the bacterial and eukaryotic microbial community of *Sargassum*, for instance through network analyses (Santi et al., 2019).

## CONCLUSIONS

We showed that *Sargassum fluitans* III quickly takes up high N and P additions in the environment. The uptake of both N and P, the decrease in photosynthetic efficiency in the control, and the trend in the growth rate, which was the highest in the NP treatment, indicate a possible combined N and P limitation in the Caribbean coastal waters of Curaçao, although there are potentially additional limiting factors, such as the availability of micronutrients. The co‐limitation of N and P is not universal across the GASB and likely depends on the local environment where *Sargassum* grows or the environments it has transited. The bacterial communities in the microbiome of *Sargassum* responded to an increase of N and P in terms of a higher relative abundance of *Lentilitoribacter* and cyanobacteria, such as *Schizothrix*, possibly facing limitations in N and P as epiphytes. In contrast, *Rhodobacteraceae* relatively decreased in the microbial community when N and P were supplied. The diversity of eukaryotic communities changed due to the additions of N and P, although future studies should further unfold the link between the host and the bacterial and eukaryotic microbiome. Although the growth of *Sargassum* and the changes in its microbiome were affected by N and P availability, they are likely part of a more intricate combined influence of factors such as temperature, micronutrients, and light intensity. Further investigation into the long‐term response of the *Sargassum* holobiont to abiotic changes in the environment could aid us in understanding the sustained expansion of the GASB.

## AUTHOR CONTRIBUTIONS

**Tom Theirlynck:** Conceptualization (equal); data curation (lead); formal analysis (lead); investigation (equal); methodology (equal); writing – original draft (lead); writing – review and editing (equal). **Lotte Staat:** Conceptualization (equal); data curation (supporting); formal analysis (supporting); investigation (equal); methodology (equal); writing – review and editing (equal). **Dhaishendra Servania:** Conceptualization (equal); data curation (supporting); formal analysis (supporting); investigation (equal); methodology (equal); writing – review and editing (equal). **Aschwin H. Engelen:** Conceptualization (equal); formal analysis (supporting); methodology (equal); writing – review and editing (equal). **Brigitta I. van Tussenbroek:** Conceptualization (equal); methodology (equal); writing – review and editing (equal). **Gerard Muyzer:** Conceptualization (equal); formal analysis (supporting); methodology (equal); writing – review and editing (equal). **Petra M. Visser:** Conceptualization (equal); methodology (equal); writing – review and editing (equal). **Linda Amaral‐Zettler:** Conceptualization (equal); formal analysis (supporting); funding acquisition (lead); methodology (equal); writing – review and editing (equal).

## Supporting information


**Appendix S1.** Extraction and PCR amplification protocol.

**Appendix S2.** MIMARKS table.

**Appendix S3.** CASCABEL pipeline settings.

**Appendix S4.** Pearson correlation coefficients of tissue content C, N, P, stoichiometric ratios of C/N, N/P and C/P, dissolved nutrient concentrations of PO4, NO2 and NO3, photosynthetic efficiency (*F*v/*F*m) and relative growth rate (RGR).
**Appendix S5.** Associated p‐values to Pearson correlation coefficients of tissue content C, N, P, stoichiometric ratios of C/N, N/P and C/P, dissolved nutrient concentrations of PO4, NO2 and NO3, photosynthetic efficiency (*F*v/*F*m) and relative growth rate (RGR).

**Appendix S6.** Detrended Correspondence Analysis (DCA) showing the distribution of the bacterial community samples of *S. fluitans* III after 6 days (*n* = 3): Control (C), Nitrate (N), Phosphate (P) and Nitrate and Phosphate (NP). Amplicon Sequence Variants (ASV) are shown with red crosses and samples of bacterial community compositions are shown with black circles.

**Appendix S7.** Overview of *p*‐values of performed statistical tests.

**Appendix S8.** Ratios of elemental C:N, C:P and C:P in *Sargassum fluitans* III for different time points during the experiment for treatments (*n* = 3): Control (C), Nitrate (N), Phosphate (P) and Nitrate and Phosphate (NP). Error bars indicate the standard error of the mean (*n* = 3).
**Appendix S9:** Accumulative growth in length of *Sargassum fluitans* III for different time points during the experiment for treatments (*n* = 5): Control, Nitrate, Phosphate and Nitrate + Phosphate. Error bars indicate the standard error of the mean (*n* = 3).

**Appendix S10.** Relative abundances (%) of displayed bacterial genera in Figures 5 and 6 for treatment day combinations (*n* = 4–5): C = control, N = nitrate, P = phosphate, and NP = nitrate and phosphate.
**Appendix S11:** Relative abundances (%) of displayed eukaryotic taxonomic groups in Figure 5 for treatment day combinations (*n* = 4–5): C = control, N = nitrate, P = phosphate and NP = nitrate and phosphate. Time (*t* = 0, 3, & 6) is displayed in days.

**Appendix S12.** Box plots of shown genera in Figure 6.

## Data Availability

Sequencing data from this study is publicly available at the European Nucleotide Archive under accession number PRJEB86257, and further data is available in article supplementary material.
