# Supplementary material for: Nutrient‐driven growth and microbiome shifts in the brown alga Sargassum fluitans III
Source: J Phycol. 2025 Jun 20;61(4):933–50. doi: 10.1111/jpy.70045 (PMC12351368; doi:10.1111/jpy.70045)
Supplement: Supplementary file 1 — Appendix S1. Extraction and PCR amplification protocol. [file JPY-61-933-s002.docx]

**Appendix S1 - Extraction and PCR amplification**

Apical ends were first crushed with a pestle and then further crushed in ZymoBIOMICS™ Lysis Solution by bead beating using a Vortex Genie 2.0 with bead beating adapter (Scientific Industries, NY, USA) for 20 minutes at 7000 rpm. DNA was extracted using the Quick DNA ZYMO Miniprep kit (ZYMO Research, CA, USA) following the manufacturer’s instructions for non-soil samples. DNA extracts were frozen at -20 ºC and transported to the Royal Netherlands Institute for Sea Research for further processing. The quality of DNA was assessed by measuring the 260/280 and 260/230 values on the Nanodrop Spectrophotometer (ThermoFisher, MA, USA). DNA concentrations were measured on a Qubit Fluorometer 2.0 (Invitrogen, CA, USA) following the high-sensitivity protocol.

PCR Amplification of the 16S/18S rRNA gene V4-5 hypervariable region was done using the 515F forward primer (5’-GTGYCAGCMGCCGCGGTAA-3’) and 926R reverse primer (5′-CCGYCAATTYMTTTRAGTTT) that capture all three domains of life (Parada et al., 2015; Quince et al., 2011). Barcoded PCR amplification was performed in triplicate in 96 well plates with the following mixture per replicate: 5 µL 5x SuperFiBuffer, 2 µL dNTP mix (2.5 µM), 0.25 µL Platinum SuperFi polymerase (Invitrogen, CA, USA), 1.25 µL of forward and reverse primer (10 µM), 1.5 µL of undiluted extracted DNA, diluted with molecular biology grade water to a total volume of 25 µL. The PCR reaction was performed with the following program settings: 30 seconds at 98 °C; 30 cycles of 10 seconds at 98 °C, 15 seconds at 50 °C, 30 seconds at 72 °C; final elongation for 5 min. at 72 °C and storage at 4 °C. PCR products were first quantified on 1% (w/v) agarose gels running for 30 min at 90 V, with GelRed staining (Biotium, ThermoFisher, USA) for 30 minutes. After successful PCR amplification, PCR products were further quantified using a 4200 TapeStation System (Agilent, CA, USA) using the D1000 manual following the manufacturer’s guidelines and then pooled in equimolar concentrations of 2 ng/µL. The resulting PCR pool was concentrated with the QIAquick PCR Purification Kit (Qiagen, Hilden, Germany) following the manufacturer's guidelines and primers were removed with an Ampure XP (Agilent, CA, USA) bead clean up with a 1:1 volume ratio of DNA to beads.
