# Supplementary material for: Nutrient‐driven growth and microbiome shifts in the brown alga Sargassum fluitans III
Source: J Phycol. 2025 Jun 20;61(4):933–50. doi: 10.1111/jpy.70045 (PMC12351368; doi:10.1111/jpy.70045)
Supplement: Supplementary file 3 — Appendix S3. CASCABEL pipeline settings. [file JPY-61-933-s009.pdf]

Paired workflow for analysis of shorter 16S rRNA fragment

Appendix S3

Amplicon Analysis Report for Library: NIOZ348

Cascabel is designed to run amplicon sequence analysis across single or multiple read libraries.

The objective of this pipeline is to create different output files which allow the user to explore data in a simple and meaningful way, as well as facilitate downstream analysis, based on the generated output files.

Another aim of **Cascabel** is also to encourage the documentation process, by creating this report in order to assure data analysis reproducibility.

Cascabel version: version 6.0.2

[redacted]

Note: Library demultiplexing has been carried out, if you have controls among your samples, please be aware that Cascabel won't perform any special operation with them. They are treated as any other sample within this workflow. Please make sure to analyze your controls with other tools, and correct your sample counts for potential contamination.

Following you can see all the steps that were taken in order to get the final results of the pipeline.

Raw Data

The raw data for this library can be found at:

- FW raw reads: Sargo_Curacao_FINAL/samples/NIOZ348/rawdata/fw.fastq

- RV raw reads: Sargo_Curacao_FINAL/samples/NIOZ348/rawdata/rv.fastq

Number of total reads: 63154513

Quality Control

Evaluate quality on raw reads.

Tool: [FastQC]

Version: **FastQC v0.11.9**

Command:

```
fastqc Sargo_Curacao_FINAL/samples/NIOZ348/rawdata/fw.fastq Sargo_Curacao_FINAL/samples/NIOZ348/rawdata/rv.fastq --
extract -o Sargo_Curacao_FINAL/samples/NIOZ348/qc/
```

You can follow the links below, in order to see the complete FastQC report:

- FastQC for sample NIOZ348_1: FQ1

- FastQC for sample NIOZ348_2: FQ2

Benchmark info:

s	max_rss	max_vms	max_uss	max_pss	io_in	io_out	mean_load
1133.67	1751.51	6156.86	1750.09	1750.11	14440.80	5.95	0.00

```
pear -f Sargo_Curacao_FINAL/samples/NIOZ348/rawdata/fw.fastq -r Sargo_Curacao_FINAL/samples/NIOZ348/rawdata/rv.fastq -t
100 -v 10 -j 620 -p 0.05 -o Sargo_Curacao_FINAL/samples/NIOZ348/peared/seqs >
Sargo_Curacao_FINAL/samples/NIOZ348/peared/seqs.assembled.fastq
```

Output files:

- Merged reads: Sargo_Curacao_FINAL/samples/NIOZ348/peared/seqs.assembled.fastq

- Log file: Sargo_Curacao_FINAL/samples/NIOZ348/peared/pear.log

Number of peared reads: 59126449=93.62%

Benchmark info:

s	max_rss	max_vms	max_uss	max_pss	io_in	io_out	mean_load
15355.15	189.48	748.29	187.70	187.72	14433.97	55867.47	0.00

Peared FastQC Analysis

Check the quality of the reads after assembly.

Tool: [FastQC]

Version: **FastQC v0.11.9**

Command:

```
fastqc Sargo_Curacao_FINAL/samples/NIOZ348/peared/seqs.assembled.fastq --extract -o
Sargo_Curacao_FINAL/samples/NIOZ348/peared/qc
```

Output files:

- FastQC report: Sargo_Curacao_FINAL/samples/NIOZ348/peared/qc/seqs.assembled_fastqc.html FQ_Report

Benchmark info:

s	max_rss	max_vms	max_uss	max_pss	io_in	io_out	mean_load
963.43	1555.06	5811.05	1540.66	1546.92	50505.61	0.54	0.00

Extract barcodes

Extract the barcodes used to identify individual samples.

Tool: [QIIME] - extract_barcodes.py

Version: **extract_barcodes.py 1.9.1**

Command:

```
extract_barcodes.py -f Sargo_Curacao_FINAL/samples/NIOZ348/peared/seqs.assembled.fastq -c barcode_paired_stitched --
bc1_len 12 --bc2_len 12 -o Sargo_Curacao_FINAL/samples/NIOZ348/barcodes/
```

Output files:

- Fastq file with barcodes: Sargo_Curacao_FINAL/samples/NIOZ348/barcodes/barcodes.fastq

- Fastq file with the reads: Sargo_Curacao_FINAL/samples/NIOZ348/barcodes/reads.fastq

Benchmark info:

s	max_rss	max_vms	max_uss	max_pss	io_in	io_out	mean_load
1817.21	119.56	5582.14	117.22	117.25	54665.61	58033.89	0.00

Correct Barcodes

Try to correct the barcode from unassigned reads. and place reads in correct orientetion.

Maximum number of mismatches **1**.

Tool: Cascabel Java program

Command:

```
java -jar Scripts/BarcodeCorrector.jar -b Sargo_Curacao_FINAL/metadata/sampleList_mergedBarcodes_NIOZ348.txt -fb
Sargo_Curacao_FINAL/samples/NIOZ348/barcodes/barcodes.fastq -fr
Sargo_Curacao_FINAL/samples/NIOZ348/barcodes/reads.fastq -m 1 -o
Sargo_Curacao_FINAL/samples/NIOZ348/barcodes/barcodes.fastq_corrected -or
Sargo_Curacao_FINAL/samples/NIOZ348/barcodes/reads.fastq_corrected -rc -x
Sargo_Curacao_FINAL/samples/NIOZ348/barcodes/sample_matrix.txt >
Sargo_Curacao_FINAL/samples/NIOZ348/barcodes/demux.log
```

Output files:

- Barcode corrected file: Sargo_Curacao_FINAL/samples/NIOZ348/barcodes/barcodes.fastq_corrected

- Reads corrected file: Sargo_Curacao_FINAL/samples/NIOZ348/barcodes/reads.fastq_corrected

- Error correction summary: Sargo_Curacao_FINAL/samples/NIOZ348/barcodes/demux.log

Benchmark info:

s	max_rss	max_vms	max_uss	max_pss	io_in	io_out	mean_load
4637.67	1428.93	38243.68	1416.66	1422.84	58823.81	58991.10	0.00

Demultiplexing

For library splitting, also known as demultiplexing, Cascabel performs several steps to assign fragments in the original as well as reverse orientation to the correct sample.

Split samples from Fastq file

Tool: [QIIME] - split_libraries_fastq.py

version: **split_libraries_fastq.py 1.9.1**

Command:

```
split_libraries_fastq.py -m Sargo_Curacao_FINAL/metadata/sampleList_mergedBarcodes_NIOZ348.txt -i
Sargo_Curacao_FINAL/samples/NIOZ348/barcodes/reads.fastq -o Sargo_Curacao_FINAL/samples/NIOZ348/splitLibs -b
Sargo_Curacao_FINAL/samples/NIOZ348/barcodes/barcodes.fastq_corrected -q 19 -r 5 --retain_unassigned_reads --phred_offset
33 --barcode_type 24
```

Benchmark info:

s	max_rss	max_vms	max_uss	max_pss	io_in	io_out	mean_load
3957.23	1884.72	6798.98	1882.23	1882.27	59101.14	30060.79	0.00

Retain assigned reads

Command:

```
cat Sargo_Curacao_FINAL/samples/NIOZ348/splitLibs/seqs.fna | grep -P -A1 "(?!>Unass)^>" | sed '/^--$/d' >
Sargo_Curacao_FINAL/samples/NIOZ348/splitLibs/seqs.no_unassigneds.fna
```

Create file with only unassigned reads

Command:

```
cat Sargo_Curacao_FINAL/samples/NIOZ348/splitLibs/seqs.fna | grep "^>Unassigned" | sed 's/>Unassigned_[0-9]* /@/g' | sed 's/
.*//' | grep -F -w -A3 -f - Sargo_Curacao_FINAL/samples/NIOZ348/peared/seqs.assembled.fastq | sed '/^--$/d'
>Sargo_Curacao_FINAL/samples/NIOZ348/splitLibs/unassigned.fastq
```

Output files:

- Text histogram with the length of the fw reads: Sargo_Curacao_FINAL/samples/NIOZ348/splitLibs/histograms.txt

- Split library log: Sargo_Curacao_FINAL/samples/NIOZ348/splitLibs/split_library_log.txt

Number of reads assigned on FW: 2839240 = 4.80% of the peared reads

Number of reads assigned on RVC: 4130781 = 6.99% of the peared reads

Generate single sample fastq files

Create single fastq files per samples (based on the raw data without applying any filtering).

Tool: Cascabel Java program

Command:

```
java -cp Scripts DemultiplexQiime --txt -a rv -b 1 -d Sargo_Curacao_FINAL/runs/MergedRun_NIOZ348_349_28-2-2024/NIOZ348_data/seqs.assigned.ori.txt -o Sargo_Curacao_FINAL/samples/NIOZ348/demultiplexed/ -r1 Sargo_Curacao_FINAL/samples/NIOZ348/rawdata/fw.fastq.gz -r2 Sargo_Curacao_FINAL/samples/NIOZ348/rawdata/fw.fastq.gz
```

Barcodes removed: 12 first bases

The demultiplexed files are located at:

- Demultiplexed directory: Sargo_Curacao_FINAL/samples/NIOZ348/demultiplexed/

- Summary file: Sargo_Curacao_FINAL/samples/NIOZ348/demultiplexed/summary.pcr.txt

Benchmark info:

s	max_rss	max_vms	max_uss	max_pss	io_in	io_out	mean_load
9063.04	1460.96	39017.88	1445.43	1451.88	22025.73	518.14	0.00

Remove primers:

Following, primers were removed from the fastq files

Remove primers from fastq files.

Primers removed: **FW** GTGYCAGCMGCCGCGGTAA **RV** CCGYCAATTYMTTTRAGTTT

Tool: [Cutadapt]

Version: **cutadapt v1.16**

Command:

```
cutadapt -g GTGYCAGCMGCCGCGGTAA -G CCGYCAATTYMTTTRAGTTT --discard-untrimmed --match-read-wildcards -O 15 -m 100 -o Sargo_Curacao_FINAL/samples/NIOZ348/demultiplexed/primer_removed/SAMPLE_1.fastq.gz -p Sargo_Curacao_FINAL/samples/NIOZ348/demultiplexed/primer_removed/SAMPLE_2.fastq.gz Sargo_Curacao_FINAL/samples/NIOZ348/demultiplexed/SAMPLE_1.fq.gz Sargo_Curacao_FINAL/samples/NIOZ348/demultiplexed/SAMPLE_2.fq.gz >> Sargo_Curacao_FINAL/samples/NIOZ348/demultiplexed/primer_removed/NIOZ348.cutadapt.log
```

The above command ran once for each single sample fastq file(s) using the mentioned primers

- Reads without primers: Sargo_Curacao_FINAL/samples/NIOZ348/demultiplexed/primer_removed

- Discarded reads (no primer): Sargo_Curacao_FINAL/samples/NIOZ348/demultiplexed/reads_discarded_primer

- Primer removal results by sample: primers_removal

Benchmark info:

s	max_rss	max_vms	max_uss	max_pss	io_in	io_out	mean_load
346.29	36.80	603.39	24.42	25.49	812.31	513.89	0.00

Sample distribution

Sample	Seqs	prc.
NIOZ348.926R.9135.9138	108966	1.56
NIOZ348.926R.9137.9140	128914	1.85
NIOZ348.926R.9139.9142	123318	1.77
NIOZ348.926R.9141.9144	121877	1.75
NIOZ348.926R.9143.9146	110426	1.58
NIOZ348.926R.9145.9148	934663	13.41
NIOZ348.926R.9147.9150	102440	1.47

Sample	Seqs	prc.
NIOZ348.926R.9149.9152	148881	2.14
NIOZ348.926R.9151.9154	77723	1.12
NIOZ348.926R.9153.9156	137968	1.98
NIOZ348.926R.9155.9158	2441703	35.03
NIOZ348.926R.9157.9160	180172	2.58
NIOZ348.926R.9159.9162	123988	1.78
NIOZ348.926R.9161.9164	488211	7.00

Sample	Seqs	prc.
NIOZ348.926R.9163.9166	97398	1.40
NIOZ348.926R.9165.9168	142505	2.04
NIOZ348.926R.9167.9170	109635	1.57
NIOZ348.926R.9169.9172	165628	2.38
NIOZ348.926R.9171.9174	76451	1.10
NIOZ348.926R.9173.9176	82623	1.19
NIOZ348.926R.9175.9178	509889	7.32

Sample	Seqs	prc.
NIOZ348.926R.9177.9180	68490	0.98
NIOZ348.926R.9179.9182	124267	1.78
NIOZ348.926R.9181.9184	79748	1.14
NIOZ348.926R.9183.9186	124872	1.79
NIOZ348.926R.9185.9188	7932	0.11
NIOZ348.926R.9187.9190	29861	0.43

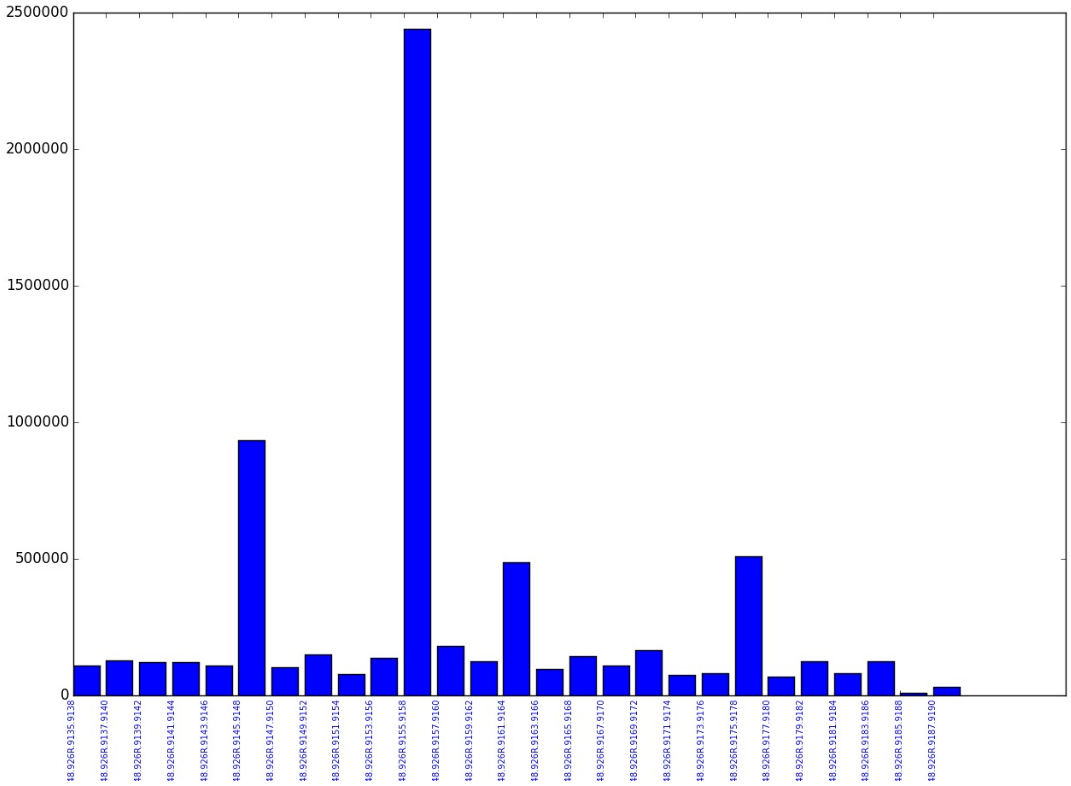

The previous chart shows the number of clean reads per sample. The bars are sorted from left to right, according to the metadata input file.

To see more details about the number of reads per sample in this library, please refer to the file: Sargo_Curacao_FINAL/runs/MergedRun_NIOZ348_349_28-2-2024/NIOZ348_data/seqs_fw_rev_filtered.dist.txt

Final counts

Following you can see the final read counts:

File description	Location	Number of reads	Prc(%) vs raw
Raw reads	Sargo_Curacao_FINAL/samples/NIOZ348/rawdata/*.fq	63154513.0	100.00%
Assembled reads	Sargo_Curacao_FINAL/samples/NIOZ348/peared/seqs.assembled.fastq	59126449.0	93.62%
Demultiplexed reads	Sargo_Curacao_FINAL/runs/MergedRun_NIOZ348_349_28-2-2024/NIOZ348_data/seqs_fw_rev_accepted.fna	6970021	11.04%
Primer removed	Sargo_Curacao_FINAL/samples/NIOZ348/demultiplexed/primer_removed/	6848549.0	10.84%

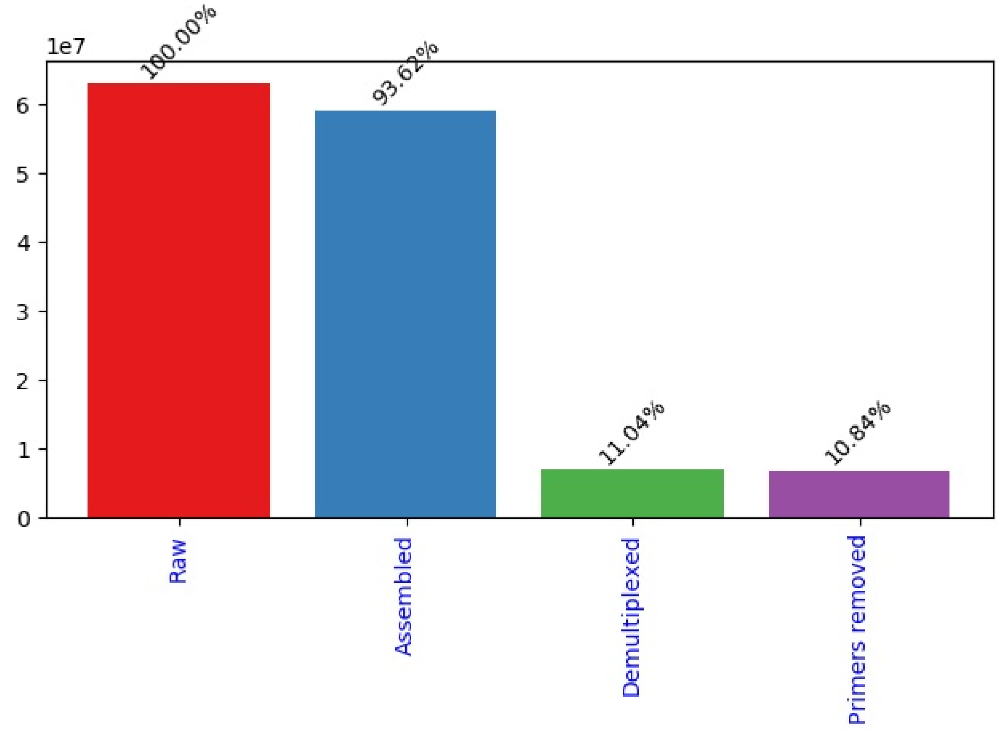

ASV report

Cascabel report on downstream analyses in combination with multiple libraries (if supplied), can be found at the following link: asv_report (Sargo_Curacao_FINAL/runs/MergedRun_NIOZ348_349_28-2-2024/asv_report_dada2.html)

References

[FastQC] (1, 2) FastQC v0.11.3. Andrews S. (2010). FastQC: a quality control tool for high throughput sequence data

[PEAR] PEAR: a fast and accurate Illumina Paired-End reAd mergeR. Zhang et al (2014) Bioinformatics 30(5): 614-620 | doi:10.1093/bioinformatics/btt593

[QIIME] (1, 2) QIIME. Caporaso JG, Kuczynski J, Stombaugh J, Bittinger K, Bushman FD, Costello EK, Fierer N, Gonzalez Pena A, Goodrich JK, Gordon JI, Huttley GA, Kelley ST, Knights D, Koenig JE, Ley RE, Lozupone CA, McDonald D, Muegge BD, Pirrung M, Reeder J, Sevinsky JR, Turnbaugh PJ, Walters WA, Widmann J, Yatsunenko T, Zaneveld J, Knight R. 2010. QIIME allows analysis of high-throughput community sequencing data. Nature Methods 7(5): 335-336.

[Cutadapt] Cutadapt v1.15 .Marcel Martin. Cutadapt removes adapter sequences from high-throughput sequencing reads. EMBnet.Journal, 17(1):10-12, May 2011. http://dx.doi.org/10.14806/ej.17.1.200

[Vsearch] Rognes T, Flouri T, Nichols B, Quince C, Mahé F. (2016) VSEARCH: a versatile open source tool for metagenomics. PeerJ 4:e2584. doi: 10.7717/peerj.2584

Amplicon Analysis Report for Library: NIOZ349

Cascabel is designed to run amplicon sequence analysis across single or multiple read libraries.

The objective of this pipeline is to create different output files which allow the user to explore data in a simple and meaningful way, as well as facilitate downstream analysis, based on the generated output files.

Another aim of **Cascabel** is also to encourage the documentation process, by creating this report in order to assure data analysis reproducibility.

Cascabel version: version 6.0.2

[redacted]

Note: Library demultiplexing has been carried out, if you have controls among your samples, please be aware that Cascabel won't perform any special operation with them. They are treated as any other sample within this workflow. Please make sure to analyze your controls with other tools, and correct your sample counts for potential contamination.

Following you can see all the steps that were taken in order to get the final results of the pipeline.

Raw Data

The raw data for this library can be found at:

- FW raw reads: Sargo_Curacao_FINAL/samples/NIOZ349/rawdata/fw.fastq

- RV raw reads: Sargo_Curacao_FINAL/samples/NIOZ349/rawdata/rv.fastq

Number of total reads: 63438353

Quality Control

Evaluate quality on raw reads.

Tool: [FastQC]

Version: **FastQC v0.11.9**

Command:

```
fastqc Sargo_Curacao_FINAL/samples/NIOZ349/rawdata/fw.fastq Sargo_Curacao_FINAL/samples/NIOZ349/rawdata/rv.fastq --extract -o Sargo_Curacao_FINAL/samples/NIOZ349/qc/
```

You can follow the links below, in order to see the complete FastQC report:

- FastQC for sample NIOZ349_1: FQ1

- FastQC for sample NIOZ349_2: FQ2

Benchmark info:

s	max_rss	max_vms	max_uss	max_pss	io_in	io_out	mean_load
1139.58	1766.67	6221.87	1764.92	1764.93	14192.34	6.01	0.00

```
pear -f Sargo_Curacao_FINAL/samples/NIOZ349/rawdata/fw.fastq -r Sargo_Curacao_FINAL/samples/NIOZ349/rawdata/rv.fastq -t 100 -v 10 -j 620 -p 0.05 -o Sargo_Curacao_FINAL/samples/NIOZ349/peared/seqs > Sargo_Curacao_FINAL/samples/NIOZ349/peared/seqs.assembled.fastq
```

Output files:

- Merged reads: Sargo_Curacao_FINAL/samples/NIOZ349/peared/seqs.assembled.fastq

- Log file: Sargo_Curacao_FINAL/samples/NIOZ349/peared/pear.log

Number of peared reads: 58219403=91.77%

Benchmark info:

s	max_rss	max_vms	max_uss	max_pss	io_in	io_out	mean_load
14800.88	189.48	748.29	187.69	187.71	14191.24	57048.09	0.00

Peared FastQC Analysis

Check the quality of the reads after assembly.

Tool: [FastQC]

Version: **FastQC v0.11.9**

Command:

```
fastqc Sargo_Curacao_FINAL/samples/NIOZ349/peared/seqs.assembled.fastq --extract -o
Sargo_Curacao_FINAL/samples/NIOZ349/peared/qc
```

Output files:

- FastQC report: Sargo_Curacao_FINAL/samples/NIOZ349/peared/qc/seqs.assembled_fastqc.html FQ_Report

Benchmark info:

s	max_rss	max_vms	max_uss	max_pss	io_in	io_out	mean_load
922.96	1639.60	6008.06	1625.45	1631.71	50647.43	0.61	0.00

Extract barcodes

Extract the barcodes used to identify individual samples.

Tool: [QIIME] - extract_barcodes.py

Version: **extract_barcodes.py 1.9.1**

Command:

```
extract_barcodes.py -f Sargo_Curacao_FINAL/samples/NIOZ349/peared/seqs.assembled.fastq -c barcode_paired_stitched --
bc1_len 12 --bc2_len 12 -o Sargo_Curacao_FINAL/samples/NIOZ349/barcodes/
```

Output files:

- Fastq file with barcodes: Sargo_Curacao_FINAL/samples/NIOZ349/barcodes/barcodes.fastq

- Fastq file with the reads: Sargo_Curacao_FINAL/samples/NIOZ349/barcodes/reads.fastq

Benchmark info:

s	max_rss	max_vms	max_uss	max_pss	io_in	io_out	mean_load
1770.36	119.57	5590.06	117.23	117.28	56504.66	59905.27	0.00

Correct Barcodes

Try to correct the barcode from unassigned reads. and place reads in correct orientetion.

Maximum number of mismatches **1**.

Tool: Cascabel Java program

Command:

```
java -jar Scripts/BarcodeCorrector.jar -b Sargo_Curacao_FINAL/metadata/sampleList_mergedBarcodes_NIOZ349.txt -fb
Sargo_Curacao_FINAL/samples/NIOZ349/barcodes/barcodes.fastq -fr
Sargo_Curacao_FINAL/samples/NIOZ349/barcodes/reads.fastq -m 1 -o
Sargo_Curacao_FINAL/samples/NIOZ349/barcodes/barcodes.fastq_corrected -or
Sargo_Curacao_FINAL/samples/NIOZ349/barcodes/reads.fastq_corrected -rc -x
Sargo_Curacao_FINAL/samples/NIOZ349/barcodes/sample_matrix.txt >
Sargo_Curacao_FINAL/samples/NIOZ349/barcodes/demux.log
```

Output files:

- Barcode corrected file: Sargo_Curacao_FINAL/samples/NIOZ349/barcodes/barcodes.fastq_corrected

- Reads corrected file: Sargo_Curacao_FINAL/samples/NIOZ349/barcodes/reads.fastq_corrected

- Error correction summary: Sargo_Curacao_FINAL/samples/NIOZ349/barcodes/demux.log

Benchmark info:

s	max_rss	max_vms	max_uss	max_pss	io_in	io_out	mean_load
12642.80	1434.34	38178.74	1420.84	1427.01	60114.55	60454.34	0.00

Demultiplexing

For library splitting, also known as demultiplexing, Cascabel performs several steps to assign fragments in the original as well as reverse orientation to the correct sample.

Split samples from Fastq file

Tool: [QIIME] - split_libraries_fastq.py

version: **split_libraries_fastq.py 1.9.1**

Command:

```
split_libraries_fastq.py -m Sargo_Curacao_FINAL/metadata/sampleList_mergedBarcodes_NIOZ349.txt -i
Sargo_Curacao_FINAL/samples/NIOZ349/barcodes/reads.fastq -o Sargo_Curacao_FINAL/samples/NIOZ349/splitLibs -b
Sargo_Curacao_FINAL/samples/NIOZ349/barcodes/barcodes.fastq_corrected -q 19 -r 5 --retain_unassigned_reads --phred_offset
33 --barcode_type 24
```

Benchmark info:

s	max_rss	max_vms	max_uss	max_pss	io_in	io_out	mean_load
4051.05	2313.12	7227.42	2310.61	2310.64	60290.75	30481.56	0.00

Retain assigned reads

Command:

```
cat Sargo_Curacao_FINAL/samples/NIOZ349/splitLibs/seqs.fna | grep -P -A1 "(?!>Unass)^>" | sed '/^--$/d' >
Sargo_Curacao_FINAL/samples/NIOZ349/splitLibs/seqs.no_unassigneds.fna
```

Create file with only unassigned reads

Command:

```
cat Sargo_Curacao_FINAL/samples/NIOZ349/splitLibs/seqs.fna | grep "^>Unassigned" | sed 's/>Unassigned_[0-9]* /@/g' | sed 's/
.*//' | grep -F -w -A3 -f - Sargo_Curacao_FINAL/samples/NIOZ349/peared/seqs.assembled.fastq | sed '/^--$/d'
>Sargo_Curacao_FINAL/samples/NIOZ349/splitLibs/unassigned.fastq
```

Output files:

- Text histogram with the length of the fw reads: Sargo_Curacao_FINAL/samples/NIOZ349/splitLibs/histograms.txt

- Split library log: Sargo_Curacao_FINAL/samples/NIOZ349/splitLibs/split_library_log.txt

Number of reads assigned on FW: 5500157 = 9.45% of the peared reads

Number of reads assigned on RVC: 7612181 = 13.07% of the peared reads

Generate single sample fastq files

Create single fastq files per samples (based on the raw data without applying any filtering).

Tool: Cascabel Java program

Command:

```
java -cp Scripts DemultiplexQiime --txt -a rv -b 1 -d Sargo_Curacao_FINAL/runs/MergedRun_NIOZ348_349_28-2-2024/NIOZ349_data/seqs.assigned.ori.txt -o Sargo_Curacao_FINAL/samples/NIOZ349/demultiplexed/ -r1 Sargo_Curacao_FINAL/samples/NIOZ349/rawdata/fw.fastq.gz -r2 Sargo_Curacao_FINAL/samples/NIOZ349/rawdata/fw.fastq.gz
```

Barcodes removed: 12 first bases

The demultiplexed files are located at:

- Demultiplexed directory: Sargo_Curacao_FINAL/samples/NIOZ349/demultiplexed/

- Summary file: Sargo_Curacao_FINAL/samples/NIOZ349/demultiplexed/summary.pcr.txt

Benchmark info:

s	max_rss	max_vms	max_uss	max_pss	io_in	io_out	mean_load
25987.01	1324.73	38620.88	1307.11	1313.55	1464.05	961.66	0.00

Remove primers:

Following, primers were removed from the fastq files

Remove primers from fastq files.

Primers removed: **FW** GTGYCAGCMGCCGCGGTAA **RV** CCGYCAATTYMTTTRAGTTT

Tool: [Cutadapt]

Version: **cutadapt v1.16**

Command:

```
cutadapt -g GTGYCAGCMGCCGCGGTAA -G CCGYCAATTYMTTTRAGTTT --discard-untrimmed --match-read-wildcards -O 15 -m 100 -o Sargo_Curacao_FINAL/samples/NIOZ349/demultiplexed/primer_removed/SAMPLE_1.fastq.gz -p Sargo_Curacao_FINAL/samples/NIOZ349/demultiplexed/primer_removed/SAMPLE_2.fastq.gz Sargo_Curacao_FINAL/samples/NIOZ349/demultiplexed/SAMPLE_1.fq.gz Sargo_Curacao_FINAL/samples/NIOZ349/demultiplexed/SAMPLE_2.fq.gz >> Sargo_Curacao_FINAL/samples/NIOZ349/demultiplexed/primer_removed/NIOZ349.cutadapt.log
```

The above command ran once for each single sample fastq file(s) using the mentioned primers

- Reads without primers: Sargo_Curacao_FINAL/samples/NIOZ349/demultiplexed/primer_removed

- Discarded reads (no primer): Sargo_Curacao_FINAL/samples/NIOZ349/demultiplexed/reads_discarded_primer

- Primer removal results by sample: primers_removal

Benchmark info:

s	max_rss	max_vms	max_uss	max_pss	io_in	io_out	mean_load
659.02	36.51	603.39	24.31	25.38	1016.92	1005.76	0.00

Sample distribution

Sample	Seqs	prc.
NIOZ349.926R.0001.0034	255006	1.94
NIOZ349.926R.0003.0036	265439	2.02
NIOZ349.926R.0005.0038	221104	1.69
NIOZ349.926R.0007.0040	288754	2.20
NIOZ349.926R.0009.0042	276955	2.11
NIOZ349.926R.0011.0044	173533	1.32
NIOZ349.926R.0013.0046	144389	1.10
NIOZ349.926R.0015.0048	151540	1.16
NIOZ349.926R.0017.0050	139138	1.06
NIOZ349.926R.0019.0052	60443	0.46
NIOZ349.926R.0021.0054	154290	1.18
NIOZ349.926R.0023.0056	183947	1.40
NIOZ349.926R.0025.0058	110744	0.84
NIOZ349.926R.0027.0060	132366	1.01
NIOZ349.926R.0029.0062	166225	1.27
NIOZ349.926R.0031.0064	163786	1.25
NIOZ349.926R.0033.0066	182624	1.39
NIOZ349.926R.0035.0068	131618	1.00
NIOZ349.926R.0037.0070	157776	1.20

Sample	Seqs	prc.
NIOZ349.926R.0039.0072	217305	1.66
NIOZ349.926R.0041.0074	119821	0.91
NIOZ349.926R.0043.0076	141184	1.08
NIOZ349.926R.0045.0078	166573	1.27
NIOZ349.926R.0047.0080	195909	1.49
NIOZ349.926R.0049.0082	252118	1.92
NIOZ349.926R.0051.0084	160587	1.22
NIOZ349.926R.0053.0086	77797	0.59
NIOZ349.926R.0055.0088	169513	1.29
NIOZ349.926R.0057.0090	160512	1.22
NIOZ349.926R.0059.0092	189474	1.45
NIOZ349.926R.0061.0094	83965	0.64
NIOZ349.926R.0063.0096	106260	0.81
NIOZ349.926R.0065.0098	202348	1.54
NIOZ349.926R.0067.0100	188764	1.44
NIOZ349.926R.0069.0102	165041	1.26
NIOZ349.926R.0071.0104	144604	1.10
NIOZ349.926R.0073.0106	117393	0.90
NIOZ349.926R.0075.0108	210882	1.61

Sample	Seqs	prc.
NIOZ349.926R.0077.0110	283531	2.16
NIOZ349.926R.0079.0112	251138	1.92
NIOZ349.926R.0081.0114	217789	1.66
NIOZ349.926R.0083.0116	172852	1.32
NIOZ349.926R.0085.0118	189780	1.45
NIOZ349.926R.0087.0120	193783	1.48
NIOZ349.926R.0089.0122	145756	1.11
NIOZ349.926R.0091.0124	190173	1.45
NIOZ349.926R.0093.0126	153713	1.17
NIOZ349.926R.0095.0128	165644	1.26
NIOZ349.926R.0097.0130	164516	1.25
NIOZ349.926R.0099.0132	176448	1.35
NIOZ349.926R.0101.0134	168864	1.29
NIOZ349.926R.0103.0136	193010	1.47
NIOZ349.926R.0105.0138	151479	1.16
NIOZ349.926R.0107.0140	180578	1.38
NIOZ349.926R.0109.0142	150988	1.15
NIOZ349.926R.0111.0144	132541	1.01
NIOZ349.926R.0113.0146	173350	1.32

Sample	Seqs	prc.
NIOZ349.926R.0115.0148	238518	1.82
NIOZ349.926R.0117.0150	180856	1.38
NIOZ349.926R.0119.0152	210359	1.60
NIOZ349.926R.0121.0154	126284	0.96
NIOZ349.926R.0123.0156	106755	0.81
NIOZ349.926R.0125.0158	113049	0.86
NIOZ349.926R.0127.0160	42262	0.32
NIOZ349.926R.0129.0162	122183	0.93
NIOZ349.926R.0131.0164	194006	1.48
NIOZ349.926R.0133.0166	152106	1.16
NIOZ349.926R.0135.0168	138952	1.06
NIOZ349.926R.0137.0170	123230	0.94
NIOZ349.926R.0139.0172	151929	1.16
NIOZ349.926R.0141.0174	160802	1.23
NIOZ349.926R.0143.0176	204813	1.56
NIOZ349.926R.0145.0178	210366	1.60
NIOZ349.926R.0147.0180	128789	0.98
NIOZ349.926R.0191.0032	144173	1.10
NIOZ349.926R.0231.0200	202276	1.54

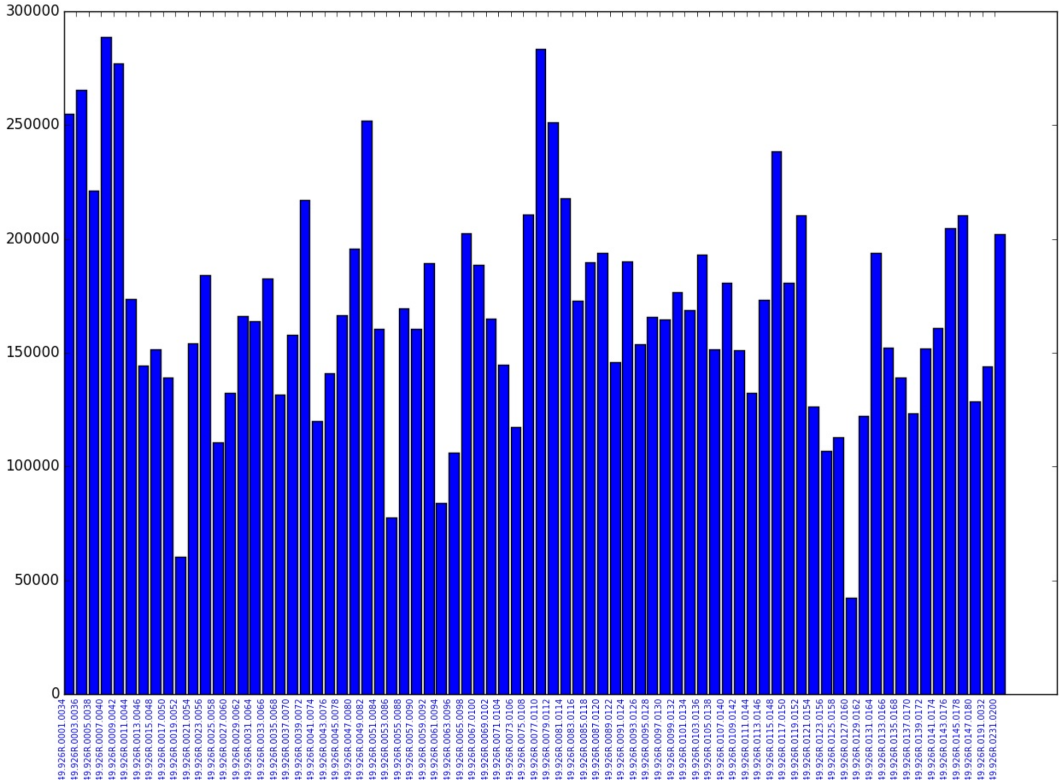

The previous chart shows the number of clean reads per sample. The bars are sorted from left to right, according to the metadata input file.

To see more details about the number of reads per sample in this library, please refer to the file: Sargo_Curacao_FINAL/runs/MergedRun_NIOZ348_349_28-2-2024/NIOZ349_data/seqs_fw_rev_filtered.dist.txt

Final counts

Following you can see the final read counts:

File description	Location	Number of reads	Prc(%) vs raw
Raw reads	Sargo_Curacao_FINAL/samples/NIOZ349/rawdata/*.fq	63438353.0	100.00%
Assembled reads	Sargo_Curacao_FINAL/samples/NIOZ349/peared/seqs.assembled.fastq	58219403.0	91.77%
Demultiplexed reads	Sargo_Curacao_FINAL/runs/MergedRun_NIOZ348_349_28-2-2024/NIOZ349_data/seqs_fw_rev_accepted.fna	13112338	20.67%
Primer removed	Sargo_Curacao_FINAL/samples/NIOZ349/demultiplexed/primer_removed/	12837368.0	20.24%

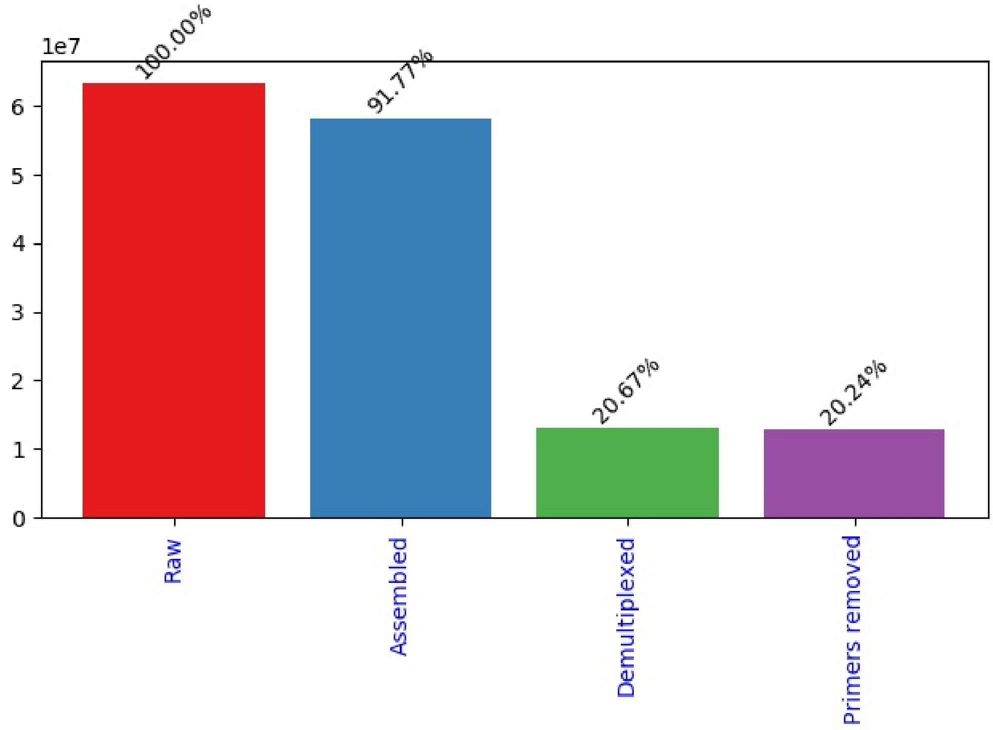

ASV report

Cascabel report on downstream analyses in combination with multiple libraries (if supplied), can be found at the following link: asv_report (Sargo_Curacao_FINAL/runs/MergedRun_NIOZ348_349_28-2-2024/asv_report_dada2.html)

References

[FastQC] (1, 2) FastQC v0.11.3. Andrews S. (2010). FastQC: a quality control tool for high throughput sequence data

[PEAR] PEAR: a fast and accurate Illumina Paired-End reAd mergeR. Zhang et al (2014) Bioinformatics 30(5): 614-620 | doi:10.1093/bioinformatics/btt593

[QIIME] (1, 2) QIIME. Caporaso JG, Kuczynski J, Stombaugh J, Bittinger K, Bushman FD, Costello EK, Fierer N, Gonzalez Pena A, Goodrich JK, Gordon JI, Huttley GA, Kelley ST, Knights D, Koenig JE, Ley RE, Lozupone CA, McDonald D, Muegge BD, Pirrung M, Reeder J, Sevinsky JR, Turnbaugh PJ, Walters WA, Widmann J, Yatsunenko T, Zaneveld J, Knight R. 2010. QIIME allows analysis of high-throughput community sequencing data. Nature Methods 7(5): 335-336.

[Cutadapt] Cutadapt v1.15 .Marcel Martin. Cutadapt removes adapter sequences from high-throughput sequencing reads. EMBnet.Journal, 17(1):10-12, May 2011. http://dx.doi.org/10.14806/ej.17.1.200

[Vsearch] Rognes T, Flouri T, Nichols B, Quince C, Mahé F. (2016) VSEARCH: a versatile open source tool for metagenomics. PeerJ 4:e2584. doi: 10.7717/peerj.2584

Amplicon Analysis Report

CASCABEL is designed to run amplicon sequence analysis across single or multiple read libraries. This report consists of the ASV table creation and taxonomic assignment for all the combined accepted reads of given samples or libraries, if multiple.

Cascabel version: version 6.0.2

[redacted]

Filter and Trim

Once that all the individual libraries were demultiplexed, the fastq files from all the samples for all the libraries were processed together.

The filter and trimming steps were both performed with the **filterAndTrim()** function from the R package dada2, according to user parameters.

Tool: dada2

Version: **[1] '1.24.0'**

Function: filterAndTrim()

Max Expected Errors (maxEE) FW: 3

Max Expected Errors (maxEE) RV: 5

Forward read truncation: 265

Reverse read truncation: 255

Command:

```
Scripts/asvFilter.R $PWD T 265 255 3 5 20 ,truncQ=2, rm.phix=TRUE Sargo_Curacao_FINAL/runs/MergedRun_NIOZ348_349_28-2-2024/asv/filter_summary.out
```

Output file:

- Filtered fastq files: Sargo_Curacao_FINAL/runs/MergedRun_NIOZ348_349_28-2-2024/<Library>/demultiplexed/filtered/

- Summary: Sargo_Curacao_FINAL/runs/MergedRun_NIOZ348_349_28-2-2024/asv/filter_summary.out

Note: To speed up downstream computation, consider tightening maxEE. If too few reads are passing the filter, consider relaxing maxEE, perhaps especially on the reverse reads.

Make sure that your forward and reverse reads overlap after length truncation.

Benchmark info:

s	max_rss	max_vms	max_uss	max_pss	io_in	io_out	mean_load
3862.28	29169.59	40346.85	28065.50	28122.37	1520.41	1263.10	0.00

Amplicon Sequence Variants

In order to identify ASVs, dada2 workflow require to execute several steps. Following a summary of these steps and its main parameters.

Tool: dada2

Version: **[1] '1.24.0'**

Learn errors

The first step after filtering the reads is to learn the errors from the fastq files.

Function: learnErrors(filteredFQ)

Error plots:

- FW reads error plot:: Sargo_Curacao_FINAL/runs/MergedRun_NIOZ348_349_28-2-2024/asv/fw_err.pdf

- RV reads error plot:: Sargo_Curacao_FINAL/runs/MergedRun_NIOZ348_349_28-2-2024/asv/rv_err.pdf

ASV inference

The amplicon sequence variant identification consists of a high resolution sample inference from the amplicon data using the learned errors.

Function: dada(filteredFQ, errors, pool='pseudo')

Merge pairs

In this step, forward and reverse reads are paired in order to create full denoised sequences.

Function: mergePairs(dadaF, dadaR)

Min overlap: 12

Max mismatch: 0

Length filtering

Sequences that are much longer or shorter than expected may be the result of non-specific priming.

- Shortest length: 220

- Longest length: 510

Remove chimeras

Sequence variants identified as bimeric are removed, and a bimera-free collection of unique sequences is generated.

Function: removeBimeraDenovo()

Method: consensus

Output files:

- Representative ASV sequences: Sargo_Curacao_FINAL/runs/MergedRun_NIOZ348_349_28-2-2024/asv/representative_seq_set.fasta

The total number of different ASVs is: **65154**

Assign taxonomy

Given a set of sequences, assign the taxonomy of each sequence.

Tool: RDP

Function: assignTaxonomy() *implementation of RDP Classifier within dada2*

Reference database: /export/data01/databases/silva/r138.1/dada2/silva_nr99_v138.1_train_set.fa.gz

Species information: The *'add species'* (add_sps) option from the configuration file is set to **false**. Set it to **true** and supply a *species database* if you want to add species-level annotation to the taxonomic table.

The percentage of successfully assigned ASVs is: **99.95%**

Output file:

- ASV taxonomy assignation: Sargo_Curacao_FINAL/runs/MergedRun_NIOZ348_349_28-2-2024/asv/taxonomy_dada2/representative_seq_set_tax_assignments.txt

The previous steps were performed within a Cascabel R script according to the following command:

Command

```
Scripts/asvDada2.R $PWD pseudo 20 T selfConsist=FALSE Sargo_Curacao_FINAL/runs/MergedRun_NIOZ348_349_28-2-2024/asv/ 220 510 10 T /export/data01/databases/silva/r138.1/dada2/silva_nr99_v138.1_train_set.fa.gz /export/data01/databases/silva/r138.1/dada2/silva_species_assignment_v138.1.fa.gz T minBoot=80 12 0 allowMultiple=TRUE
```

Benchmark info:

s	max_rss	max_vms	max_uss	max_pss	io_in	io_out	mean_load
75006.72	112016.34	128030.10	112004.24	112007.53	1227.57	37.59	0.00

Make ASV table

Tabulates the number of times an ASV is found in each sample, and adds the taxonomic predictions for each ASV in the last column.

Command:

```
cat Sargo_Curacao_FINAL/runs/MergedRun_NIOZ348_349_28-2-2024/asv/taxonomy_dada2/representative_seq_set_tax_assignments.txt | awk 'NR==FNR{if(NR>1){tax=$2;for(i=3;i<=NF;i++){tax=tax";"$i};h[$1]=tax;}next;} {if(FNR==1){print $0"ttaxonomy"}else{print $0"t"h[$1]}' - Sargo_Curacao_FINAL/runs/MergedRun_NIOZ348_349_28-2-2024/asv/asv_table.txt > Sargo_Curacao_FINAL/runs/MergedRun_NIOZ348_349_28-2-2024/asv/taxonomy_dada2/asvTable.txt
```

Output file:

- ASV table: Sargo_Curacao_FINAL/runs/MergedRun_NIOZ348_349_28-2-2024/asv/taxonomy_dada2/asvTable.txt

Benchmark info:

s	max_rss	max_vms	max_uss	max_pss	io_in	io_out	mean_load
0.32	1.39	110.64	0.24	0.25	0.00	0.00	0.00

Convert ASV table

Convert from txt to the BIOM table format.

Tool: [BIOM]

Version: **biom, version 2.1.6**

Command:

```
biom convert -i Sargo_Curacao_FINAL/runs/MergedRun_NIOZ348_349_28-2-2024/asv/taxonomy_dada2/asvTable.txt -o Sargo_Curacao_FINAL/runs/MergedRun_NIOZ348_349_28-2-2024/asv/taxonomy_dada2/asvTable.biom --table-type 'OTU table' --table type "OTU table" --to-hdf5 --process-obs-metdata taxonomy
```

Output file:

- Biom format table: Sargo_Curacao_FINAL/runs/MergedRun_NIOZ348_349_28-2-2024/asv/taxonomy_dada2/asvTable.biom

Benchmark info:

s	max_rss	max_vms	max_uss	max_pss	io_in	io_out	mean_load
6.17	290.57	5623.60	288.44	288.46	194.17	15.51	0.00

Summarize Taxa

Summarize information of the representation of taxonomic groups within each sample.

Tool: [QIIME] - summarize_taxa.py

Version: **summarize_taxa.py 1.9.1**

Command:

```
summarize_taxa.py -i Sargo_Curacao_FINAL/runs/MergedRun_NIOZ348_349_28-2-2024/asv/taxonomy_dada2/otuTable.biom --level 2,3,4,5,6,7 -o Sargo_Curacao_FINAL/runs/MergedRun_NIOZ348_349_28-2-2024/asv/taxonomy_dada2/summary/
```

Output file:

- Taxonomy summarized counts at different taxonomy levels: Sargo_Curacao_FINAL/runs/MergedRun_NIOZ348_349_28-2-2024/asv/taxonomy_dada2/summary/otuTable_L**N**.txt

Where **N** is the taxonomy level. Default configuration produces levels from 2 to 6.

Benchmark info:

s	max_rss	max_vms	max_uss	max_pss	io_in	io_out	mean_load
68.07	322.02	5680.35	319.70	319.72	16.03	0.86	0.00

Filter ASV table

Filter ASVs from an ASV table based on their observed counts or identifier.

Tool: [QIIME] - filter_otus_from_otu_table.py

Version: **filter_otus_from_otu_table.py 1.9.1**

Minimum observation counts: 2

Command:

```
filter_otus_from_otu_table.py -i Sargo_Curacao_FINAL/runs/MergedRun_NIOZ348_349_28-2-
2024/asv/taxonomy_dada2/asvTable.biom -o Sargo_Curacao_FINAL/runs/MergedRun_NIOZ348_349_28-2-
2024/asv/taxonomy_dada2/asvTable_noSingletons.biom -n 2
```

Output file:

- Biom table: Sargo_Curacao_FINAL/runs/MergedRun_NIOZ348_349_28-2-2024/asv/taxonomy_dada2/otuTable_noSingletons.biom

Benchmark info:

s	max_rss	max_vms	max_uss	max_pss	io_in	io_out	mean_load
7.47	332.14	5597.27	329.80	329.83	88.00	14.04	0.00

Convert Filtered ASV table

Convert the filtered OTU table from the BIOM table format to a human readable format

Tool: [BIOM]

Version: **biom, version 2.1.6**

Command:

```
biom convert -i Sargo_Curacao_FINAL/runs/MergedRun_NIOZ348_349_28-2-
2024/otu/taxonomy_dada2/asvTable_noSingletons.biom -o Sargo_Curacao_FINAL/runs/MergedRun_NIOZ348_349_28-2-
2024/asv/taxonomy_dada2/asvTable_noSingletons.txt --table-type 'OTU table' --header-key taxonomy --to-tsv
```

Output file:

- TSV format table: Sargo_Curacao_FINAL/runs/MergedRun_NIOZ348_349_28-2-2024/asv/taxonomy_dada2/asvTable_noSingletons.txt

Benchmark info:

s	max_rss	max_vms	max_uss	max_pss	io_in	io_out	mean_load
12.15	336.20	5052.23	334.03	334.05	15.23	29.04	0.00

Filter representative sequences

Remove sequences according to the filtered OTU biom table.

Tool: [QIIME] - filter_fasta.py

Version: **filter_fasta.py 1.9.1**

Command:

```
filter_fasta.py -f Sargo_Curacao_FINAL/samples/MergedRun_NIOZ348_349_28-2-2024/asv/representative_seq_set.fasta -o
Sargo_Curacao_FINAL/samples/MergedRun_NIOZ348_349_28-2-
2024/asv/taxonomy_dada2/representative_seq_set_noSingletons.fasta -b
Sargo_Curacao_FINAL/samples/MergedRun_NIOZ348_349_28-2-2024/asv/taxonomy_dada2/otuTable_noSingletons.biom
```

Output file:

- Filtered fasta file: Sargo_Curacao_FINAL/samples/MergedRun_NIOZ348_349_28-2-2024/asv/taxonomy_dada2/representative_seq_set_noSingletons.fasta

Align representative sequences

Align the sequences in a FASTA file to each other or to a template sequence alignment.

Tool: [QIIME] - align_seqs.py

Version: TBD

Method: [pynast]

Command:

```
align_seqs.py -m pynast -i Sargo_Curacao_FINAL/runs/MergedRun_NIOZ348_349_28-2-2024/asv/dada2/representative_seq_set_noSingletons.fasta -o Sargo_Curacao_FINAL/runs/MergedRun_NIOZ348_349_28-2-2024/asv/taxonomy_dada2/aligned/representative_seq_set_noSingletons_aligned.fasta
```

Output files:

- Aligned fasta file: Sargo_Curacao_FINAL/runs/MergedRun_NIOZ348_349_28-2-2024/asv/taxonomy_dada2/aligned/representative_seq_set_noSingletons_aligned.fasta

- Log file: Sargo_Curacao_FINAL/runs/MergedRun_NIOZ348_349_28-2-2024/asv/taxonomy_dada2/aligned/representative_seq_set_noSingletons_log.txt

Benchmark info:

s	max_rss	max_vms	max_uss	max_pss	io_in	io_out	mean_load
1849.92	746.45	5672.27	743.95	743.97	69.89	201.61	0.00

Filter alignment

Removes positions which are gaps in every sequence.

Tool: [QIIME] - filter_alignment.py

Version: **filter_alignment.py 1.9.1**

Command:

```
filter_alignment.py -i Sargo_Curacao_FINAL/runs/MergedRun_NIOZ348_349_28-2-2024/asv/taxonomy_dada2/aligned/representative_seq_set_noSingletons_aligned.fasta -o Sargo_Curacao_FINAL/runs/MergedRun_NIOZ348_349_28-2-2024/asv/taxonomy_dada2/aligned/filtered/
```

Output file:

- Aligned fasta file: Sargo_Curacao_FINAL/runs/MergedRun_NIOZ348_349_28-2-2024/asv/taxonomy_dada2/aligned/representative_seq_set_noSingletons_aligned_pfiltered.fasta

Benchmark info:

s	max_rss	max_vms	max_uss	max_pss	io_in	io_out	mean_load
339.73	708.99	5651.32	706.66	706.68	443.58	0.02	0.00

Make tree

Create phylogenetic tree (newick format).

Tool: [QIIME] - make_phylogeny.py

Version: **make_phylogeny.py 1.9.1**

Method: [fasttree]

Command:

```
make_phylogeny.py -i Sargo_Curacao_FINAL/runs/MergedRun_NIOZ348_349_28-2-2024/asv/taxonomy_dada2/aligned/representative_seq_set_noSingletons_aligned.fasta -o representative_seq_set_noSingletons_aligned_pfiltered.tre -t fasttree
```

Output file:

- Taxonomy tree: Sargo_Curacao_FINAL/runs/MergedRun_NIOZ348_349_28-2-2024/asv/taxonomy_dada2/aligned/representative_seq_set_noSingletons_aligned.tre

Benchmark info:

s	max_rss	max_vms	max_uss	max_pss	io_in	io_out	mean_load
1704.72	759.39	5734.75	755.14	755.19	34.85	37.97	0.00

Krona report

Krona allows hierarchical data to be explored with zooming, multi-layered pie charts.

Tool: [Krona]

These charts were created using the ASV table **without** singletons

The report was executed for all the samples.

Each sample is represented on a separated chart (same html report).

You can see the report at the following link:

- Krona report: kreport

Or access the html file at:

- Krona html file: Sargo_Curacao_FINAL/runs/MergedRun_NIOZ348_349_28-2-2024/asv/taxonomy_dada2/krona_report.html

Benchmark info:

s	max_rss	max_vms	max_uss	max_pss	io_in	io_out	mean_load
19.66	32.25	1018.99	25.34	25.78	39.84	21.00	0.00

Final counts

Following the read counts:

File description	Location	#	(%)
Demultiplexed reads	Sargo_Curacao_FINAL/runs/MergedRun_NIOZ348_349_28-2-2024/<SAMPLE>_data/demultiplexed/*.fastq.gz	19685917	100%
QA filtered & trimmed reads	Sargo_Curacao_FINAL/runs/MergedRun_NIOZ348_349_28-2-2024/<LIBRARY>_data/demultiplexed/filtered/*.fastq.gz	17867189	90.76%
Denoised FW reads	*No intermediate file generated*	17374619	88.26%
Denoised RV reads	*NO intermediate file generated*	17293965	87.85%
Merged and full denoised reads	*No intermediate file generated*	13161189	66.86%
Length filtered	*No intermediate file generated*	13161189	66.86%
Chimera removed	*No intermediate file generated*	11048186	56.12%
ASV table	Sargo_Curacao_FINAL/runs/MergedRun_NIOZ348_349_28-2-2024/asv/asvTable.txt	65154	100%
Taxonomy assignation	Sargo_Curacao_FINAL/runs/MergedRun_NIOZ348_349_28-2-2024/asv/taxonomy_dada2/representative_seq_set_tax_assignments.txt	65120	99.95%
ASV table (no singletons: a > 2)	Sargo_Curacao_FINAL/runs/MergedRun_NIOZ348_349_28-2-2024/asv/taxonomy_dada2/asvTable_noSingletons.txt	61985	95.14%
Assigned no singletons	Sargo_Curacao_FINAL/runs/MergedRun_NIOZ348_349_28-2-2024/otu/taxonomy_vsearch/asvTable_noSingletons.txt	61951	99.95%

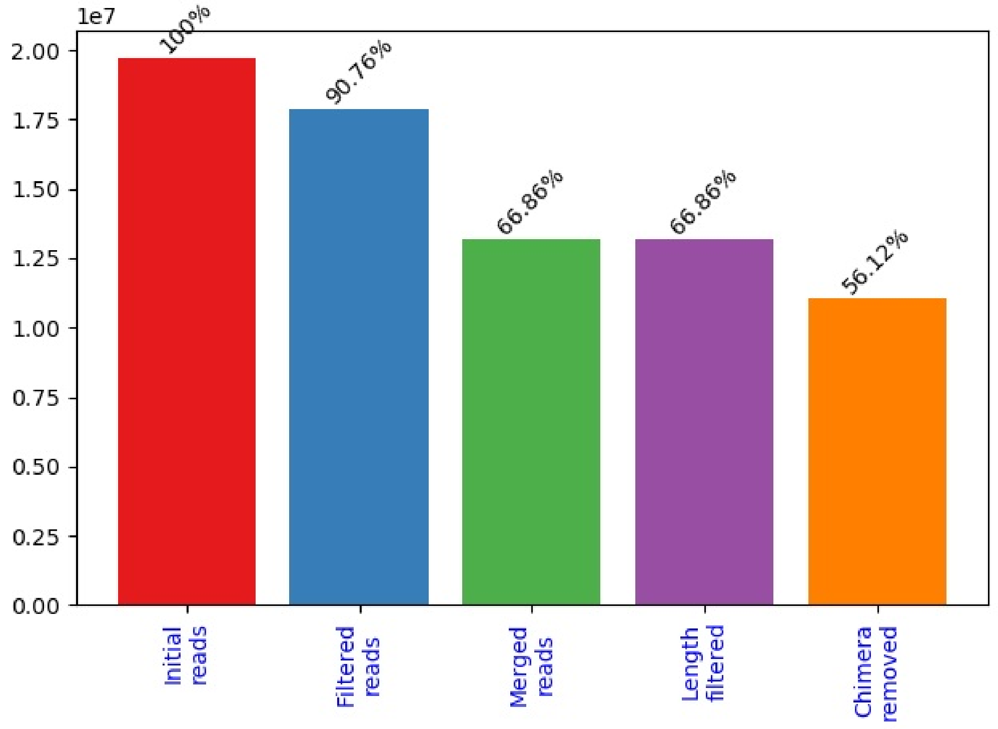

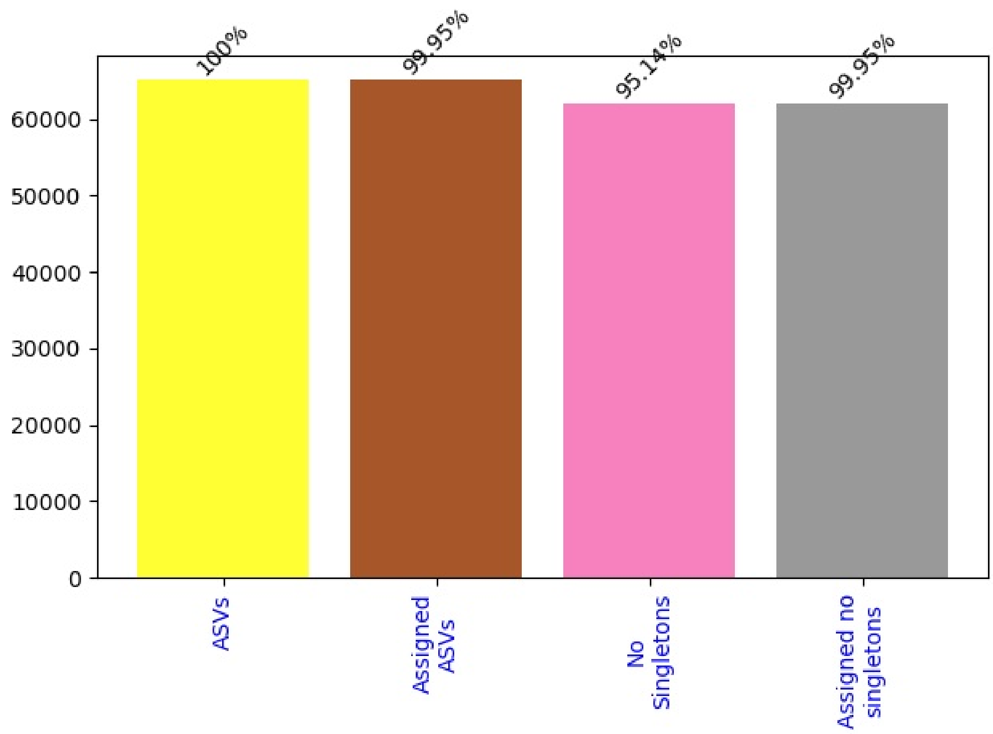

Note:

- Assigned ASVs percentage is the amount of successfully assigned ASVs.

- No singletons percentage is the percentage of no singletons ASVs in reference to the complete ASV table.

- Assigned No singletons is the amount of successfully no singletons assigned ASVs.

References

[QIIME] (1, 2, 3, 4, 5, 6) QIIME. Caporaso JG, Kuczynski J, Stombaugh J, Bittinger K, Bushman FD, Costello EK, Fierer N, Gonzalez Pena A, Goodrich JK, Gordon JI, Huttley GA, Kelley ST, Knights D, Koenig JE, Ley RE, Lozupone CA, McDonald D, Muegge BD, Pirrung M, Reeder J, Sevinsky JR, Turnbaugh PJ, Walters WA, Widmann J, Yatsunenko T, Zaneveld J, Knight R. 2010. QIIME allows analysis of high-throughput community sequencing data. Nature Methods 7(5): 335-336.

[Cutadapt] Cutadapt v1.15 .Marcel Martin. Cutadapt removes adapter sequences from high-throughput sequencing reads. EMBnet.Journal, 17(1):10-12, May 2011. http://dx.doi.org/10.14806/ej.17.1.200

[vsearch] Rognes T, Flouri T, Nichols B, Quince C, Mahé F. (2016) VSEARCH: a versatile open source tool for metagenomics. PeerJ 4:e2584. doi: 10.7717/peerj.2584

[Krona] Ondov BD, Bergman NH, and Phillippy AM. Interactive metagenomic visualization in a Web browser. BMC Bioinformatics. 2011 Sep 30; 12(1):385.

[BIOM] (1, 2) The Biological Observation Matrix (BIOM) format or: how I learned to stop worrying and love the ome-ome. Daniel McDonald, Jose C. Clemente, Justin Kuczynski, Jai Ram Rideout, Jesse Stombaugh, Doug Wendel, Andreas Wilke, Susan Huse, John Hufnagle, Folker Meyer, Rob Knight, and J. Gregory Caporaso.GigaScience 2012, 1:7. doi:10.1186/2047-217X-1-7

[RDP] Wang, Q, G. M. Garrity, J. M. Tiedje, and J. R. Cole. 2007. Naive Bayesian Classifier for Rapid Assignment of rRNA Sequences into the New Bacterial Taxonomy. Appl Environ Microbiol. 73(16):5261-7.

[dada2] Callahan BJ, McMurdie PJ, Rosen MJ, Han AW, Johnson AJA, Holmes SP (2016). DADA2: High-resolution sample inference from Illumina amplicon data. Nature Methods, 13, 581-583. doi: 10.1038/nmeth.3869.

[pynast] Caporaso JG, Bittinger K, Bushman FD, DeSantis TZ, Andersen GL, Knight R. 2010. PyNAST: a flexible tool for aligning sequences to a template alignment. Bioinformatics 26:266-267.

[fasttree] Price MN, Dehal PS, Arkin AP. 2010. FastTree 2-Approximately Maximum-Likelihood Trees for Large Alignments. Plos One 5(3).

Unpaired workflow for analysis of longer 18S rRNA fragment

snakemake report

Amplicon Analysis Report for Library: NIOZ348

Cascabel is designed to run amplicon sequence analysis across single or multiple read libraries.

The objective of this pipeline is to create different output files which allow the user to explore data in a simple and meaningful way, as well as facilitate downstream analysis, based on the generated output files.

Another aim of **Cascabel** is also to encourage the documentation process, by creating this report in order to assure data analysis reproducibility.

Cascabel version: version 6.0.2

Note: Library demultiplexing has been carried out, if you have controls among your samples, please be aware that Cascabel won't perform any special operation with them. They are treated as any other sample within this workflow. Please make sure to analyze your controls with other tools, and correct your sample counts for potential contamination.

Following you can see all the steps that were taken in order to get the final results of the pipeline.

Raw Data

The raw data for this library can be found at:

- FW raw reads: SARGO_Curacao_18S_unpaired/samples/NIOZ348/rawdata/fw.fastq

- RV raw reads: SARGO_Curacao_18S_unpaired/samples/NIOZ348/rawdata/rv.fastq

Number of total reads: 63154513

Quality Control

Evaluate quality on raw reads.

Tool: [FastQC]

Version: **FastQC v0.11.9**

Command:

```
fastqc SARGO_Curacao_18S_unpaired/samples/NIOZ348/rawdata/fw.fastq
SARGO_Curacao_18S_unpaired/samples/NIOZ348/rawdata/rv.fastq --extract -o
SARGO_Curacao_18S_unpaired/samples/NIOZ348/qc/
```

You can follow the links below, in order to see the complete FastQC report:

- FastQC for sample NIOZ348_1: FQ1

- FastQC for sample NIOZ348_2: FQ2

Benchmark info:

s	max_rss	max_vms	max_uss	max_pss	io_in	io_out	mean_load
1153.37	1644.10	6268.07	1642.28	1642.29	14413.51	0.74	0.00

```
pear -f SARGO_Curacao_18S_unpaired/samples/NIOZ348/rawdata/fw.fastq -r
SARGO_Curacao_18S_unpaired/samples/NIOZ348/rawdata/rv.fastq -t 100 -v 10 -j 20 -p 0.05 -o
SARGO_Curacao_18S_unpaired/samples/NIOZ348/peared/seqs >
SARGO_Curacao_18S_unpaired/samples/NIOZ348/peared/seqs.assembled.fastq
```

Output files:

- Merged reads: SARGO_Curacao_18S_unpaired/samples/NIOZ348/peared/seqs.assembled.fastq

- Log file: SARGO_Curacao_18S_unpaired/samples/NIOZ348/peared/pear.log

Number of peared reads: 4027851=6.38%

Benchmark info:

s	max_rss	max_vms	max_uss	max_pss	io_in	io_out	mean_load
5434.03	189.80	1756.34	187.87	187.89	0.69	55865.11	0.00

Peared FastQC Analysis

Check the quality of the reads after assembly.

Tool: [FastQC]

Version: **FastQC v0.11.9**

Command:

```
fastqc SARGO_Curacao_18S_unpaired/samples/NIOZ348/peared/seqs.assembled.fastq --extract -o
SARGO_Curacao_18S_unpaired/samples/NIOZ348/peared/qc
```

Output files:

- FastQC report: SARGO_Curacao_18S_unpaired/samples/NIOZ348/peared/qc/seqs.assembled_fastqc.html FQ_Report

Benchmark data not found: SARGO_Curacao_18S_unpaired/samples/NIOZ348/peared/qc/fq.benchmark

Extract barcodes

Extract the barcodes used to identify individual samples.

Tool: [QIIME] - extract_barcodes.py

Version: **extract_barcodes.py 1.9.1**

Command:

```
extract_barcodes.py -f SARGO_Curacao_18S_unpaired/samples/NIOZ348/peared/seqs.assembled.fastq -c
barcode_paired_stitched --bc1_len 12 --bc2_len 12 -o SARGO_Curacao_18S_unpaired/samples/NIOZ348/barcodes_unpaired/
```

Output files:

- Fastq file with barcodes: SARGO_Curacao_18S_unpaired/samples/NIOZ348/barcodes_unpaired/barcodes.fastq

- Fastq file with the reads: SARGO_Curacao_18S_unpaired/samples/NIOZ348/barcodes_unpaired/reads.fastq

Benchmark info:

s	max_rss	max_vms	max_uss	max_pss	io_in	io_out	mean_load
161.57	119.03	5744.97	116.70	116.72	4229.66	4359.23	0.00

Correct Barcodes

Try to correct the barcode from unassigned reads. and place reads in correct orientetion.

Maximum number of mismatches **1**.

Tool: Cascabel Java program

Command:

```
java -jar Scripts/BarcodeCorrector.jar -b SARGO_Curacao_18S_unpaired/metadata/sampleList_mergedBarcodes_NIOZ348.txt -fb
SARGO_Curacao_18S_unpaired/samples/NIOZ348/barcodes_unpaired/barcodes.fastq -fr
SARGO_Curacao_18S_unpaired/samples/NIOZ348/barcodes_unpaired/reads.fastq -m 1 -o
SARGO_Curacao_18S_unpaired/samples/NIOZ348/barcodes_unpaired/barcodes.fastq_corrected -or
SARGO_Curacao_18S_unpaired/samples/NIOZ348/barcodes_unpaired/reads.fastq_corrected -rc -x
SARGO_Curacao_18S_unpaired/samples/NIOZ348/barcodes_unpaired/sample_matrix.txt >
SARGO_Curacao_18S_unpaired/samples/NIOZ348/barcodes_unpaired/demux.log
```

Output files:

- Barcode corrected file: SARGO_Curacao_18S_unpaired/samples/NIOZ348/barcodes_unpaired/barcodes.fastq_corrected

- Reads corrected file: SARGO_Curacao_18S_unpaired/samples/NIOZ348/barcodes_unpaired/reads.fastq_corrected

- Error correction summary: SARGO_Curacao_18S_unpaired/samples/NIOZ348/barcodes_unpaired/demux.log

Benchmark info:

s	max_rss	max_vms	max_uss	max_pss	io_in	io_out	mean_load
320.01	1439.20	38309.14	1437.70	1437.72	4967.52	4962.62	0.00

Demultiplexing

For library splitting, also known as demultiplexing, Cascabel performs several steps to assign fragments in the original as well as reverse orientation to the correct sample.

Split samples from Fastq file

Tool: [QIIME] - split_libraries_fastq.py

version: **split_libraries_fastq.py 1.9.1**

Command:

```
split_libraries_fastq.py -m SARGO_Curacao_18S_unpaired/metadata/sampleList_mergedBarcodes_NIOZ348.txt -i
SARGO_Curacao_18S_unpaired/samples/NIOZ348/barcodes_unpaired/reads.fastq -o
SARGO_Curacao_18S_unpaired/samples/NIOZ348/splitLibs_unpaired -b
SARGO_Curacao_18S_unpaired/samples/NIOZ348/barcodes_unpaired/barcodes.fastq_corrected -q 19 -r 5 --
retain_unassigned_reads --phred_offset 33 --barcode_type 24
```

Benchmark info:

s	max_rss	max_vms	max_uss	max_pss	io_in	io_out	mean_load
345.56	213.70	5611.02	211.40	211.43	4967.52	1900.47	0.00

Retain assigned reads

Command:

```
cat SARGO_Curacao_18S_unpaired/samples/NIOZ348/splitLibs_unpaired/seqs.fna | grep -P -A1 "(?!>Unass)^>" | sed '/^--$/d' >
SARGO_Curacao_18S_unpaired/samples/NIOZ348/splitLibs_unpaired/seqs.no_unassigneds.fna
```

Create file with only unassigned reads

Command:

```
cat SARGO_Curacao_18S_unpaired/samples/NIOZ348/splitLibs_unpaired/seqs.fna | grep "^>Unassigned" | sed
's/>Unassigned_[0-9]* /@/g' | sed 's/ .*//' | grep -F -w -A3 -f -
SARGO_Curacao_18S_unpaired/samples/NIOZ348/peared/seqs.assembled.fastq | sed '/^--$/d'
>SARGO_Curacao_18S_unpaired/samples/NIOZ348/splitLibs_unpaired/unassigned.fastq
```

Output files:

- Text histogram with the length of the fw reads: SARGO_Curacao_18S_unpaired/samples/NIOZ348/splitLibs_unpaired/histograms.txt

- Split library log: SARGO_Curacao_18S_unpaired/samples/NIOZ348/splitLibs_unpaired/split_library_log.txt

Number of reads assigned on FW: 262966 = 6.53% of the peared reads

Number of reads assigned on RVC: 448276 = 11.13% of the peared reads

Generate single sample fastq files

Create single fastq files per samples (based on the raw data without applying any filtering).

Tool: Cascabel Java program

Command:

```
java -cp Scripts DemultiplexQiime --txt -a rv -b 1 -d SARGO_Curacao_18S_unpaired/runs/SARGO_18S_4-4-2024_run1/NIOZ348_data/seqs.assigned.ori.txt -o SARGO_Curacao_18S_unpaired/samples/NIOZ348/demultiplexed/unpaired/ -r1 SARGO_Curacao_18S_unpaired/samples/NIOZ348/rawdata/fw.fastq.gz -r2 SARGO_Curacao_18S_unpaired/samples/NIOZ348/rawdata/fw.fastq.gz
```

Barcodes removed: 12 first bases

The demultiplexed files are located at:

- Demultiplexed directory: SARGO_Curacao_18S_unpaired/samples/NIOZ348/demultiplexed/unpaired/

- Summary file: SARGO_Curacao_18S_unpaired/samples/NIOZ348/demultiplexed/unpaired/summary.pcr.txt

Benchmark info:

s	max_rss	max_vms	max_uss	max_pss	io_in	io_out	mean_load
8205.63	249.57	38674.21	246.62	246.66	20435.12	54.59	0.00

Remove primers:

Following, primers were removed from the fastq files

Remove primers from fastq files.

Primers removed: **FW** GTGYCAGCMGCCGCGGTAA **RV** CCGYCAATTYMTTTRAGTTT

Tool: [Cutadapt]

Version: **cutadapt v1.16**

Command:

```
cutadapt -g GTGYCAGCMGCCGCGGTAA -G CCGYCAATTYMTTTRAGTTT --discard-untrimmed --match-read-wildcards -O 15 -m 100 -o SARGO_Curacao_18S_unpaired/samples/NIOZ348/demultiplexed/unpaired/primer_removed/SAMPLE_1.fastq.gz -p SARGO_Curacao_18S_unpaired/samples/NIOZ348/demultiplexed/unpaired/primer_removed/SAMPLE_2.fastq.gz SARGO_Curacao_18S_unpaired/samples/NIOZ348/demultiplexed/unpaired/SAMPLE_1.fq.gz SARGO_Curacao_18S_unpaired/samples/NIOZ348/demultiplexed/unpaired/SAMPLE_2.fq.gz >> SARGO_Curacao_18S_unpaired/samples/NIOZ348/demultiplexed/unpaired/primer_removed/NIOZ348.cutadapt.log
```

The above command ran once for each single sample fastq file(s) using the mentioned primers

- Reads without primers: SARGO_Curacao_18S_unpaired/samples/NIOZ348/demultiplexed/unpaired/primer_removed

- Discarded reads (no primer): SARGO_Curacao_18S_unpaired/samples/NIOZ348/demultiplexed/unpaired/reads_discarded_primer

- Primer removal results by sample: primers_removal

Benchmark info:

s	max_rss	max_vms	max_uss	max_pss	io_in	io_out	mean_load
32.59	36.41	603.32	24.19	25.53	52.55	28.07	0.00

Sample distribution

Sample	Seqs	prc.
NIOZ348.926R.9135.9138	2545	0.36
NIOZ348.926R.9137.9140	274	0.04
NIOZ348.926R.9139.9142	14710	2.07
NIOZ348.926R.9141.9144	11149	1.57
NIOZ348.926R.9143.9146	16122	2.27
NIOZ348.926R.9145.9148	81245	11.42
NIOZ348.926R.9147.9150	15045	2.12

Sample	Seqs	prc.
NIOZ348.926R.9149.9152	7446	1.05
NIOZ348.926R.9151.9154	13611	1.91
NIOZ348.926R.9153.9156	6440	0.91
NIOZ348.926R.9155.9158	186363	26.20
NIOZ348.926R.9157.9160	17247	2.42
NIOZ348.926R.9159.9162	16921	2.38
NIOZ348.926R.9161.9164	35617	5.01

Sample	Seqs	prc.
NIOZ348.926R.9163.9166	19813	2.79
NIOZ348.926R.9165.9168	19681	2.77
NIOZ348.926R.9167.9170	19421	2.73
NIOZ348.926R.9169.9172	21759	3.06
NIOZ348.926R.9171.9174	14556	2.05
NIOZ348.926R.9173.9176	11171	1.57
NIOZ348.926R.9175.9178	94932	13.35

Sample	Seqs	prc.
NIOZ348.926R.9177.9180	13136	1.85
NIOZ348.926R.9179.9182	22396	3.15
NIOZ348.926R.9181.9184	12896	1.81
NIOZ348.926R.9183.9186	18619	2.62
NIOZ348.926R.9185.9188	1630	0.23
NIOZ348.926R.9187.9190	2	0.00

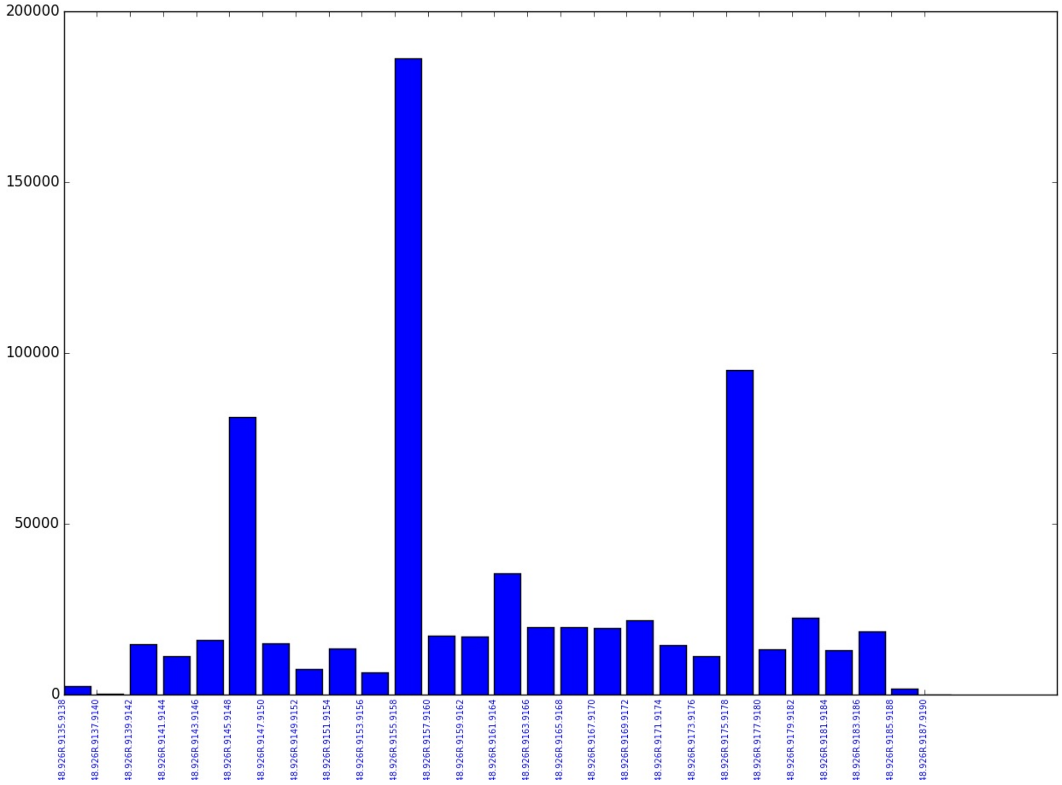

The previous chart shows the number of clean reads per sample. The bars are sorted from left to right, according to the metadata input file.

To see more details about the number of reads per sample in this library, please refer to the file: SARGO_Curacao_18S_unpaired/runs/SARGO_18S_4-4-2024_run1/NIOZ348_data/seqs_fw_rev_filtered.dist.txt

Final counts

Following you can see the final read counts:

File description	Location	Number of reads	Prc(%) vs raw
Raw reads	SARGO_Curacao_18S_unpaired/samples/NIOZ348/rawdata/*.fq	63154513.0	100.00%
Assembled reads	SARGO_Curacao_18S_unpaired/samples/NIOZ348/peared/seqs.assembled.fastq	4027851.0	6.38%
Demultiplexed reads	SARGO_Curacao_18S_unpaired/runs/SARGO_18S_4-4-2024_run1/NIOZ348_data/seqs_fw_rev_accepted.fna	711242	1.13%
Primer removed	SARGO_Curacao_18S_unpaired/samples/NIOZ348/demultiplexed/unpaired/primer_removed/	694747.0	1.10%

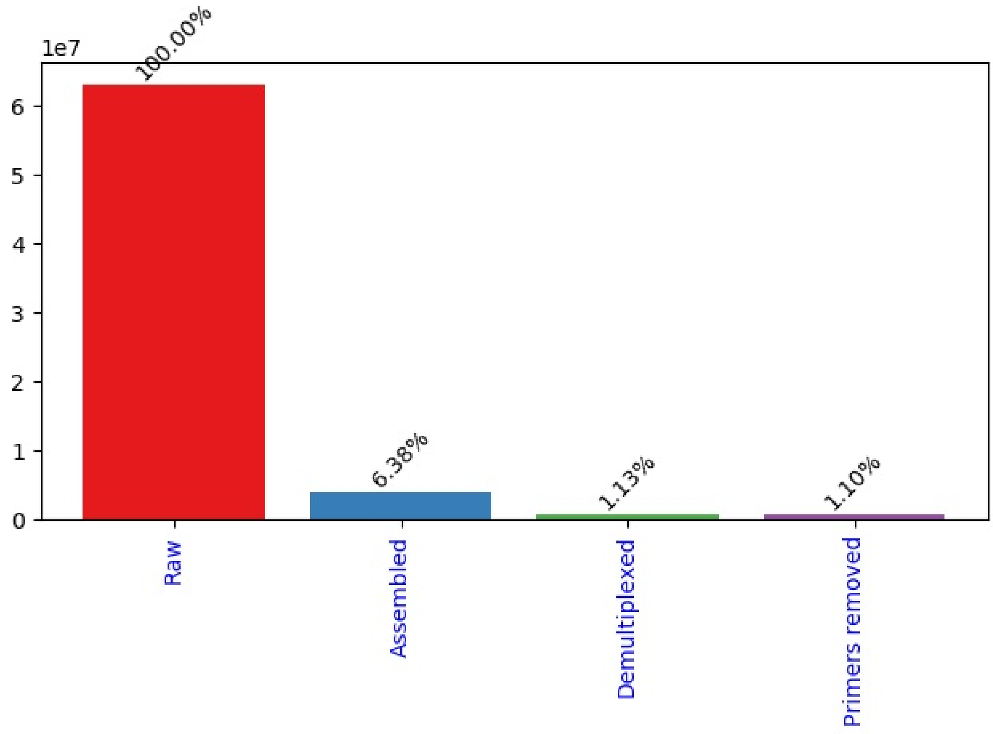

ASV report

Cascabel report on downstream analyses in combination with multiple libraries (if supplied), can be found at the following link: asv_report (SARGO_Curacao_18S_unpaired/runs/SARGO_18S_4-4-2024_run1/asv_report_dada2.html)

References

[FastQC] (1, 2) FastQC v0.11.3. Andrews S. (2010). FastQC: a quality control tool for high throughput sequence data

[PEAR] PEAR: a fast and accurate Illumina Paired-End reAd mergeR. Zhang et al (2014) Bioinformatics 30(5): 614-620 | doi:10.1093/bioinformatics/btt593

[QIIME] (1, 2) QIIME. Caporaso JG, Kuczynski J, Stombaugh J, Bittinger K, Bushman FD, Costello EK, Fierer N, Gonzalez Pena A, Goodrich JK, Gordon JI, Huttley GA, Kelley ST, Knights D, Koenig JE, Ley RE, Lozupone CA, McDonald D, Muegge BD, Pirrung M, Reeder J, Sevinsky JR, Turnbaugh PJ, Walters WA, Widmann J, Yatsunenko T, Zaneveld J, Knight R. 2010. QIIME allows analysis of high-throughput community sequencing data. Nature Methods 7(5): 335-336.

[Cutadapt] Cutadapt v1.15 .Marcel Martin. Cutadapt removes adapter sequences from high-throughput sequencing reads. EMBnet.Journal, 17(1):10-12, May 2011. http://dx.doi.org/10.14806/ej.17.1.200

[Vsearch] Rognes T, Flouri T, Nichols B, Quince C, Mahé F. (2016) VSEARCH: a versatile open source tool for metagenomics. PeerJ 4:e2584. doi: 10.7717/peerj.2584

Amplicon Analysis Report for Library: NIOZ349

Cascabel is designed to run amplicon sequence analysis across single or multiple read libraries.

The objective of this pipeline is to create different output files which allow the user to explore data in a simple and meaningful way, as well as facilitate downstream analysis, based on the generated output files.

Another aim of **Cascabel** is also to encourage the documentation process, by creating this report in order to assure data analysis reproducibility.

Cascabel version: version 6.0.2

Note: Library demultiplexing has been carried out, if you have controls among your samples, please be aware that Cascabel won't perform any special operation with them. They are treated as any other sample within this workflow. Please make sure to analyze your controls with other tools, and correct your sample counts for potential contamination.

Following you can see all the steps that were taken in order to get the final results of the pipeline.

Raw Data

The raw data for this library can be found at:

- FW raw reads: SARGO_Curacao_18S_unpaired/samples/NIOZ349/rawdata/fw.fastq

- RV raw reads: SARGO_Curacao_18S_unpaired/samples/NIOZ349/rawdata/rv.fastq

Number of total reads: 63438353

Quality Control

Evaluate quality on raw reads.

Tool: [FastQC]

Version: **FastQC v0.11.9**

Command:

```
fastqc SARGO_Curacao_18S_unpaired/samples/NIOZ349/rawdata/fw.fastq
SARGO_Curacao_18S_unpaired/samples/NIOZ349/rawdata/rv.fastq --extract -o
SARGO_Curacao_18S_unpaired/samples/NIOZ349/qc/
```

You can follow the links below, in order to see the complete FastQC report:

- FastQC for sample NIOZ349_1: FQ1

- FastQC for sample NIOZ349_2: FQ2

Benchmark info:

s	max_rss	max_vms	max_uss	max_pss	io_in	io_out	mean_load
1173.61	1769.44	6221.86	1767.56	1767.58	14177.31	4.80	0.00

```
pear -f SARGO_Curacao_18S_unpaired/samples/NIOZ349/rawdata/fw.fastq -r
SARGO_Curacao_18S_unpaired/samples/NIOZ349/rawdata/rv.fastq -t 100 -v 10 -j 20 -p 0.05 -o
SARGO_Curacao_18S_unpaired/samples/NIOZ349/peared/seqs >
SARGO_Curacao_18S_unpaired/samples/NIOZ349/peared/seqs.assembled.fastq
```

Output files:

- Merged reads: SARGO_Curacao_18S_unpaired/samples/NIOZ349/peared/seqs.assembled.fastq

- Log file: SARGO_Curacao_18S_unpaired/samples/NIOZ349/peared/pear.log

Number of peared reads: 5218901=8.23%

Benchmark info:

s	max_rss	max_vms	max_uss	max_pss	io_in	io_out	mean_load
5475.42	189.79	1756.34	187.86	187.88	0.00	56982.64	0.00

Peared FastQC Analysis

Check the quality of the reads after assembly.

Tool: [FastQC]

Version: **FastQC v0.11.9**

Command:

```
fastqc SARGO_Curacao_18S_unpaired/samples/NIOZ349/peared/seqs.assembled.fastq --extract -o
SARGO_Curacao_18S_unpaired/samples/NIOZ349/peared/qc
```

Output files:

- FastQC report: SARGO_Curacao_18S_unpaired/samples/NIOZ349/peared/qc/seqs.assembled_fastqc.html FQ_Report

Benchmark data not found: SARGO_Curacao_18S_unpaired/samples/NIOZ349/peared/qc/fq.benchmark

Extract barcodes

Extract the barcodes used to identify individual samples.

Tool: [QIIME] - extract_barcodes.py

Version: **extract_barcodes.py 1.9.1**

Command:

```
extract_barcodes.py -f SARGO_Curacao_18S_unpaired/samples/NIOZ349/peared/seqs.assembled.fastq -c
barcode_paired_stitched --bc1_len 12 --bc2_len 12 -o SARGO_Curacao_18S_unpaired/samples/NIOZ349/barcodes_unpaired/
```

Output files:

- Fastq file with barcodes: SARGO_Curacao_18S_unpaired/samples/NIOZ349/barcodes_unpaired/barcodes.fastq

- Fastq file with the reads: SARGO_Curacao_18S_unpaired/samples/NIOZ349/barcodes_unpaired/reads.fastq

Benchmark info:

s	max_rss	max_vms	max_uss	max_pss	io_in	io_out	mean_load
162.27	119.01	5576.13	116.68	116.71	5317.59	5460.77	0.00

Correct Barcodes

Try to correct the barcode from unassigned reads. and place reads in correct orientetion.

Maximum number of mismatches **1**.

Tool: Cascabel Java program

Command:

```
java -jar Scripts/BarcodeCorrector.jar -b SARGO_Curacao_18S_unpaired/metadata/sampleList_mergedBarcodes_NIOZ349.txt -fb
SARGO_Curacao_18S_unpaired/samples/NIOZ349/barcodes_unpaired/barcodes.fastq -fr
SARGO_Curacao_18S_unpaired/samples/NIOZ349/barcodes_unpaired/reads.fastq -m 1 -o
SARGO_Curacao_18S_unpaired/samples/NIOZ349/barcodes_unpaired/barcodes.fastq_corrected -or
SARGO_Curacao_18S_unpaired/samples/NIOZ349/barcodes_unpaired/reads.fastq_corrected -rc -x
SARGO_Curacao_18S_unpaired/samples/NIOZ349/barcodes_unpaired/sample_matrix.txt >
SARGO_Curacao_18S_unpaired/samples/NIOZ349/barcodes_unpaired/demux.log
```

Output files:

- Barcode corrected file: SARGO_Curacao_18S_unpaired/samples/NIOZ349/barcodes_unpaired/barcodes.fastq_corrected

- Reads corrected file: SARGO_Curacao_18S_unpaired/samples/NIOZ349/barcodes_unpaired/reads.fastq_corrected

- Error correction summary: SARGO_Curacao_18S_unpaired/samples/NIOZ349/barcodes_unpaired/demux.log

Benchmark info:

s	max_rss	max_vms	max_uss	max_pss	io_in	io_out	mean_load
797.65	1428.75	38243.44	1427.12	1427.13	6270.04	6253.73	0.00

Demultiplexing

For library splitting, also known as demultiplexing, Cascabel performs several steps to assign fragments in the original as well as reverse orientation to the correct sample.

Split samples from Fastq file

Tool: [QIIME] - split_libraries_fastq.py

version: **split_libraries_fastq.py 1.9.1**

Command:

```
split_libraries_fastq.py -m SARGO_Curacao_18S_unpaired/metadata/sampleList_mergedBarcodes_NIOZ349.txt -i
SARGO_Curacao_18S_unpaired/samples/NIOZ349/barcodes_unpaired/reads.fastq -o
SARGO_Curacao_18S_unpaired/samples/NIOZ349/splitLibs_unpaired -b
SARGO_Curacao_18S_unpaired/samples/NIOZ349/barcodes_unpaired/barcodes.fastq_corrected -q 19 -r 5 --
retain_unassigned_reads --phred_offset 33 --barcode_type 24
```

Benchmark info:

s	max_rss	max_vms	max_uss	max_pss	io_in	io_out	mean_load
442.25	253.92	5587.63	251.66	251.69	6428.70	2778.78	0.00

Retain assigned reads

Command:

```
cat SARGO_Curacao_18S_unpaired/samples/NIOZ349/splitLibs_unpaired/seqs.fna | grep -P -A1 "(?!>Unass)^>" | sed '/^--$/d' >
SARGO_Curacao_18S_unpaired/samples/NIOZ349/splitLibs_unpaired/seqs.no_unassigneds.fna
```

Create file with only unassigned reads

Command:

```
cat SARGO_Curacao_18S_unpaired/samples/NIOZ349/splitLibs_unpaired/seqs.fna | grep "^>Unassigned" | sed
's/>Unassigned_[0-9]* /@/g' | sed 's/ .*//' | grep -F -w -A3 -f -
SARGO_Curacao_18S_unpaired/samples/NIOZ349/peared/seqs.assembled.fastq | sed '/^--$/d'
>SARGO_Curacao_18S_unpaired/samples/NIOZ349/splitLibs_unpaired/unassigned.fastq
```

Output files:

- Text histogram with the length of the fw reads: SARGO_Curacao_18S_unpaired/samples/NIOZ349/splitLibs_unpaired/histograms.txt

- Split library log: SARGO_Curacao_18S_unpaired/samples/NIOZ349/splitLibs_unpaired/split_library_log.txt

Number of reads assigned on FW: 660069 = 12.65% of the peared reads

Number of reads assigned on RVC: 1113845 = 21.34% of the peared reads

Generate single sample fastq files

Create single fastq files per samples (based on the raw data without applying any filtering).

Tool: Cascabel Java program

Command:

```
java -cp Scripts DemultiplexQiime --txt -a rv -b 1 -d SARGO_Curacao_18S_unpaired/runs/SARGO_18S_4-4-2024_run1/NIOZ349_data/seqs.assigned.ori.txt -o SARGO_Curacao_18S_unpaired/samples/NIOZ349/demultiplexed/unpaired/ -r1 SARGO_Curacao_18S_unpaired/samples/NIOZ349/rawdata/fw.fastq.gz -r2 SARGO_Curacao_18S_unpaired/samples/NIOZ349/rawdata/fw.fastq.gz
```

Barcodes removed: 12 first bases

The demultiplexed files are located at:

- Demultiplexed directory: SARGO_Curacao_18S_unpaired/samples/NIOZ349/demultiplexed/unpaired/

- Summary file: SARGO_Curacao_18S_unpaired/samples/NIOZ349/demultiplexed/unpaired/summary.pcr.txt

Benchmark info:

s	max_rss	max_vms	max_uss	max_pss	io_in	io_out	mean_load
23385.38	604.75	38796.55	600.36	600.40	186.92	135.85	0.00

Remove primers:

Following, primers were removed from the fastq files

Remove primers from fastq files.

Primers removed: **FW** GTGYCAGCMGCCGCGGTAA **RV** CCGYCAATTYMTTTRAGTTT

Tool: [Cutadapt]

Version: **cutadapt v1.16**

Command:

```
cutadapt -g GTGYCAGCMGCCGCGGTAA -G CCGYCAATTYMTTTRAGTTT --discard-untrimmed --match-read-wildcards -O 15 -m 100 -o SARGO_Curacao_18S_unpaired/samples/NIOZ349/demultiplexed/unpaired/primer_removed/SAMPLE_1.fastq.gz -p SARGO_Curacao_18S_unpaired/samples/NIOZ349/demultiplexed/unpaired/primer_removed/SAMPLE_2.fastq.gz SARGO_Curacao_18S_unpaired/samples/NIOZ349/demultiplexed/unpaired/SAMPLE_1.fq.gz SARGO_Curacao_18S_unpaired/samples/NIOZ349/demultiplexed/unpaired/SAMPLE_2.fq.gz >> SARGO_Curacao_18S_unpaired/samples/NIOZ349/demultiplexed/unpaired/primer_removed/NIOZ349.cutadapt.log
```

The above command ran once for each single sample fastq file(s) using the mentioned primers

- Reads without primers: SARGO_Curacao_18S_unpaired/samples/NIOZ349/demultiplexed/unpaired/primer_removed

- Discarded reads (no primer): SARGO_Curacao_18S_unpaired/samples/NIOZ349/demultiplexed/unpaired/reads_discarded_primer

- Primer removal results by sample: primers_removal

Benchmark info:

s	max_rss	max_vms	max_uss	max_pss	io_in	io_out	mean_load
80.04	35.96	603.07	24.10	25.48	226.25	129.89	0.00

Sample distribution

Sample	Seqs	prc.
NIOZ349.926R.0001.0034	3498	0.20
NIOZ349.926R.0003.0036	13220	0.75
NIOZ349.926R.0005.0038	44247	2.49
NIOZ349.926R.0007.0040	10530	0.59
NIOZ349.926R.0009.0042	11251	0.63
NIOZ349.926R.0011.0044	23296	1.31
NIOZ349.926R.0013.0046	19889	1.12
NIOZ349.926R.0015.0048	19673	1.11
NIOZ349.926R.0017.0050	25097	1.41
NIOZ349.926R.0019.0052	14549	0.82
NIOZ349.926R.0021.0054	33976	1.92
NIOZ349.926R.0023.0056	37848	2.13
NIOZ349.926R.0025.0058	20789	1.17
NIOZ349.926R.0027.0060	26087	1.47
NIOZ349.926R.0029.0062	19943	1.12
NIOZ349.926R.0031.0064	20320	1.15
NIOZ349.926R.0033.0066	13414	0.76
NIOZ349.926R.0035.0068	18628	1.05
NIOZ349.926R.0037.0070	16542	0.93

Sample	Seqs	prc.
NIOZ349.926R.0039.0072	28927	1.63
NIOZ349.926R.0041.0074	36580	2.06
NIOZ349.926R.0043.0076	14481	0.82
NIOZ349.926R.0045.0078	28686	1.62
NIOZ349.926R.0047.0080	23124	1.30
NIOZ349.926R.0049.0082	33815	1.91
NIOZ349.926R.0051.0084	23073	1.30
NIOZ349.926R.0053.0086	17821	1.00
NIOZ349.926R.0055.0088	21777	1.23
NIOZ349.926R.0057.0090	16140	0.91
NIOZ349.926R.0059.0092	27437	1.55
NIOZ349.926R.0061.0094	10926	0.62
NIOZ349.926R.0063.0096	6	0.00
NIOZ349.926R.0065.0098	34720	1.96
NIOZ349.926R.0067.0100	38972	2.20
NIOZ349.926R.0069.0102	21014	1.18
NIOZ349.926R.0071.0104	39193	2.21
NIOZ349.926R.0073.0106	22923	1.29
NIOZ349.926R.0075.0108	15294	0.86

Sample	Seqs	prc.
NIOZ349.926R.0077.0110	27726	1.56
NIOZ349.926R.0079.0112	29037	1.64
NIOZ349.926R.0081.0114	23555	1.33
NIOZ349.926R.0083.0116	14193	0.80
NIOZ349.926R.0085.0118	28520	1.61
NIOZ349.926R.0087.0120	19384	1.09
NIOZ349.926R.0089.0122	33790	1.90
NIOZ349.926R.0091.0124	28049	1.58
NIOZ349.926R.0093.0126	18660	1.05
NIOZ349.926R.0095.0128	22994	1.30
NIOZ349.926R.0097.0130	15226	0.86
NIOZ349.926R.0099.0132	22378	1.26
NIOZ349.926R.0101.0134	26109	1.47
NIOZ349.926R.0103.0136	31154	1.76
NIOZ349.926R.0105.0138	27936	1.57
NIOZ349.926R.0107.0140	16945	0.96
NIOZ349.926R.0109.0142	23062	1.30
NIOZ349.926R.0111.0144	27571	1.55
NIOZ349.926R.0113.0146	29924	1.69

Sample	Seqs	prc.
NIOZ349.926R.0115.0148	38958	2.20
NIOZ349.926R.0117.0150	25185	1.42
NIOZ349.926R.0119.0152	31894	1.80
NIOZ349.926R.0121.0154	27264	1.54
NIOZ349.926R.0123.0156	23803	1.34
NIOZ349.926R.0125.0158	13578	0.77
NIOZ349.926R.0127.0160	1795	0.10
NIOZ349.926R.0129.0162	10272	0.58
NIOZ349.926R.0131.0164	25223	1.42
NIOZ349.926R.0133.0166	28123	1.59
NIOZ349.926R.0135.0168	33755	1.90
NIOZ349.926R.0137.0170	25319	1.43
NIOZ349.926R.0139.0172	12612	0.71
NIOZ349.926R.0141.0174	38254	2.16
NIOZ349.926R.0143.0176	30473	1.72
NIOZ349.926R.0145.0178	28487	1.61
NIOZ349.926R.0147.0180	18827	1.06
NIOZ349.926R.0191.0032	3401	0.19
NIOZ349.926R.0231.0200	478	0.03

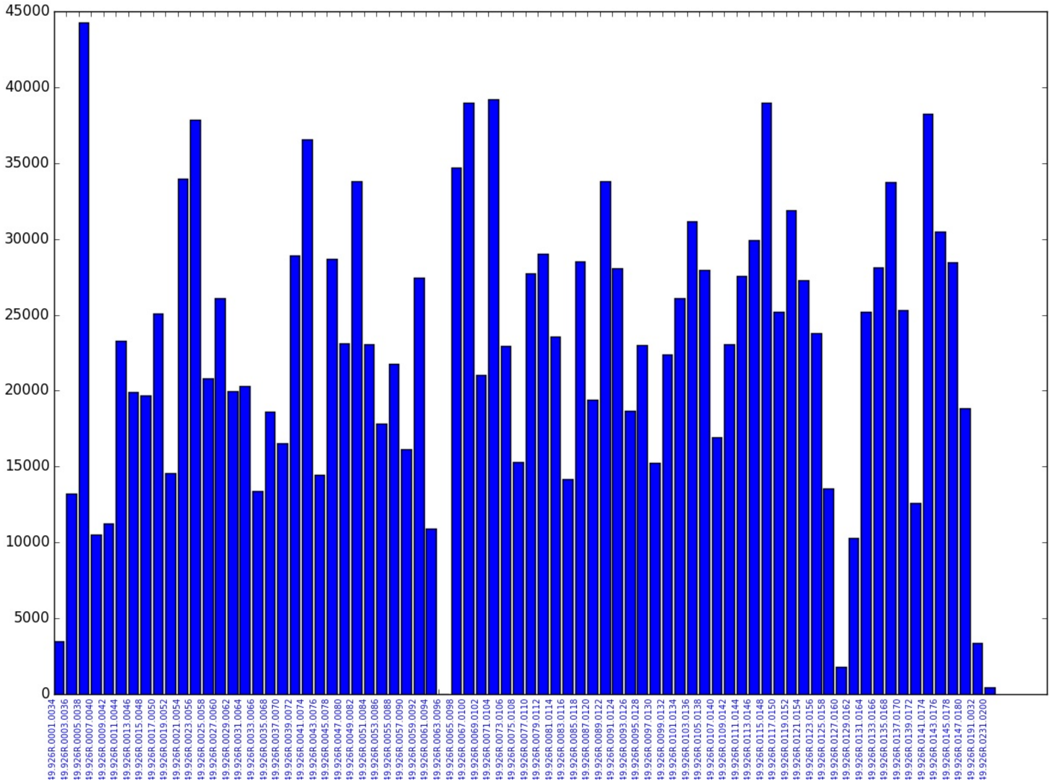

The previous chart shows the number of clean reads per sample. The bars are sorted from left to right, according to the metadata input file.

To see more details about the number of reads per sample in this library, please refer to the file: SARGO_Curacao_18S_unpaired/runs/SARGO_18S_4-4-2024_run1/NIOZ349_data/seqs_fw_rev_filtered.dist.txt

Final counts

Following you can see the final read counts:

File description	Location	Number of reads	Prc(%) vs raw
Raw reads	SARGO_Curacao_18S_unpaired/samples/NIOZ349/rawdata/*.fq	63438353.0	100.00%
Assembled reads	SARGO_Curacao_18S_unpaired/samples/NIOZ349/peared/seqs.assembled.fastq	5218901.0	8.23%
Demultiplexed reads	SARGO_Curacao_18S_unpaired/runs/SARGO_18S_4-4-2024_run1/NIOZ349_data/seqs_fw_rev_accepted.fna	1773914	2.80%
Primer removed	SARGO_Curacao_18S_unpaired/samples/NIOZ349/demultiplexed/unpaired/primer_removed/	1731620.0	2.73%

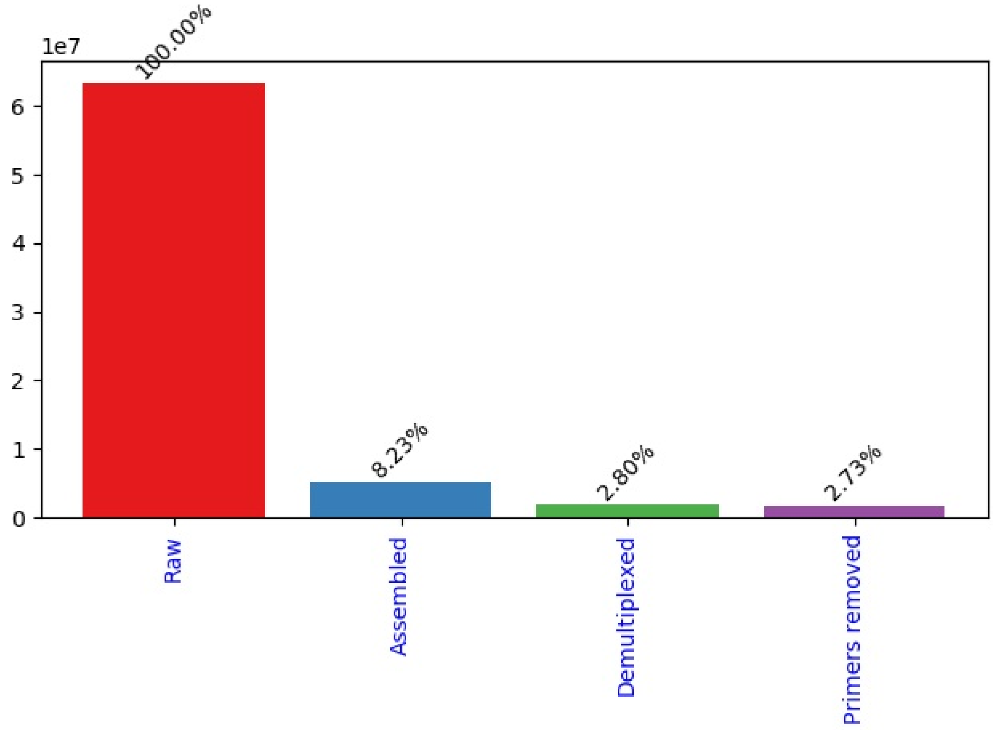

ASV report

Cascabel report on downstream analyses in combination with multiple libraries (if supplied), can be found at the following link: asv_report (SARGO_Curacao_18S_unpaired/runs/SARGO_18S_4-4-2024_run1/asv_report_dada2.html)

References

[FastQC] *(1, 2)* FastQC v0.11.3. Andrews S. (2010). FastQC: a quality control tool for high throughput sequence data

[PEAR] PEAR: a fast and accurate Illumina Paired-End reAd mergeR. Zhang et al (2014) Bioinformatics 30(5): 614-620 | doi:10.1093/bioinformatics/btt593

[QIIME] *(1, 2)* QIIME. Caporaso JG, Kuczynski J, Stombaugh J, Bittinger K, Bushman FD, Costello EK, Fierer N, Gonzalez Pena A, Goodrich JK, Gordon JI, Huttley GA, Kelley ST, Knights D, Koenig JE, Ley RE, Lozupone CA, McDonald D, Muegge BD, Pirrung M, Reeder J, Sevinsky JR, Turnbaugh PJ, Walters WA, Widmann J, Yatsunenko T, Zaneveld J, Knight R. 2010. QIIME allows analysis of high-throughput community sequencing data. Nature Methods 7(5): 335-336.

[Cutadapt] Cutadapt v1.15 .Marcel Martin. Cutadapt removes adapter sequences from high-throughput sequencing reads. EMBnet.Journal, 17(1):10-12, May 2011. http://dx.doi.org/10.14806/ej.17.1.200

[Vsearch] Rognes T, Flouri T, Nichols B, Quince C, Mahé F. (2016) VSEARCH: a versatile open source tool for metagenomics. PeerJ 4:e2584. doi: 10.7717/peerj.2584

Amplicon Analysis Report

CASCABEL is designed to run amplicon sequence analysis across single or multiple read libraries. This report consists of the ASV table creation and taxonomic assignment for all the combined accepted reads of given samples or libraries, if multiple.

Cascabel version: version 6.0.2

Filter and Trim

Once that all the individual libraries were demultiplexed, the fastq files from all the samples for all the libraries were processed together.

The filter and trimming steps were both performed with the **filterAndTrim()** function from the R package dada2, according to user parameters.

Tool: dada2

Version: **[1] '1.19.1'**

Function: filterAndTrim()

Max Expected Errors (maxEE) FW: 3

Max Expected Errors (maxEE) RV: 5

Forward read truncation: 265

Reverse read truncation: 255

Command:

```
Scripts/asvFilter.R $PWD T 265 255 3 5 20 ,truncQ=2, rm.phix=TRUE SARGO_Curacao_18S_unpaired/runs/SARGO_18S_4-4-2024_run1/asv/filter_summary.out
```

Output file:

- Filtered fastq files: SARGO_Curacao_18S_unpaired/runs/SARGO_18S_4-4-2024_run1/<Library>/demultiplexed/filtered/

- Summary: SARGO_Curacao_18S_unpaired/runs/SARGO_18S_4-4-2024_run1/asv/filter_summary.out

Note: To speed up downstream computation, consider tightening maxEE. If too few reads are passing the filter, consider relaxing maxEE, perhaps especially on the reverse reads.

Make sure that your forward and reverse reads overlap after length truncation.

Benchmark info:

s	max_rss	max_vms	max_uss	max_pss	io_in	io_out	mean_load
6335.09	22000.70	33758.45	20776.45	20839.64	184.59	160.45	0.00

Amplicon Sequence Variants

In order to identify ASVs, dada2 workflow require to execute several steps. Following a summary of these steps and its main parameters.

Tool: dada2

Version: **[1] '1.19.1'**

Learn errors

The first step after filtering the reads is to learn the errors from the fastq files.

Function: learnErrors(filteredFQ)

Error plots:

- FW reads error plot:: SARGO_Curacao_18S_unpaired/runs/SARGO_18S_4-4-2024_run1/asv/fw_err.pdf

- RV reads error plot:: SARGO_Curacao_18S_unpaired/runs/SARGO_18S_4-4-2024_run1/asv/rv_err.pdf

ASV inference

The amplicon sequence variant identification consists of a high resolution sample inference from the amplicon data using the learned errors.

Function: dada(filteredFQ, errors, pool='pseudo')

Merge pairs

In this step, forward and reverse reads are paired in order to create full denoised sequences.

Function: mergePairs(dadaF, dadaR)

Min overlap: 12

Max mismatch: 0

Length filtering

Sequences that are much longer or shorter than expected may be the result of non-specific priming.

- Shortest length: 510

- Longest length: 550

Remove chimeras

Sequence variants identified as bimeric are removed, and a bimera-free collection of unique sequences is generated.

Function: removeBimeraDenovo()

Method: consensus

Output files:

- Representative ASV sequences: SARGO_Curacao_18S_unpaired/runs/SARGO_18S_4-4-2024_run1/asv/representative_seq_set.fasta

The total number of different ASVs is: **3783**

Assign taxonomy

Given a set of sequences, assign the taxonomy of each sequence.

Tool: RDP

Function: assignTaxonomy() *implementation of RDP Classifier within dada2*

Reference database: /export/data01/databases/protist_ribosomal_db/pr2_v_5.0.0/pr2_version_5.0.0_SSU_dada2.fasta.gz

Species information: The *'add species'* (add_sps) option from the configuration file is set to **false**. Set it to **true** and supply a *species database* if you want to add species-level annotation to the taxonomic table.

The percentage of successfully assigned ASVs is: **99.81%**

Output file:

- ASV taxonomy assignation: SARGO_Curacao_18S_unpaired/runs/SARGO_18S_4-4-2024_run1/asv/taxonomy_dada2/representative_seq_set_tax_assignments.txt

The previous steps were performed within a Cascabel R script according to the following command:

Command

```
Scripts/asvDada2.R $PWD pseudo 20 T selfConsist=FALSE SARGO_Curacao_18S_unpaired/runs/SARGO_18S_4-4-2024_run1/asv/ 510 550 10 T /export/data01/databases/protist_ribosomal_db/pr2_v_5.0.0/pr2_version_5.0.0_SSU_dada2.fasta.gz /Absolute/Path/to/sp_db/i.e./silva_species_assignment_v132.fa.gz F minBoot=80 12 0 allowMultiple=TRUE
```

Benchmark info:

s	max_rss	max_vms	max_uss	max_pss	io_in	io_out	mean_load
93635.74	17229.71	20556.37	17223.64	17224.19	183.53	2.76	0.00

Make ASV table

Tabulates the number of times an ASV is found in each sample, and adds the taxonomic predictions for each ASV in the last column.

Command:

```
cat SARGO_Curacao_18S_unpaired/runs/SARGO_18S_4-4-2024_run1/asv/taxonomy_dada2/representative_seq_set_tax_assignments.txt | awk 'NR==FNR{if(NR>1){tax=$2;for(i=3;i<=NF;i++){tax=tax";"$i};h[$1]=tax;}next;} {if(FNR==1){print $0"ttaxonomy"}else{print $0"t"h[$1]}}' - SARGO_Curacao_18S_unpaired/runs/SARGO_18S_4-4-2024_run1/asv/asv_table.txt > SARGO_Curacao_18S_unpaired/runs/SARGO_18S_4-4-2024_run1/asv/taxonomy_dada2/asvTable.txt
```

Output file:

- ASV table: SARGO_Curacao_18S_unpaired/runs/SARGO_18S_4-4-2024_run1/asv/taxonomy_dada2/asvTable.txt

Benchmark info:

s	max_rss	max_vms	max_uss	max_pss	io_in	io_out	mean_load
0.06	1.38	110.64	0.22	0.23	0.35	0.00	0.00

Convert ASV table

Convert from txt to the BIOM table format.

Tool: [BIOM]

Version: **biom, version 2.1.6**

Command:

```
biom convert -i SARGO_Curacao_18S_unpaired/runs/SARGO_18S_4-4-2024_run1/asv/taxonomy_dada2/asvTable.txt -o SARGO_Curacao_18S_unpaired/runs/SARGO_18S_4-4-2024_run1/asv/taxonomy_dada2/asvTable.biom --table-type 'OTU table' --table type "OTU table" --to-hdf5 --process-obs-metdata taxonomy
```

Output file:

- Biom format table: SARGO_Curacao_18S_unpaired/runs/SARGO_18S_4-4-2024_run1/asv/taxonomy_dada2/asvTable.biom

Benchmark info:

s	max_rss	max_vms	max_uss	max_pss	io_in	io_out	mean_load
1.62	65.13	5570.57	62.89	62.92	94.76	0.01	0.00

Summarize Taxa

Summarize information of the representation of taxonomic groups within each sample.

Tool: [QIIME] - summarize_taxa.py

Version: **summarize_taxa.py 1.9.1**

Command:

```
summarize_taxa.py -i SARGO_Curacao_18S_unpaired/runs/SARGO_18S_4-4-2024_run1/asv/taxonomy_dada2/otuTable.biom --level 2,3,4,5,6,7 -o SARGO_Curacao_18S_unpaired/runs/SARGO_18S_4-4-2024_run1/asv/taxonomy_dada2/summary/
```

Output file:

- Taxonomy summarized counts at different taxonomy levels: SARGO_Curacao_18S_unpaired/runs/SARGO_18S_4-4-2024_run1/asv/taxonomy_dada2/summary/otuTable_L**N**.txt

Where **N** is the taxonomy level. Default configuration produces levels from 2 to 6.

Benchmark info:

s	max_rss	max_vms	max_uss	max_pss	io_in	io_out	mean_load
9.33	135.88	5671.68	133.53	133.57	0.11	0.44	0.00

Filter ASV table

Filter ASVs from an ASV table based on their observed counts or identifier.

Tool: [QIIME] - filter_otus_from_otu_table.py

Version: **filter_otus_from_otu_table.py 1.9.1**

Minimum observation counts: 2

Command:

```
filter_otus_from_otu_table.py -i SARGO_Curacao_18S_unpaired/runs/SARGO_18S_4-4-2024_run1/asv/taxonomy_dada2/asvTable.biom -o SARGO_Curacao_18S_unpaired/runs/SARGO_18S_4-4-2024_run1/asv/taxonomy_dada2/asvTable_noSingletons.biom -n 2
```

Output file:

- Biom table: SARGO_Curacao_18S_unpaired/runs/SARGO_18S_4-4-2024_run1/asv/taxonomy_dada2/otuTable_noSingletons.biom

Benchmark info:

s	max_rss	max_vms	max_uss	max_pss	io_in	io_out	mean_load
1.95	134.96	5602.78	132.65	132.68	1.89	0.02	0.00

Convert Filtered ASV table

Convert the filtered OTU table from the BIOM table format to a human readable format

Tool: [BIOM]

Version: **biom, version 2.1.6**

Command:

```
biom convert -i SARGO_Curacao_18S_unpaired/runs/SARGO_18S_4-4-2024_run1/otu/taxonomy_dada2/asvTable_noSingletons.biom -o SARGO_Curacao_18S_unpaired/runs/SARGO_18S_4-4-2024_run1/asv/taxonomy_dada2/asvTable_noSingletons.txt --table-type 'OTU table' --header-key taxonomy --to-tsv
```

Output file:

- TSV format table: SARGO_Curacao_18S_unpaired/runs/SARGO_18S_4-4-2024_run1/asv/taxonomy_dada2/asvTable_noSingletons.txt

Benchmark info:

s	max_rss	max_vms	max_uss	max_pss	io_in	io_out	mean_load
1.21	77.89	4793.77	75.59	75.63	1.03	1.52	0.00

Filter representative sequences

Remove sequences according to the filtered OTU biom table.

Tool: [QIIME] - filter_fasta.py

Version: **filter_fasta.py 1.9.1**

Command:

```
filter_fasta.py -f SARGO_Curacao_18S_unpaired/samples/SARGO_18S_4-4-2024_run1/asv/representative_seq_set.fasta -o SARGO_Curacao_18S_unpaired/samples/SARGO_18S_4-4-2024_run1/asv/taxonomy_dada2/representative_seq_set_noSingletons.fasta -b SARGO_Curacao_18S_unpaired/samples/SARGO_18S_4-4-2024_run1/asv/taxonomy_dada2/otuTable_noSingletons.biom
```

Output file:

- Filtered fasta file: SARGO_Curacao_18S_unpaired/samples/SARGO_18S_4-4-2024_run1/asv/taxonomy_dada2/representative_seq_set_noSingletons.fasta

Krona report

Krona allows hierarchical data to be explored with zooming, multi-layered pie charts.

Tool: [Krona]

These charts were created using the ASV table **without** singletons

The report was executed for all the samples.

Each sample is represented on a separated chart (same html report).

You can see the report at the following link:

- Krona report: kreport

Or access the html file at:

- Krona html file: SARGO_Curacao_18S_unpaired/runs/SARGO_18S_4-4-2024_run1/asv/taxonomy_dada2/krona_report.html

Benchmark info:

s	max_rss	max_vms	max_uss	max_pss	io_in	io_out	mean_load
3.08	28.57	383.39	21.77	22.78	5.70	3.10	0.00

Final counts

Following the read counts:

File description	Location	#	(%)
Demultiplexed reads	SARGO_Curacao_18S_unpaired/runs/SARGO_18S_4-4-2024_run1/<SAMPLE>_data/demultiplexed/*.fastq.gz	2426367	100%
QA filtered & trimmed reads	SARGO_Curacao_18S_unpaired/runs/SARGO_18S_4-4-2024_run1/<LIBRARY>_data/demultiplexed/filtered/*.fastq.gz	2207150	90.97%
Denoised FW reads	*No intermediate file generated*	2199570	90.65%
Denoised RV reads	*NO intermediate file generated*	2199133	90.63%
Merged and full denoised reads	*No intermediate file generated*	2193429	90.40%
Length filtered	*No intermediate file generated*	2193429	90.40%
Chimera removed	*No intermediate file generated*	2142745	88.31%
ASV table	SARGO_Curacao_18S_unpaired/runs/SARGO_18S_4-4-2024_run1/asv/asvTable.txt	3783	100%
Taxonomy assignation	SARGO_Curacao_18S_unpaired/runs/SARGO_18S_4-4-2024_run1/asv/taxonomy_dada2/representative_seq_set_tax_assignments.txt	3776	99.81%
ASV table (no singletons: a > 2)	SARGO_Curacao_18S_unpaired/runs/SARGO_18S_4-4-2024_run1/asv/taxonomy_dada2/asvTable_noSingletons.txt	3077	81.34%
Assigned no singletons	SARGO_Curacao_18S_unpaired/runs/SARGO_18S_4-4-2024_run1/otu/taxonomy_qiime/asvTable_noSingletons.txt	3073	99.87%

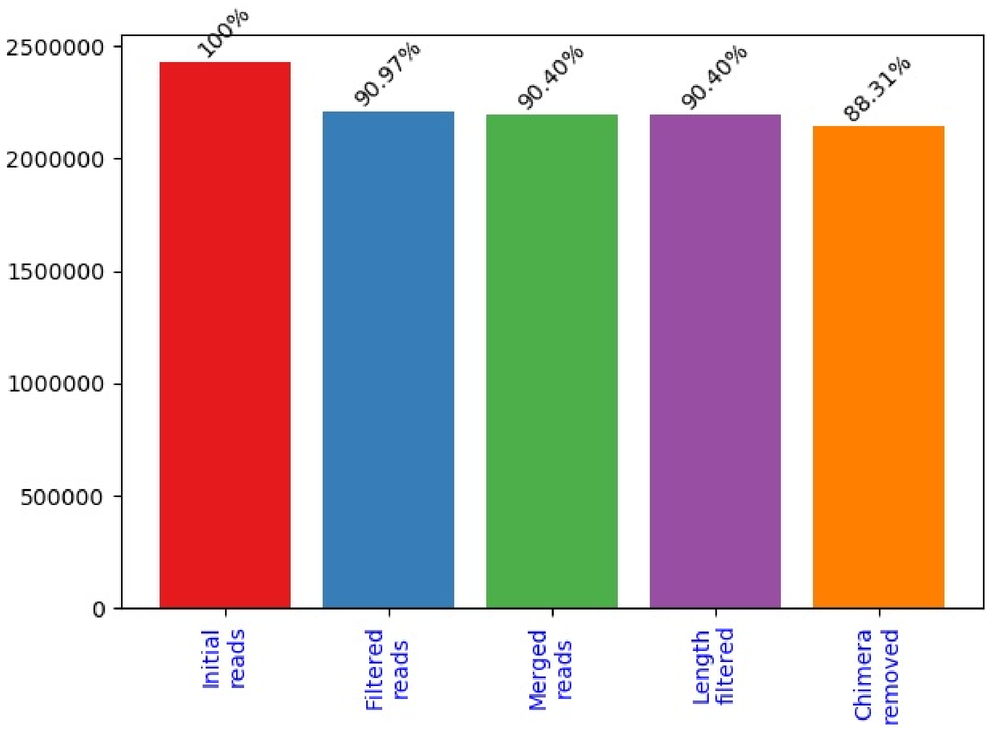

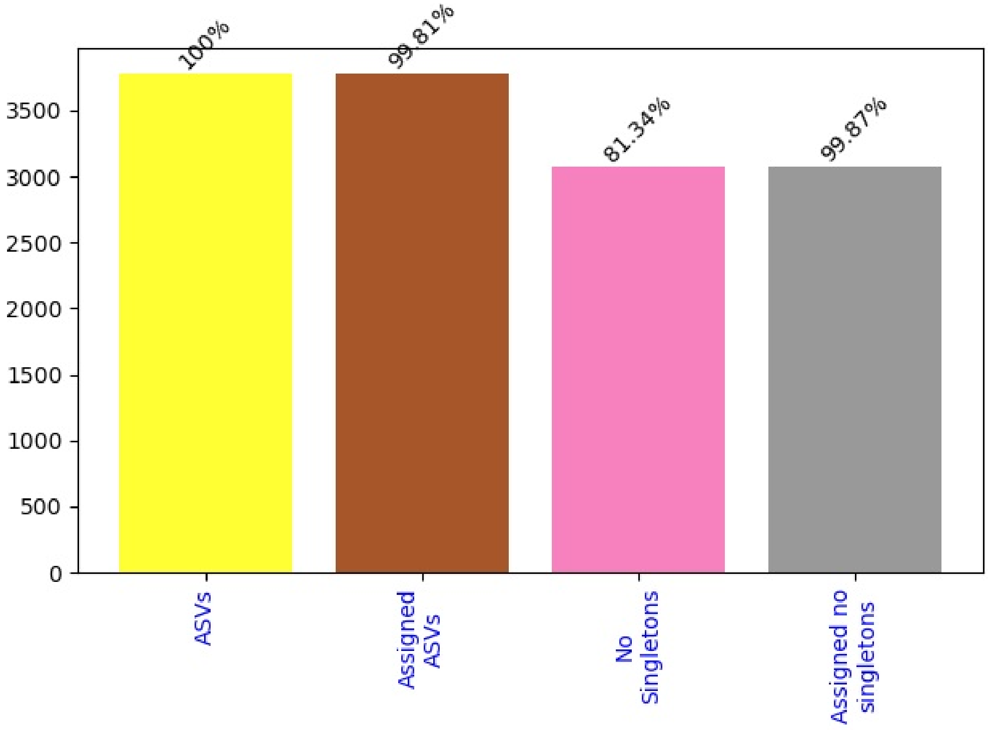

Note:

- Assigned ASVs percentage is the amount of successfully assigned ASVs.

- No singletons percentage is the percentage of no singletons ASVs in reference to the complete ASV table.

- Assigned No singletons is the amount of successfully no singletons assigned ASVs.

References

[QIIME] *(1, 2, 3)* QIIME. Caporaso JG, Kuczynski J, Stombaugh J, Bittinger K, Bushman FD, Costello EK, Fierer N, Gonzalez Pena A, Goodrich JK, Gordon JI, Huttley GA, Kelley ST, Knights D, Koenig JE, Ley RE, Lozupone CA, McDonald D, Muegge BD, Pirrung M, Reeder J, Sevinsky JR, Turnbaugh PJ, Walters WA, Widmann J, Yatsunenko T, Zaneveld J, Knight R. 2010. QIIME allows analysis of high-throughput community sequencing data. Nature Methods 7(5): 335-336.

[Cutadapt] Cutadapt v1.15 .Marcel Martin. Cutadapt removes adapter sequences from high-throughput sequencing reads. EMBnet.Journal, 17(1):10-12, May 2011. http://dx.doi.org/10.14806/ej.17.1.200

[vsearch] Rognes T, Flouri T, Nichols B, Quince C, Mahé F. (2016) VSEARCH: a versatile open source tool for metagenomics. PeerJ 4:e2584. doi: 10.7717/peerj.2584

[Krona] Ondov BD, Bergman NH, and Phillippy AM. Interactive metagenomic visualization in a Web browser. BMC Bioinformatics. 2011 Sep 30; 12(1):385.

[BIOM] *(1, 2)* The Biological Observation Matrix (BIOM) format or: how I learned to stop worrying and love the ome-ome. Daniel McDonald, Jose C. Clemente, Justin Kuczynski, Jai Ram Rideout, Jesse Stombaugh, Doug Wendel, Andreas Wilke, Susan Huse, John Hufnagle, Folker Meyer, Rob Knight, and J. Gregory Caporaso.GigaScience 2012, 1:7. doi:10.1186/2047-217X-1-7

[RDP] Wang, Q, G. M. Garrity, J. M. Tiedje, and J. R. Cole. 2007. Naive Bayesian Classifier for Rapid Assignment of rRNA Sequences into the New Bacterial Taxonomy. Appl Environ Microbiol. 73(16):5261-7.

[dada2] Callahan BJ, McMurdie PJ, Rosen MJ, Han AW, Johnson AJA, Holmes SP (2016). DADA2: High-resolution sample inference from Illumina amplicon data. Nature Methods, 13, 581-583. doi: 10.1038/nmeth.3869.
