# Supplementary material for: Nutrient‐driven growth and microbiome shifts in the brown alga Sargassum fluitans III
Source: J Phycol. 2025 Jun 20;61(4):933–50. doi: 10.1111/jpy.70045 (PMC12351368; doi:10.1111/jpy.70045)
Supplement: Supplementary file 5 — Appendix S6. Detrended Correspondence Analysis (DCA) showing the distribution of the bacterial community samples of S. fluitans III after 6 days (n = 3): Control (C), Nitrate (N), Phosphate (P) and Nitrate and Phosphate (NP). Amplicon Sequence Variants (ASV) are shown with red crosses and samples of bacterial community compositions are shown with black circles. [file JPY-61-933-s003.docx]

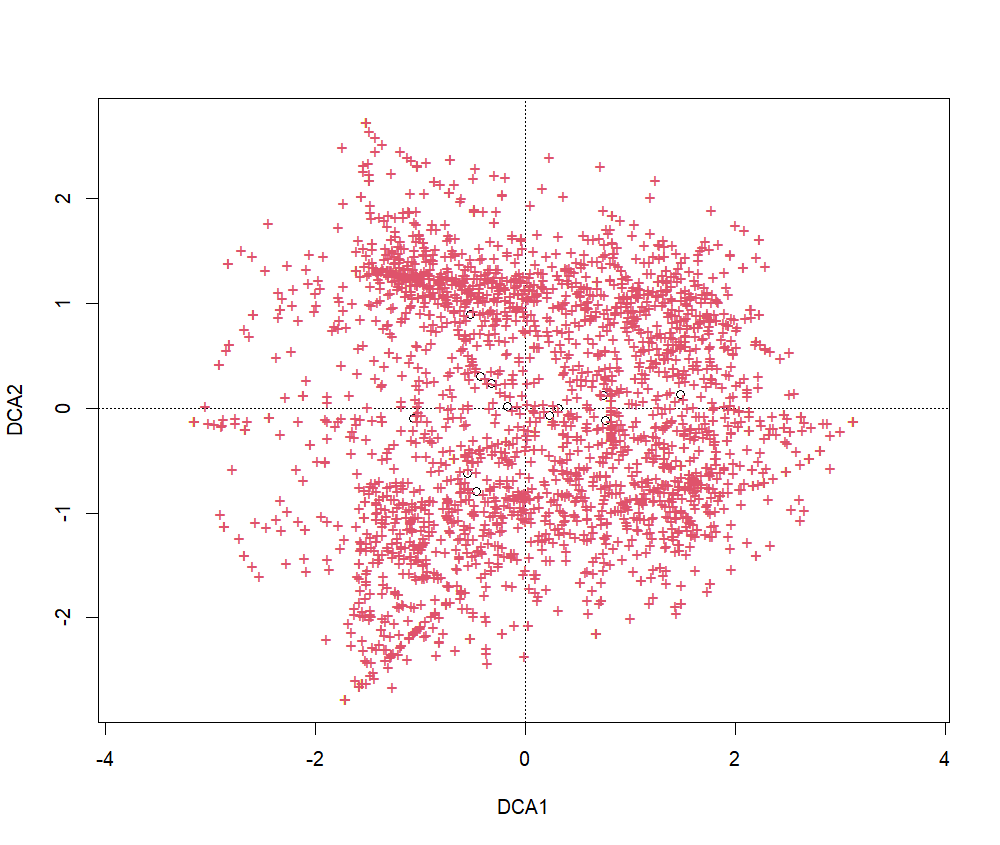


**Figure S6:** Detrended Correspondence Analysis (DCA) showing the distribution of the bacterial community samples of *S. fluitans* III after 6 days (n=3): Control (C), Nitrate (N), Phosphate (P) and Nitrate and Phosphate (NP). Amplicon Sequence Variants (ASV) are shown with red crosses and samples of bacterial community compositions are shown with black circles.
