# Supplementary material for: Nutrient‐driven growth and microbiome shifts in the brown alga Sargassum fluitans III
Source: J Phycol. 2025 Jun 20;61(4):933–50. doi: 10.1111/jpy.70045 (PMC12351368; doi:10.1111/jpy.70045)
Supplement: Supplementary file 7 — Appendix S8. Ratios of elemental C:N, C:P and C:P in Sargassum fluitans III for different time points during the experiment for treatments (n = 3): Control (C), Nitrate (N), Phosphate (P) and Nitrate and Phosphate (NP). Error bars indicate the standard error of the mean (n = 3). Appendix S9: Accumulative growth in length of Sargassum fluitans III for different time points during the experiment for treatments (n = 5): Control, Nitrate, Phosphate and Nitrate + Phosphate. Error bars indicate the standard error of the mean (n = 3). [file JPY-61-933-s004.docx]

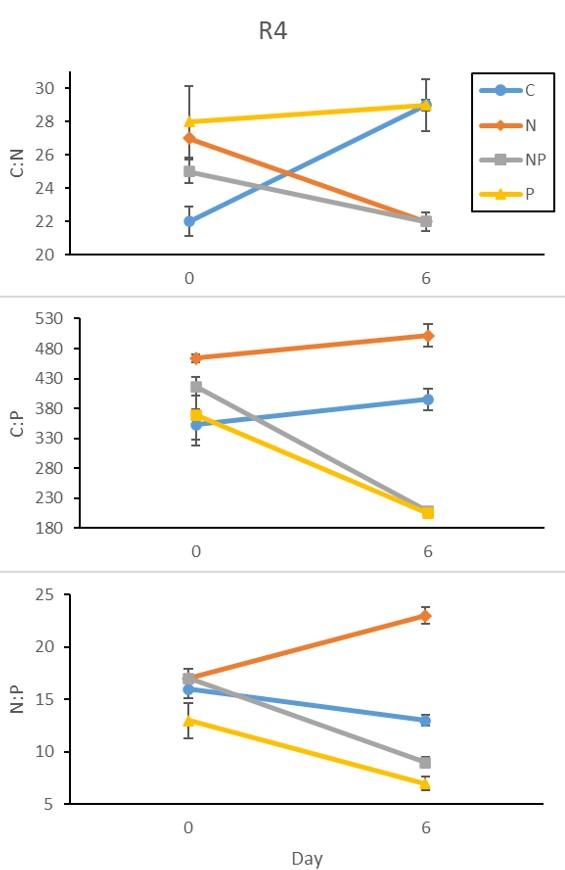


**Figure S8:** ratios of elemental C:N, C:P and C:P in *Sargassum fluitans* III for different time points during the experiment for treatments (n=3): Control (C), Nitrate (N), Phosphate (P) and Nitrate and Phosphate (NP). Error bars indicate the standard error of the mean (n=3).


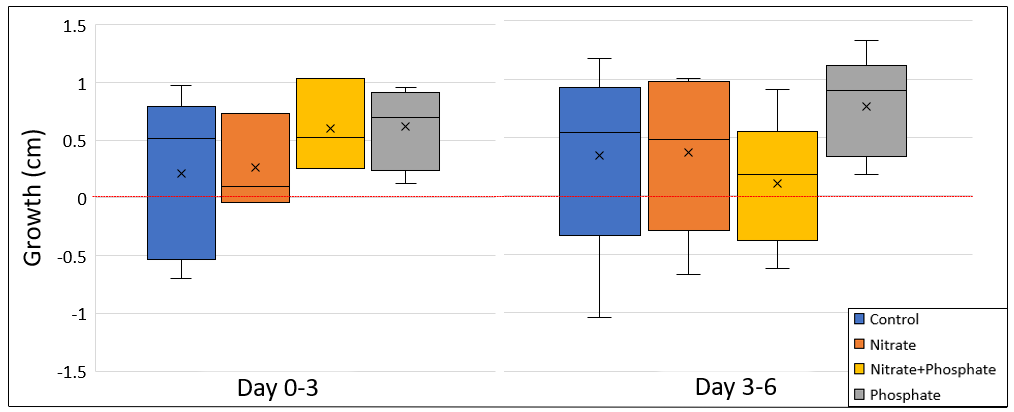


**Figure S9:** Accumulative growth in length of *Sargassum fluitans* III for different time points during the experiment for treatments (n=5): Control, Nitrate, Phosphate and Nitrate + Phosphate. Error bars indicate the standard error of the mean (n=3).
