# Supplementary material for: Nutrient‐driven growth and microbiome shifts in the brown alga Sargassum fluitans III
Source: J Phycol. 2025 Jun 20;61(4):933–50. doi: 10.1111/jpy.70045 (PMC12351368; doi:10.1111/jpy.70045)
Supplement: Supplementary file 9 — Appendix S12. Box plots of shown genera in Figure 6. [file JPY-61-933-s001.pdf]

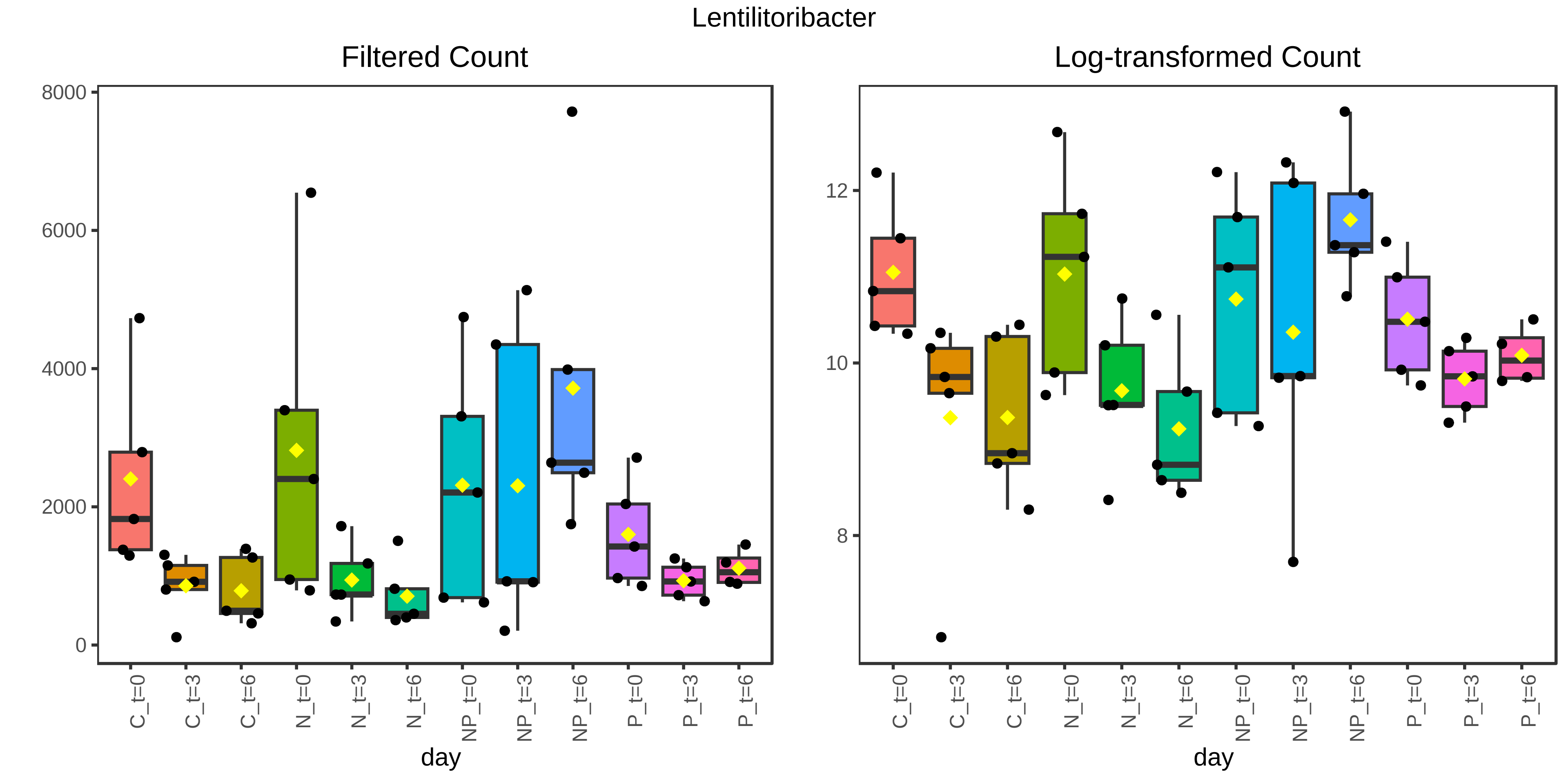

Lentilitoribacter
Filtered Count
Log-transformed Count
8000
6000
4000
2000
0
12
10
8
C_t=0
C_t=3
C_t=6
N_t=0
N_t=3
N_t=6
NP_t=0
NP_t=3
NP_t=6
P_t=0
P_t=3
P_t=6
day

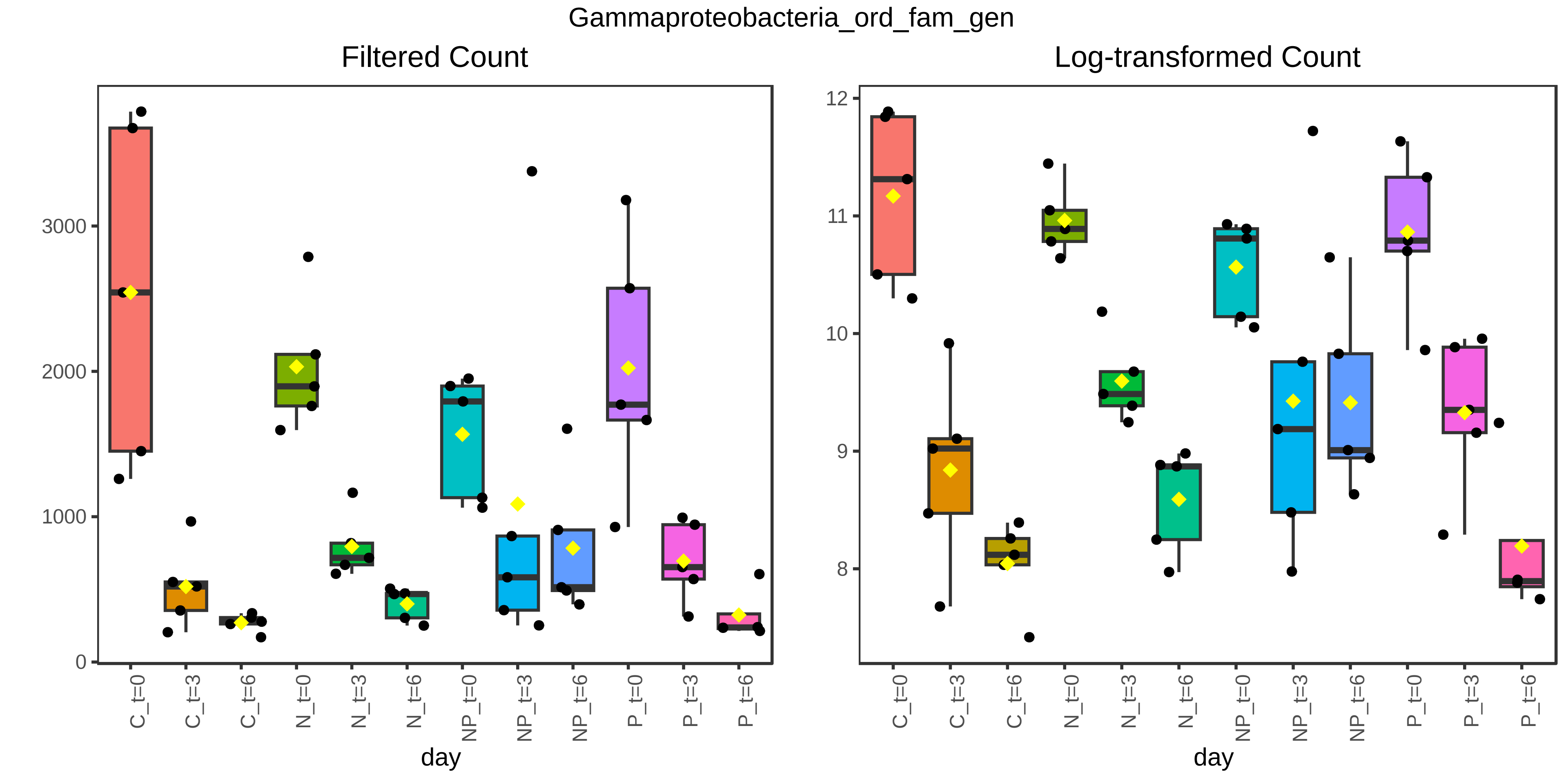

Gammaproteobacteria_ord_fam_gen
Filtered Count
Log-transformed Count
0
1000
2000
3000
8
9
10
11
12
C_t=0
C_t=3
C_t=6
N_t=0
N_t=3
N_t=6
NP_t=0
NP_t=3
NP_t=6
P_t=0
P_t=3
P_t=6
day

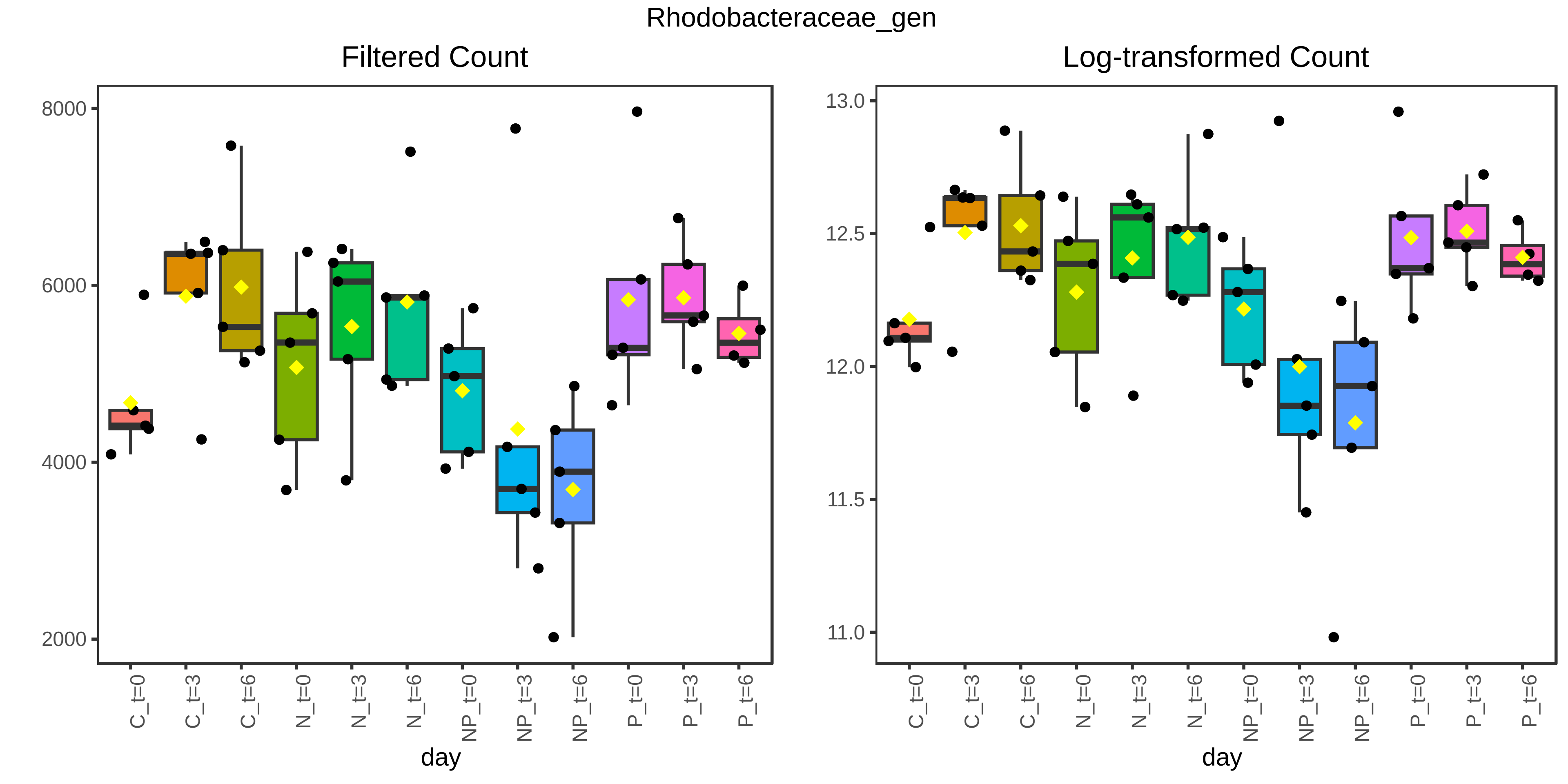
Rhodobacteraceae_gen
Filtered Count
Log-transformed Count
8000
6000
4000
2000
13.0
12.5
12.0
11.5
11.0
C_t=0
C_t=3
C_t=6
N_t=0
N_t=3
N_t=6
NP_t=0
NP_t=3
NP_t=6
P_t=0
P_t=3
P_t=6
day

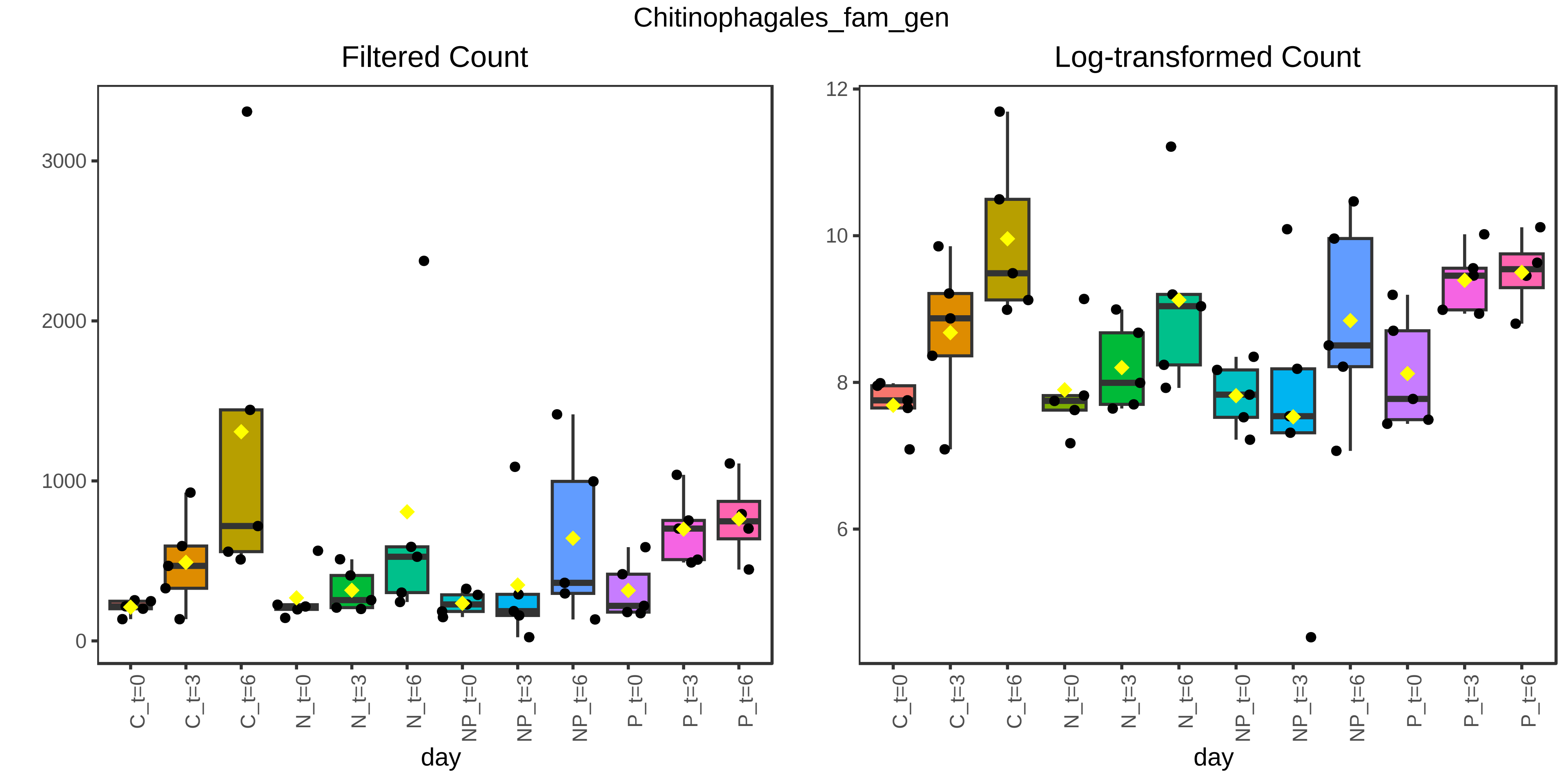
Chitinophagales_fam_gen
Filtered Count
Log-transformed Count
0
1000
2000
3000
6
8
10
12
C_t=0
C_t=3
C_t=6
N_t=0
N_t=3
N_t=6
NP_t=0
NP_t=3
NP_t=6
P_t=0
P_t=3
P_t=6
day

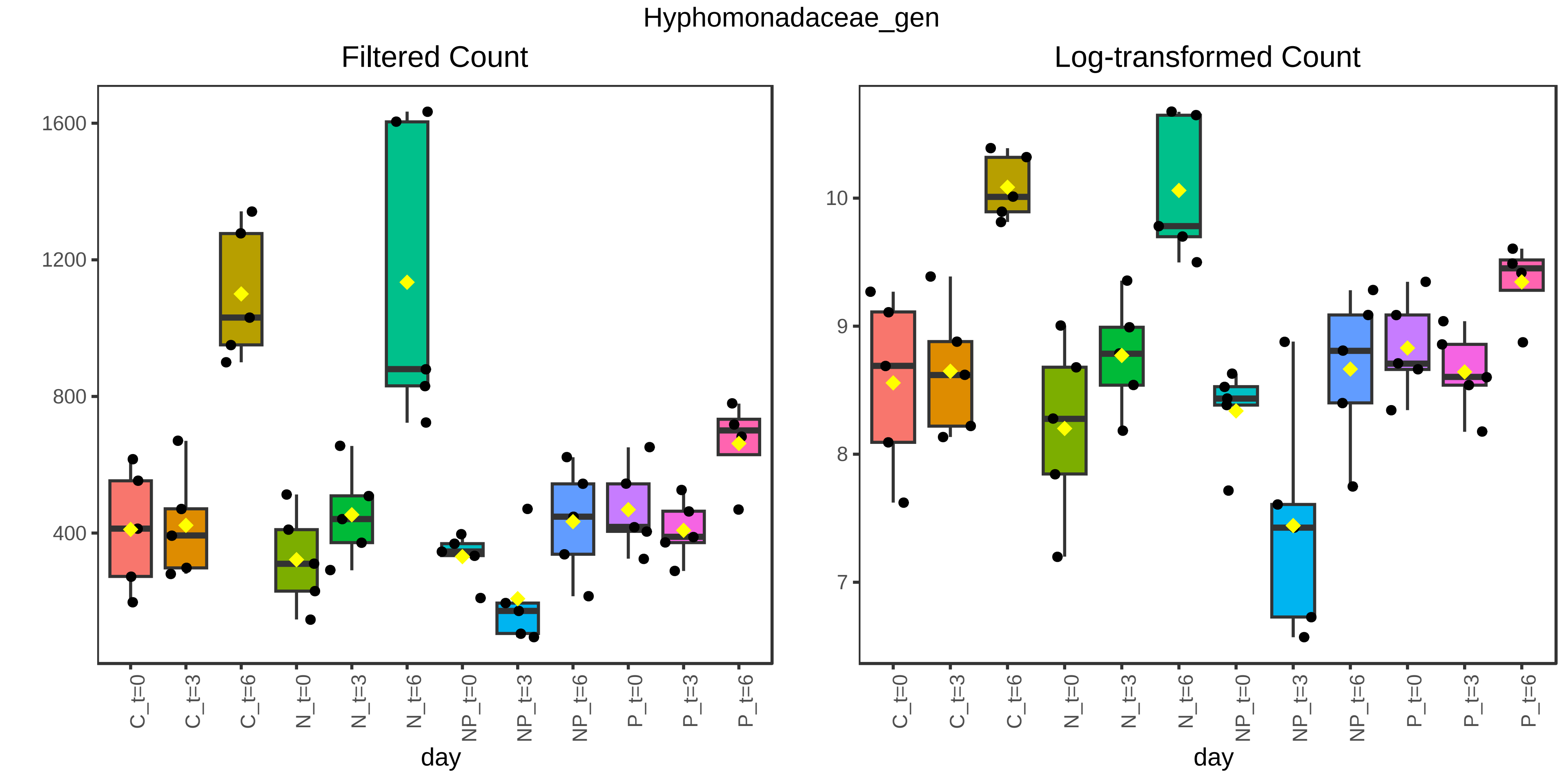
Hyphomonadaceae_gen
Filtered Count
Log-transformed Count
1600
1200
800
400
10
9
8
7
C_t=0
C_t=3
C_t=6
N_t=0
N_t=3
N_t=6
NP_t=0
NP_t=3
NP_t=6
P_t=0
P_t=3
P_t=6
day

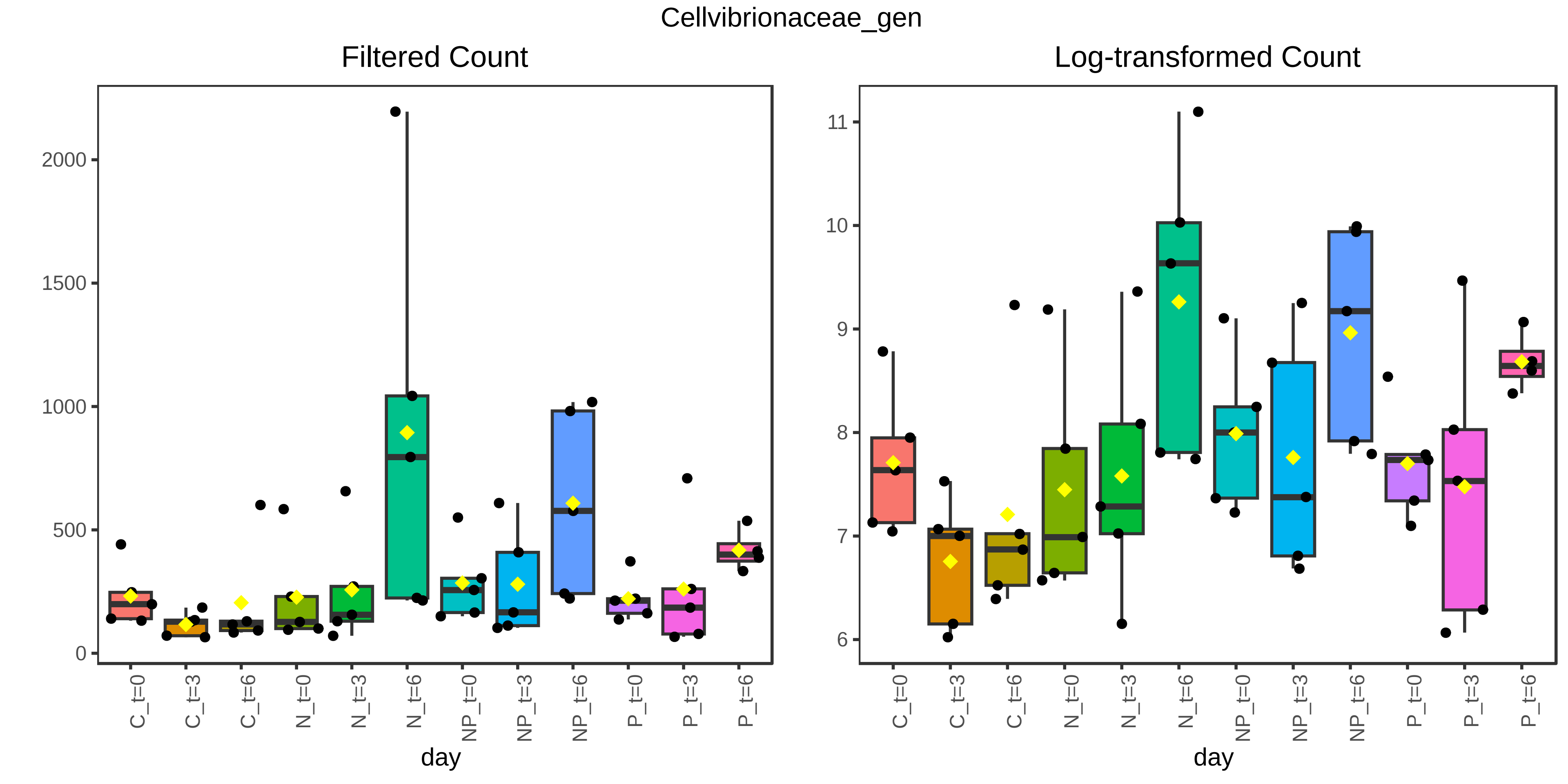

Cellvibrionaceae_gen
Filtered Count
Log-transformed Count
0
500
1000
1500
2000
6
7
8
9
10
11
C_t=0
C_t=3
C_t=6
N_t=0
N_t=3
N_t=6
NP_t=0
NP_t=3
NP_t=6
P_t=0
P_t=3
P_t=6
day

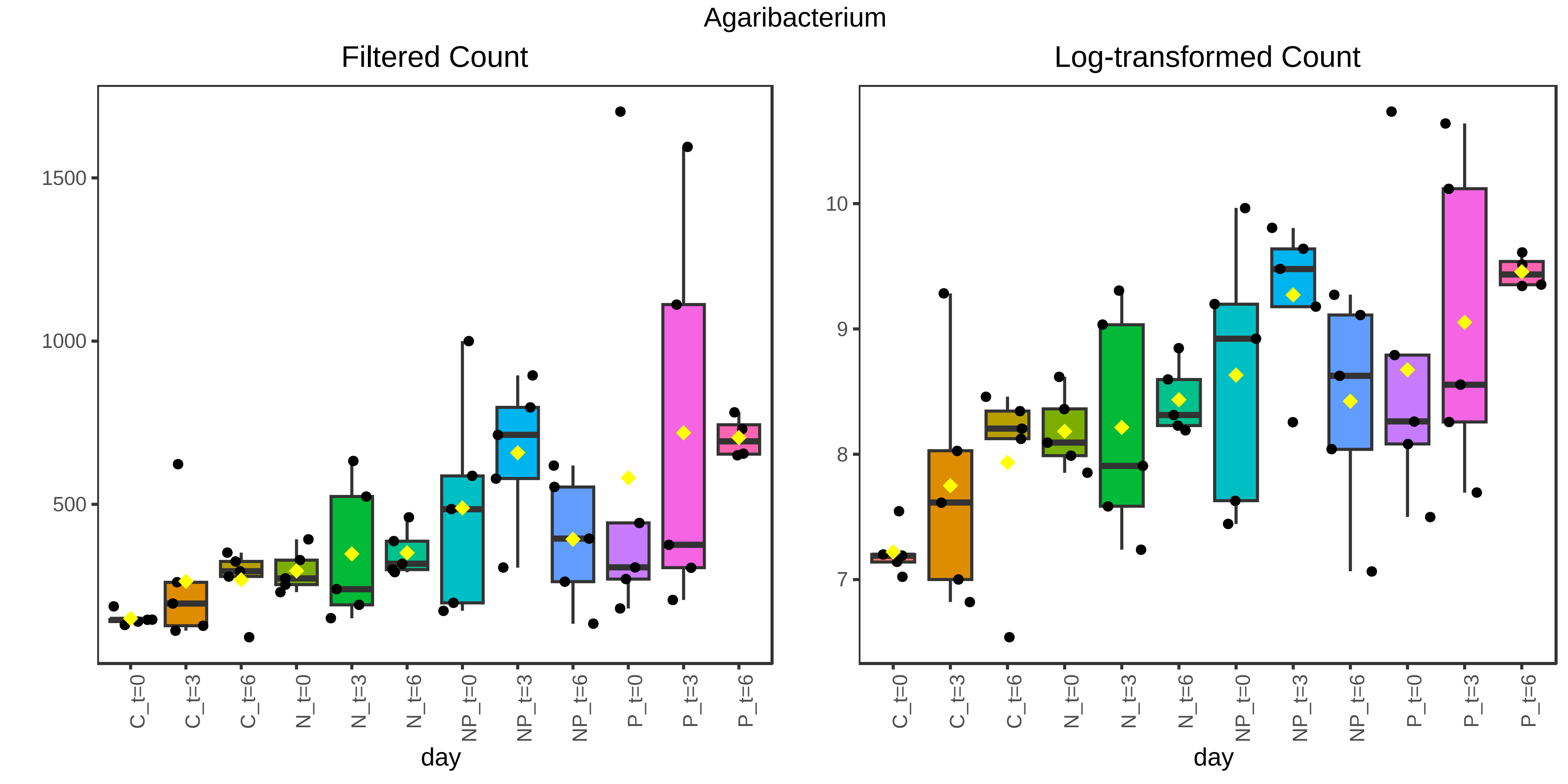

Agaribacterium
Filtered Count
Log-transformed Count
1500
1000
500
10
9
8
7
C_t=0
C_t=3
C_t=6
N_t=0
N_t=3
N_t=6
NP_t=0
NP_t=3
NP_t=6
P_t=0
P_t=3
P_t=6
day

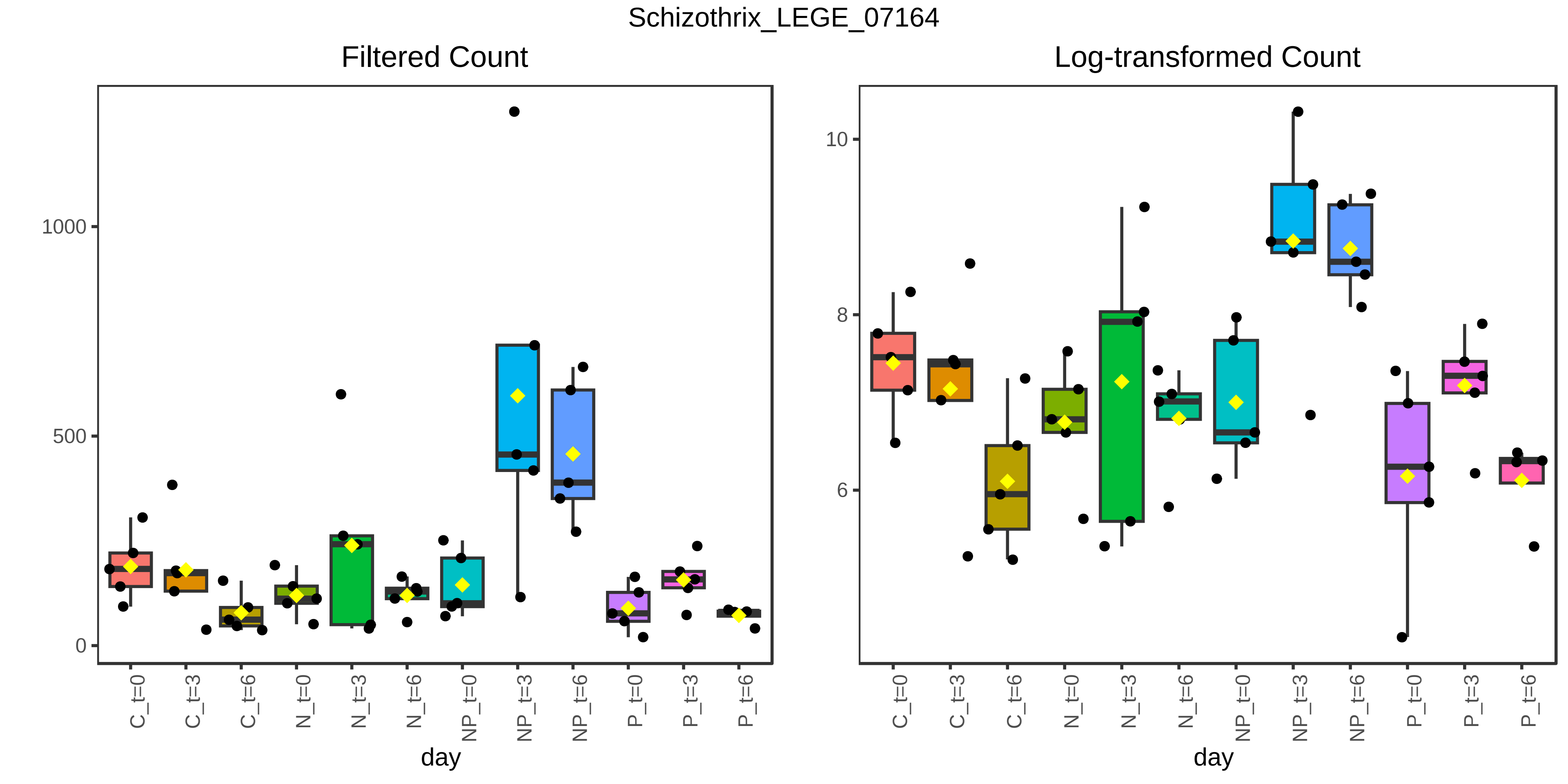
Schizothrix_LEGE_07164
Filtered Count
Log-transformed Count
1000
500
0
10
8
6
C_t=0
C_t=3
C_t=6
N_t=0
N_t=3
N_t=6
NP_t=0
NP_t=3
NP_t=6
P_t=0
P_t=3
P_t=6
day

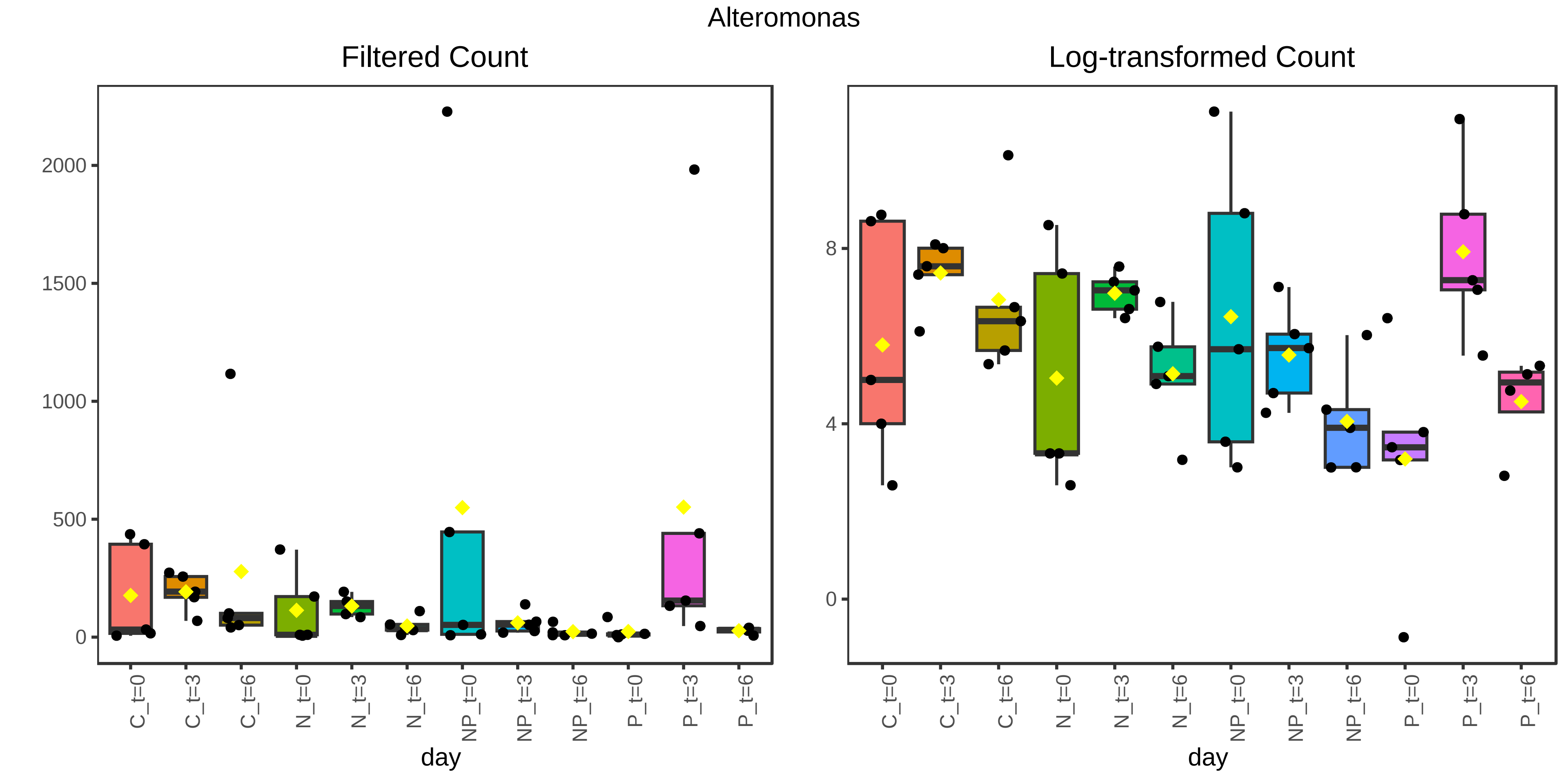

Alteromonas
Filtered Count
Log-transformed Count
0
500
1000
1500
2000
0
4
8
C_t=0
C_t=3
C_t=6
N_t=0
N_t=3
N_t=6
NP_t=0
NP_t=3
NP_t=6
P_t=0
P_t=3
P_t=6
day

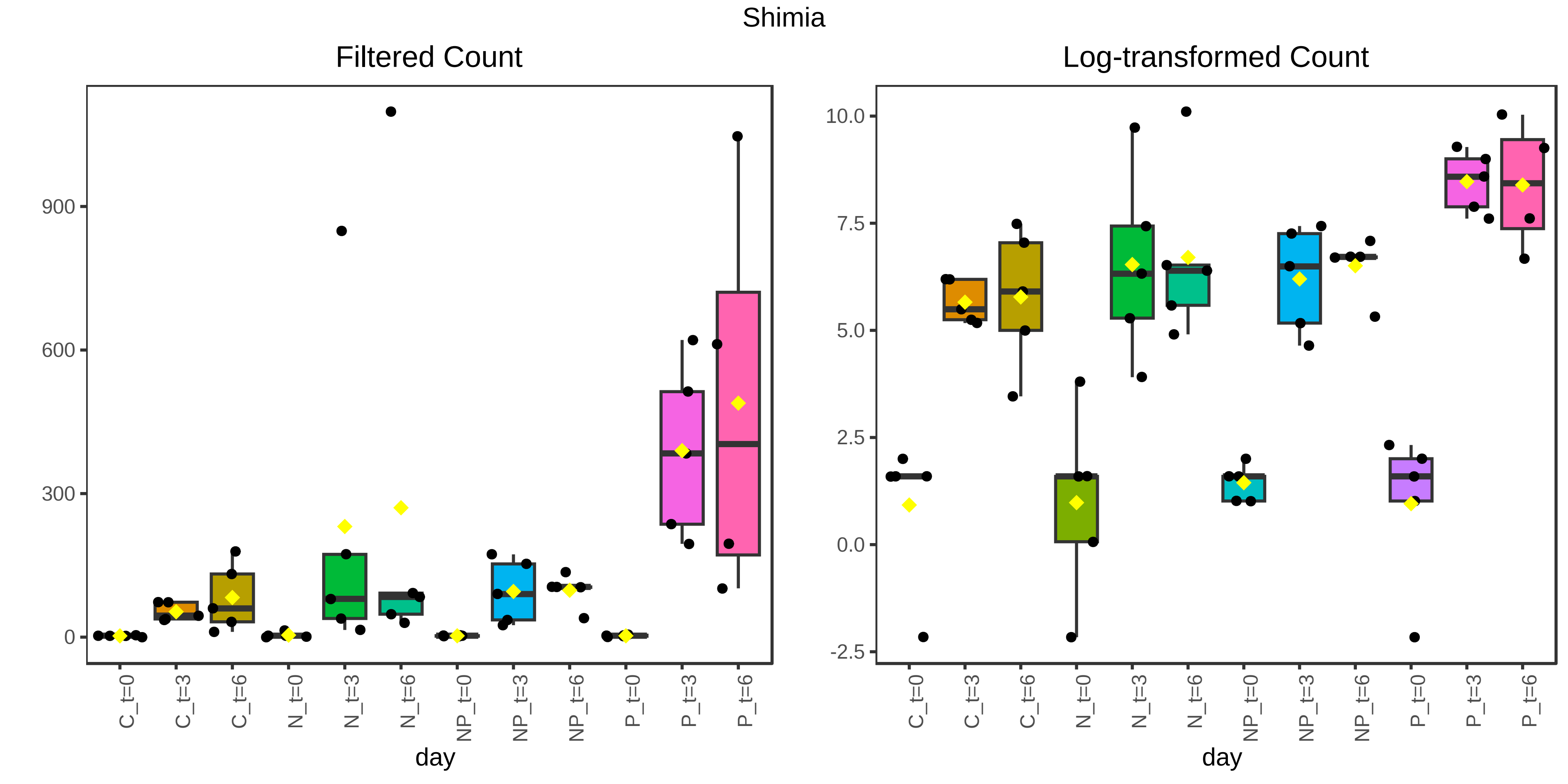
Shimia
Filtered Count
Log-transformed Count
900
600
300
0
10.0
7.5
5.0
2.5
0.0
-2.5
C_t=0
C_t=3
C_t=6
N_t=0
N_t=3
N_t=6
NP_t=0
NP_t=3
NP_t=6
P_t=0
P_t=3
P_t=6
day
day

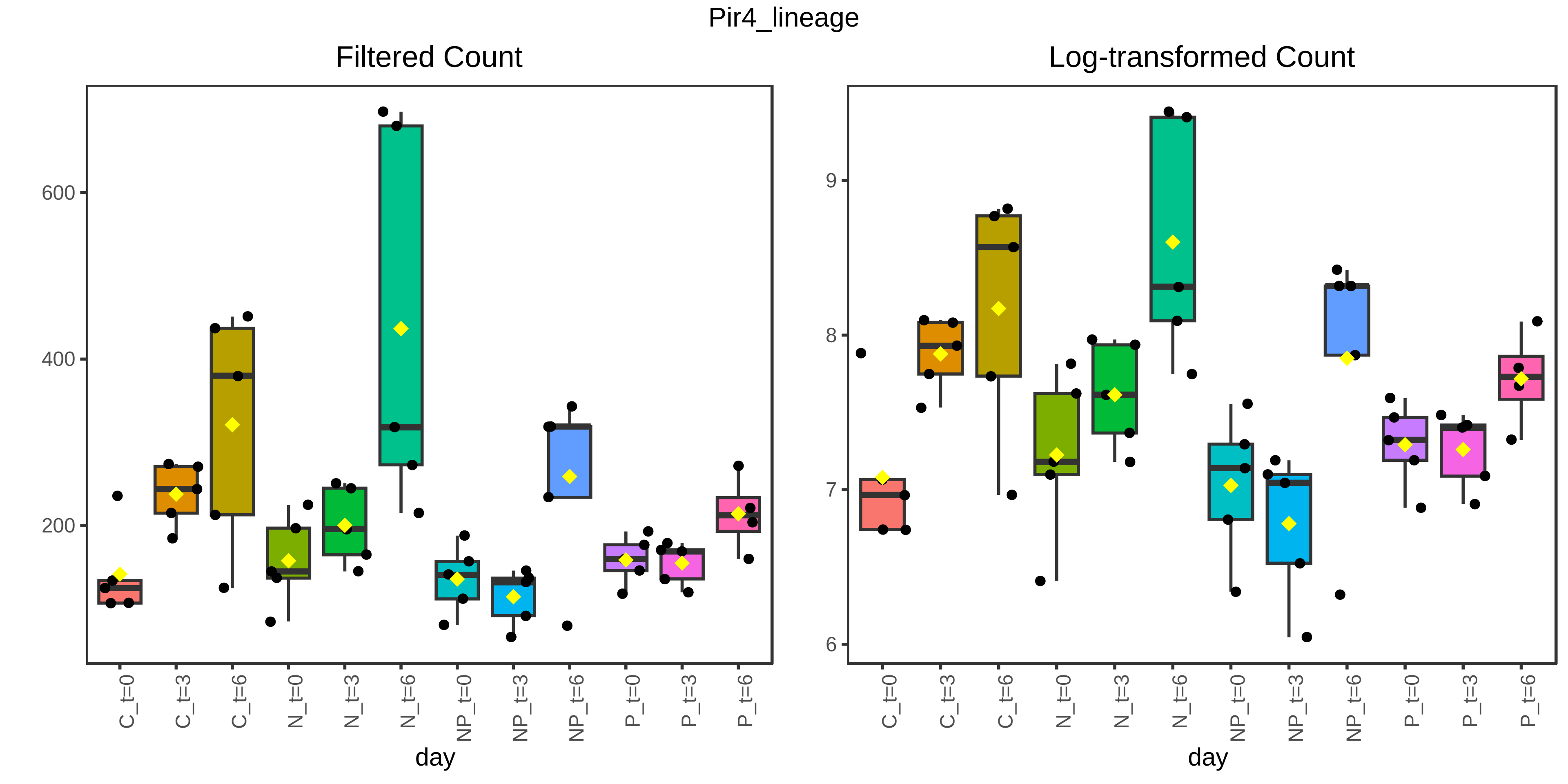
Pir4_lineage
Filtered Count
Log-transformed Count
600
400
200
9
8
7
6
C_t=0
C_t=3
C_t=6
N_t=0
N_t=3
N_t=6
NP_t=0
NP_t=3
NP_t=6
P_t=0
P_t=3
P_t=6
day
day

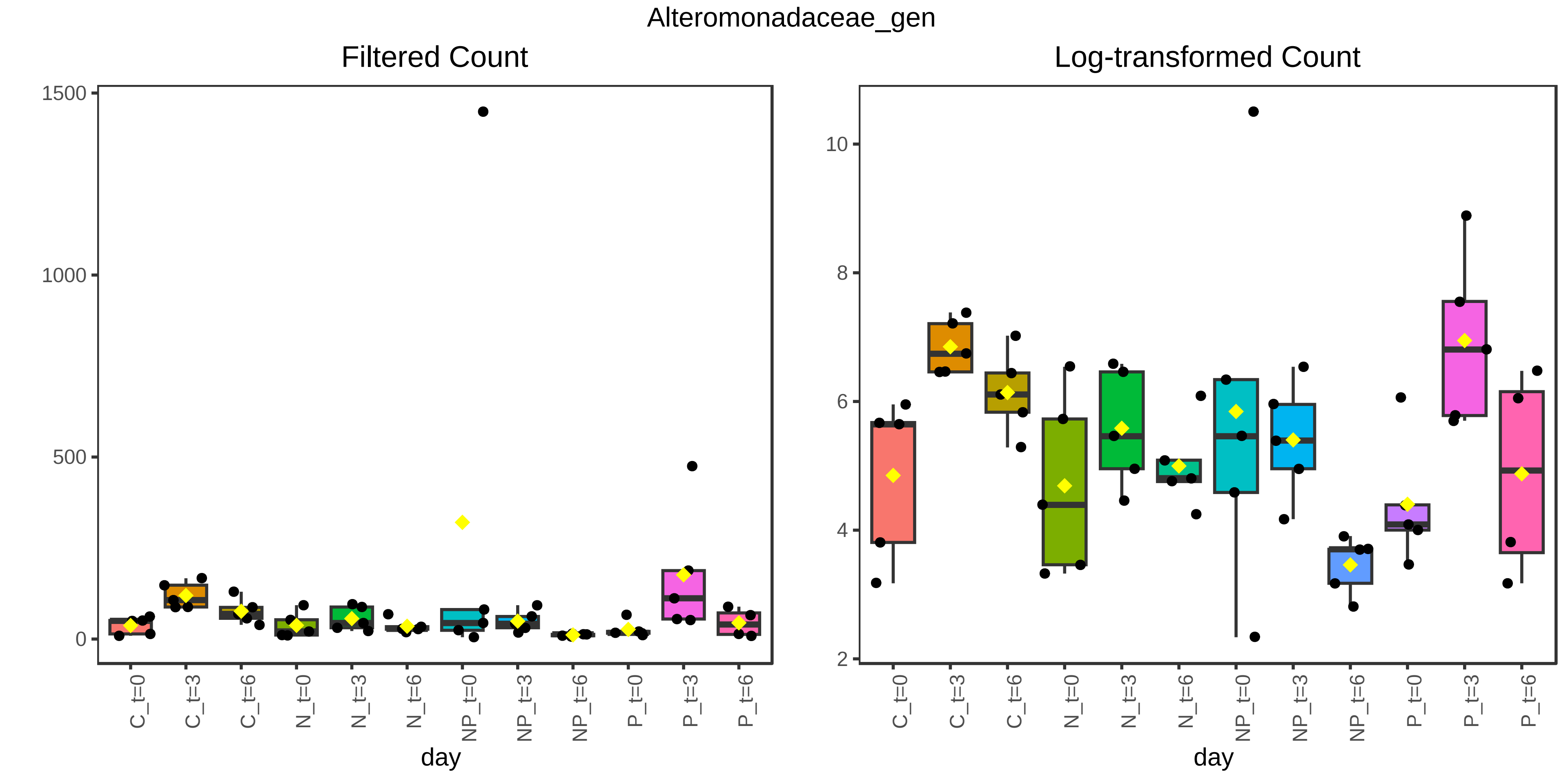

Alteromonadaceae_gen
Filtered Count
Log-transformed Count
1500
1000
500
0
10
8
6
4
2
C_t=0
C_t=3
C_t=6
N_t=0
N_t=3
N_t=6
NP_t=0
NP_t=3
NP_t=6
P_t=0
P_t=3
P_t=6
day

C1_B045

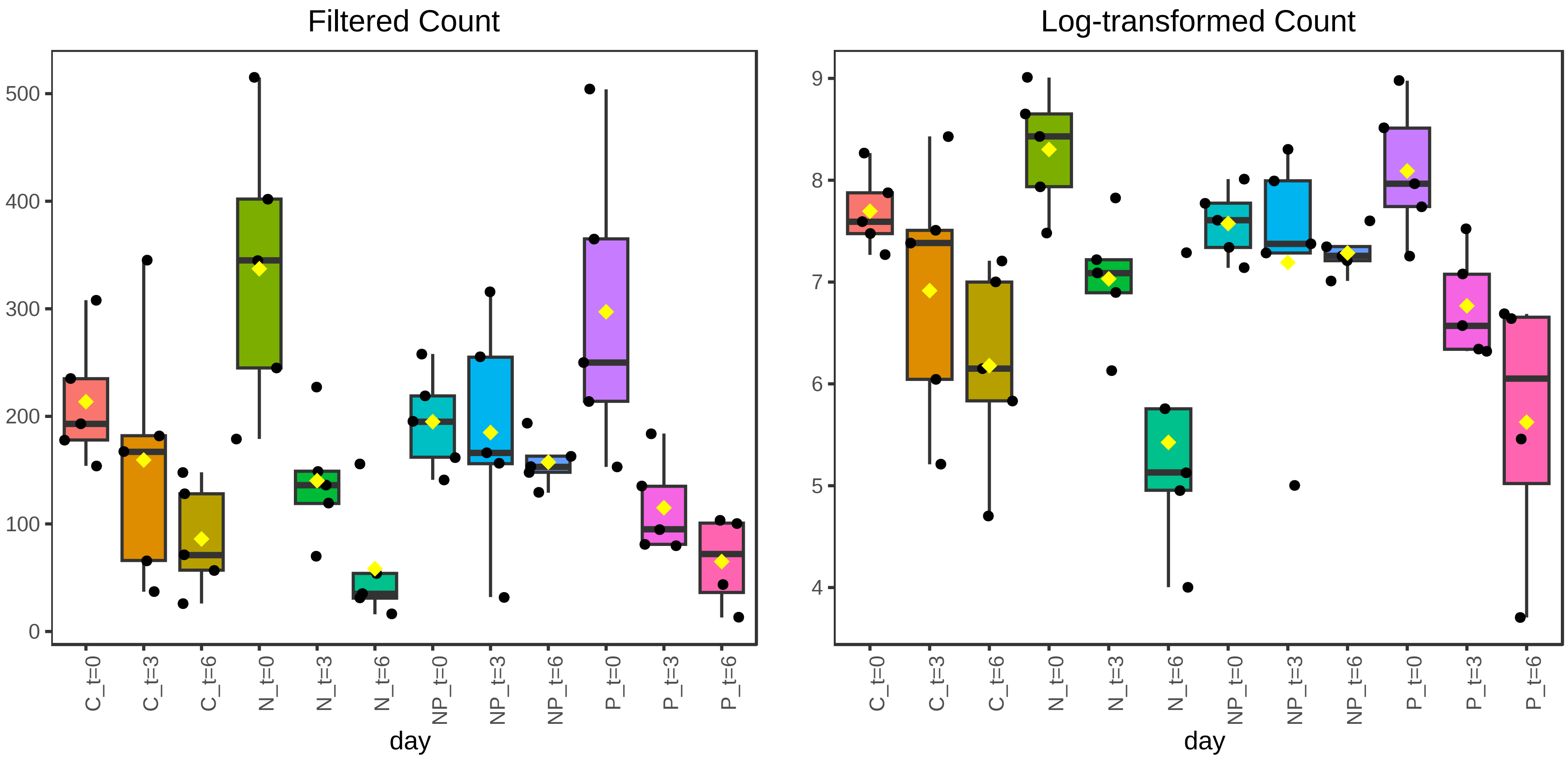

Filtered Count
Log-transformed Count
0
100
200
300
400
500
4
5
6
7
8
9
C_t=0
C_t=3
C_t=6
N_t=0
N_t=3
N_t=6
NP_t=0
NP_t=3
NP_t=6
P_t=0
P_t=3
P_t=6
day

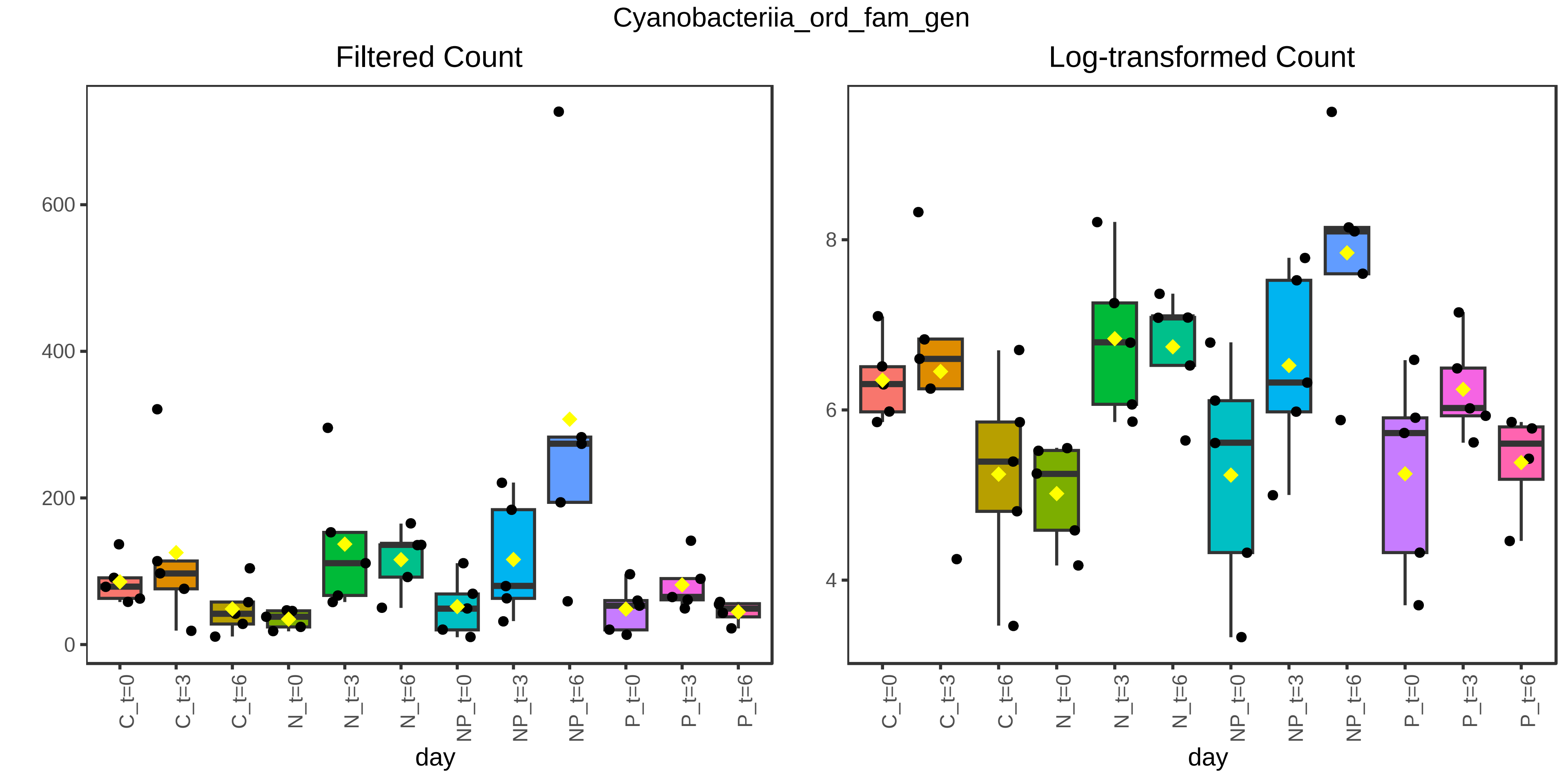
Cyanobacteriia_ord_fam_gen
Filtered Count
Log-transformed Count
600
400
200
0
8
6
4
C_t=0
C_t=3
C_t=6
N_t=0
N_t=3
N_t=6
NP_t=0
NP_t=3
NP_t=6
P_t=0
P_t=3
P_t=6
day
day

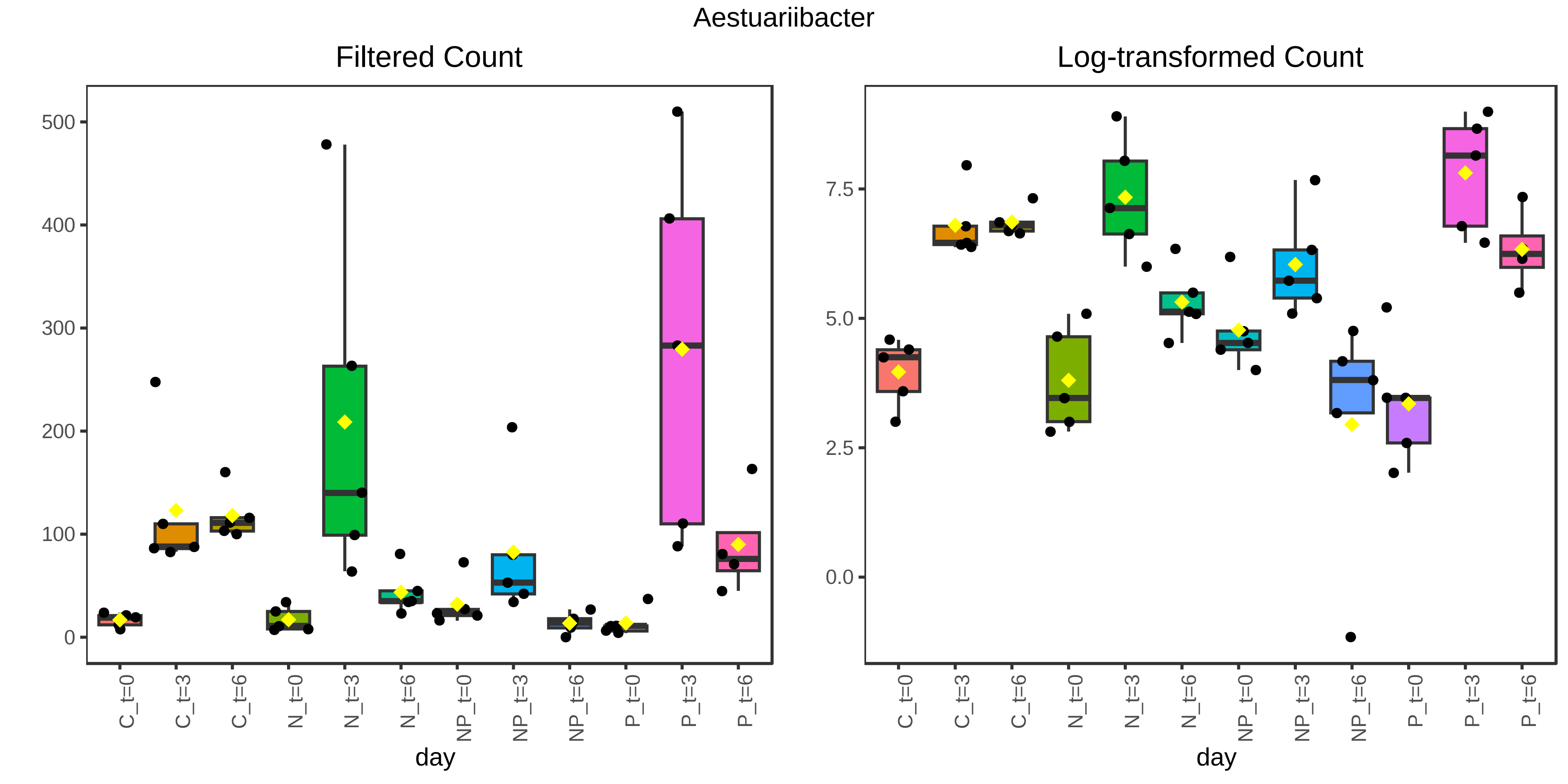
Aestuariibacter
Filtered Count
Log-transformed Count
500
400
300
200
100
0
7.5
5.0
2.5
0.0
C_t=0
C_t=3
C_t=6
N_t=0
N_t=3
N_t=6
NP_t=0
NP_t=3
NP_t=6
P_t=0
P_t=3
P_t=6
day

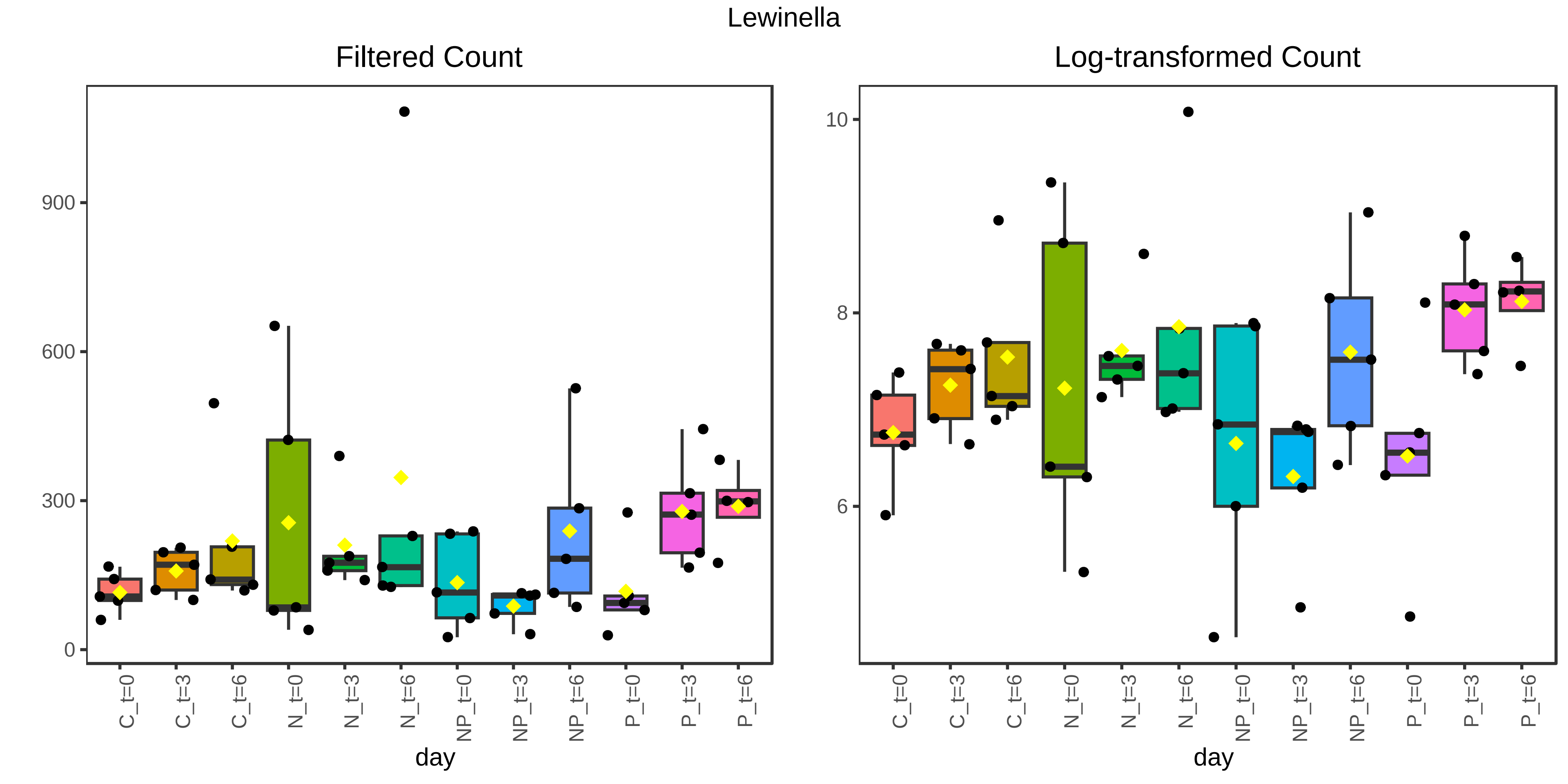

Lewinella
Filtered Count
Log-transformed Count
900
600
300
0
10
8
6
C_t=0
C_t=3
C_t=6
N_t=0
N_t=3
N_t=6
NP_t=0
NP_t=3
NP_t=6
P_t=0
P_t=3
P_t=6
day
day

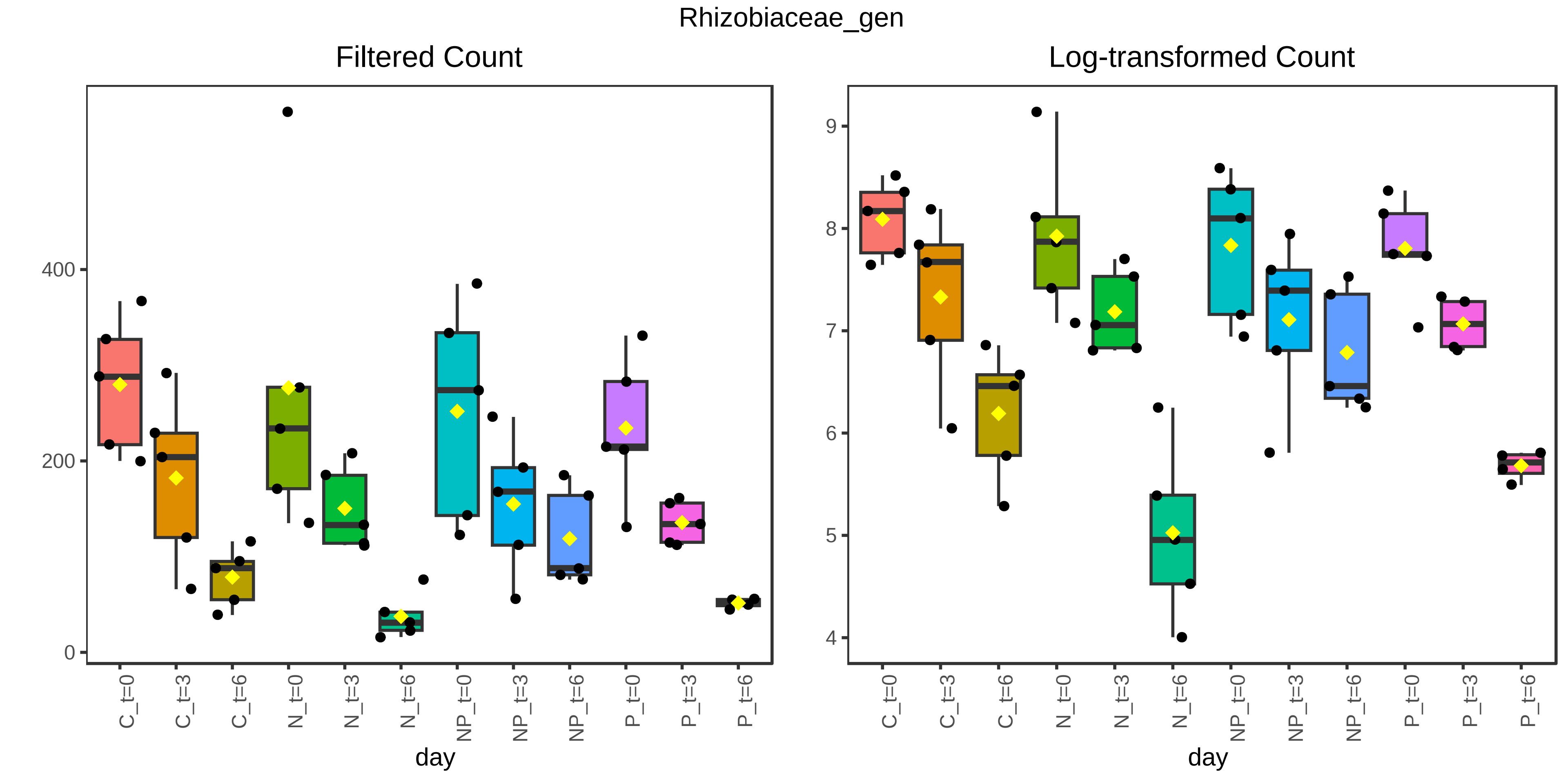
Rhizobiaceae_gen
Filtered Count
Log-transformed Count
0
200
400
4
5
6
7
8
9
C_t=0
C_t=3
C_t=6
N_t=0
N_t=3
N_t=6
NP_t=0
NP_t=3
NP_t=6
P_t=0
P_t=3
P_t=6
day

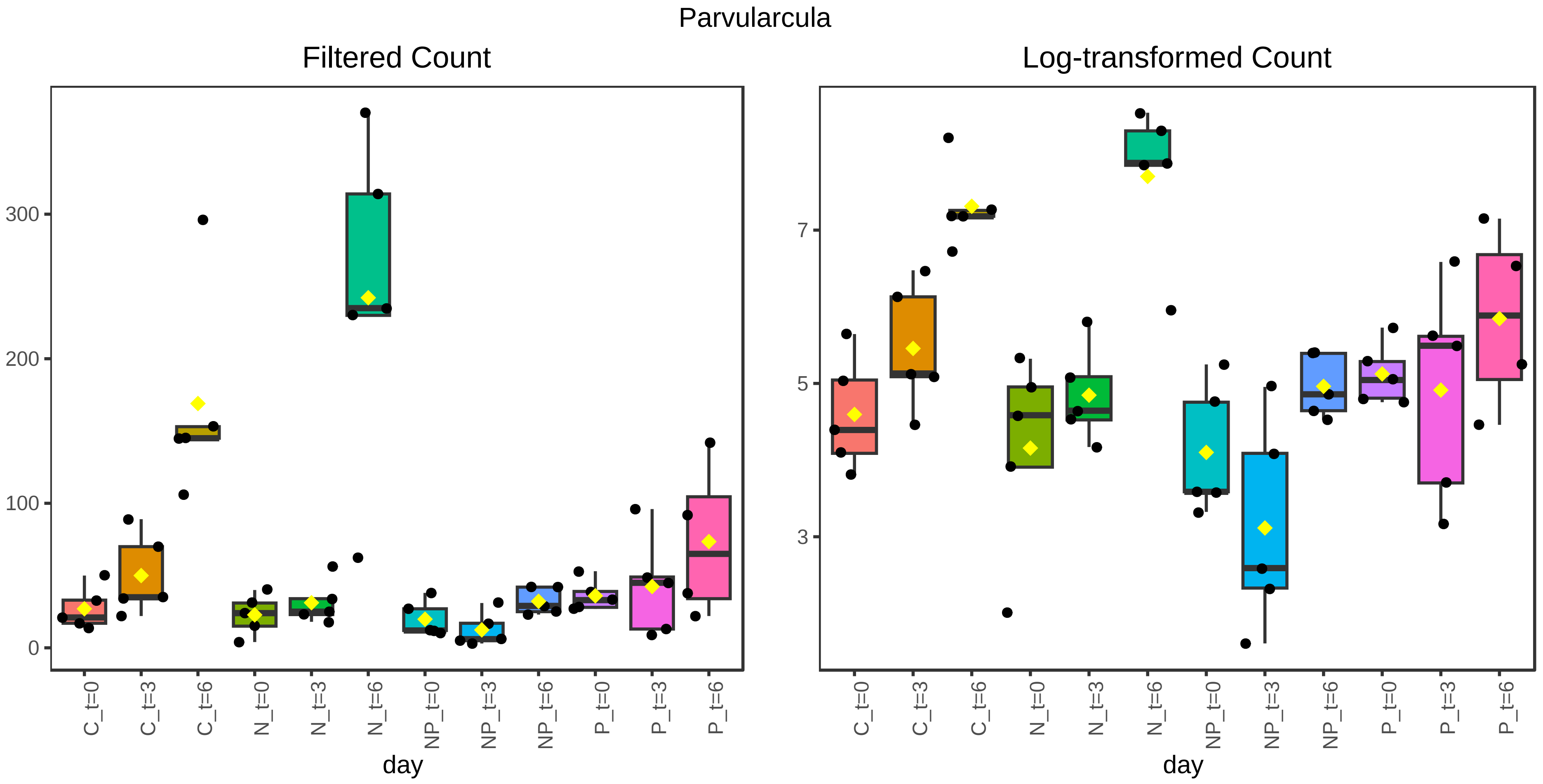

Parvularcula
Filtered Count
Log-transformed Count
300
200
100
0
7
5
3
C_t=0
C_t=3
C_t=6
N_t=0
N_t=3
N_t=6
NP_t=0
NP_t=3
NP_t=6
P_t=0
P_t=3
P_t=6
day
day

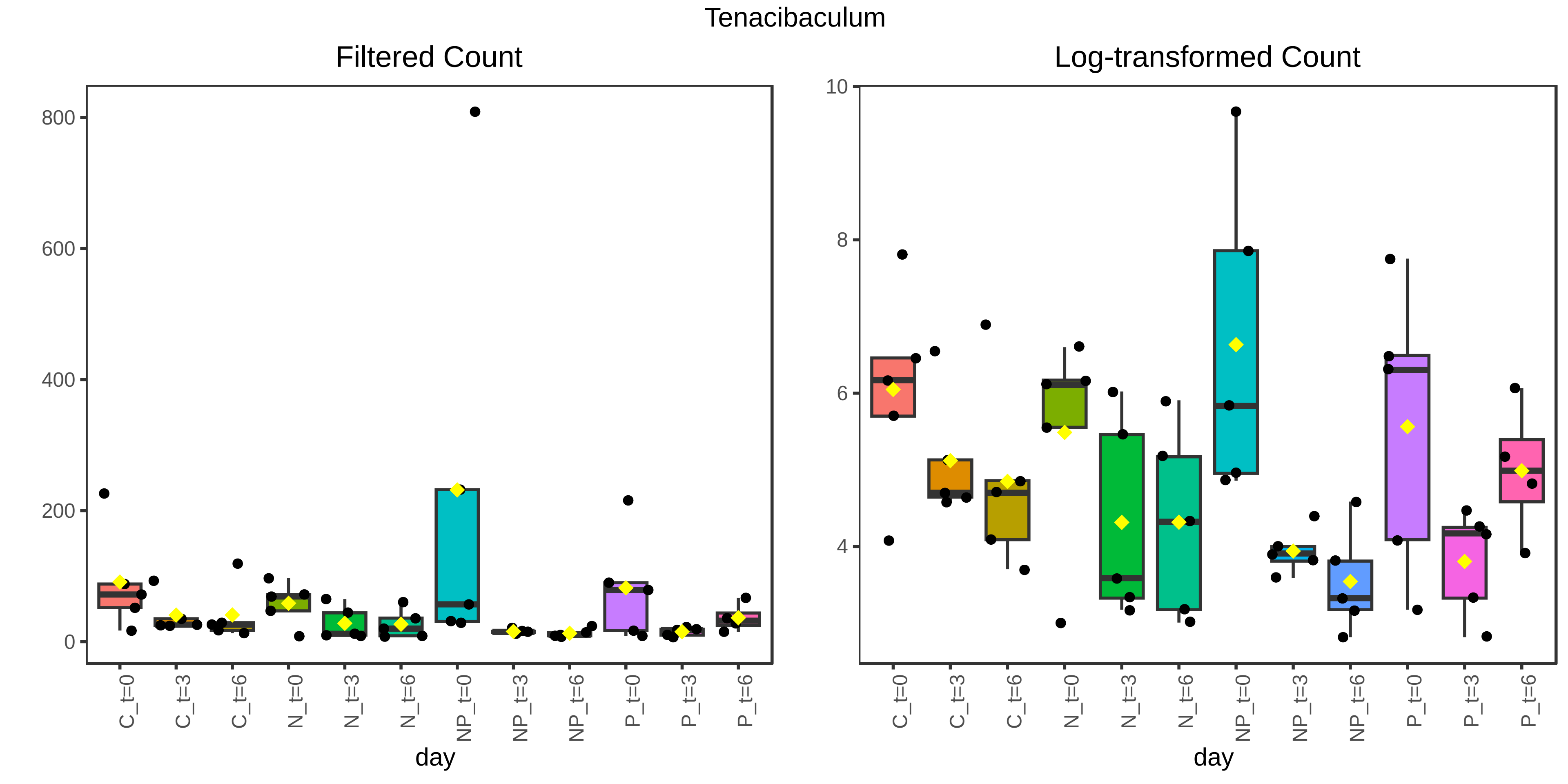

Tenacibaculum
Filtered Count
Log-transformed Count
800
600
400
200
0
10
8
6
4
C_t=0
C_t=3
C_t=6
N_t=0
N_t=3
N_t=6
NP_t=0
NP_t=3
NP_t=6
P_t=0
P_t=3
P_t=6
day
